# A synthetic lipopeptide targeting top-priority multidrug-resistant Gram-negative pathogens

**DOI:** 10.1038/s41467-022-29234-3

**Published:** 2022-03-25

**Authors:** Kade D. Roberts, Yan Zhu, Mohammad A. K. Azad, Mei-Ling Han, Jiping Wang, Lynn Wang, Heidi H. Yu, Andrew S. Horne, Jo-Anne Pinson, David Rudd, Nicolas H. Voelcker, Nitin A. Patil, Jinxin Zhao, Xukai Jiang, Jing Lu, Ke Chen, Olga Lomovskaya, Scott J. Hecker, Philip E. Thompson, Roger L. Nation, Michael N. Dudley, David C. Griffith, Tony Velkov, Jian Li

**Affiliations:** 1 Biomedicine Discovery Institute, Infection & Immunity Program and Department of Microbiology, Monash University, Melbourne, Australia; 2 Medicinal Chemistry, Monash Institute of Pharmaceutical Sciences, Faculty of Pharmacy and Pharmaceutical Sciences, Monash University, Melbourne, Australia; 3 Drug Delivery, Disposition and Dynamics, Monash Institute of Pharmaceutical Sciences, Faculty of Pharmacy and Pharmaceutical Sciences, Monash University, Melbourne, Australia; 4 Melbourne Centre for Nanofabrication, Victorian Node of the Australian National Fabrication Facility, Monash University, Melbourne, Australia; 5 Qpex Biopharma, Inc., San Diego, CA USA; 6 Present Address: Department of Biochemistry and Pharmacology, University of Melbourne, Melbourne, Australia

**Keywords:** Antimicrobial resistance, Drug discovery and development, Antibiotics, Drug development, Translational research

## Abstract

The emergence of multidrug-resistant (MDR) Gram-negative pathogens is an urgent global medical challenge. The old polymyxin lipopeptide antibiotics (polymyxin B and colistin) are often the only therapeutic option due to resistance to all other classes of antibiotics and the lean antibiotic drug development pipeline. However, polymyxin B and colistin suffer from major issues in safety (dose-limiting nephrotoxicity, acute toxicity), pharmacokinetics (poor exposure in the lungs) and efficacy (negligible activity against pulmonary infections) that have severely limited their clinical utility. Here we employ chemical biology to systematically optimize multiple non-conserved positions in the polymyxin scaffold, and successfully disconnect the therapeutic efficacy from the toxicity to develop a new synthetic lipopeptide, structurally and pharmacologically distinct from polymyxin B and colistin. This resulted in the clinical candidate **F365** (**QPX9003**) with superior safety and efficacy against lung infections caused by top-priority MDR pathogens *Pseudomonas aeruginosa*, *Acinetobacter baumannii* and *Klebsiella pneumoniae*.

## Abstract (web summary)

Polymyxins are often the last therapeutic option for multidrug-resistant (MDR) bacteria, but have suboptimal safety and efficacy. Here the authors report the discovery and development of a synthetic lipopeptide with an improved safety and efficacy against top-priority MDR Gram-negative pathogens.

## Introduction

**Fig. 1:**
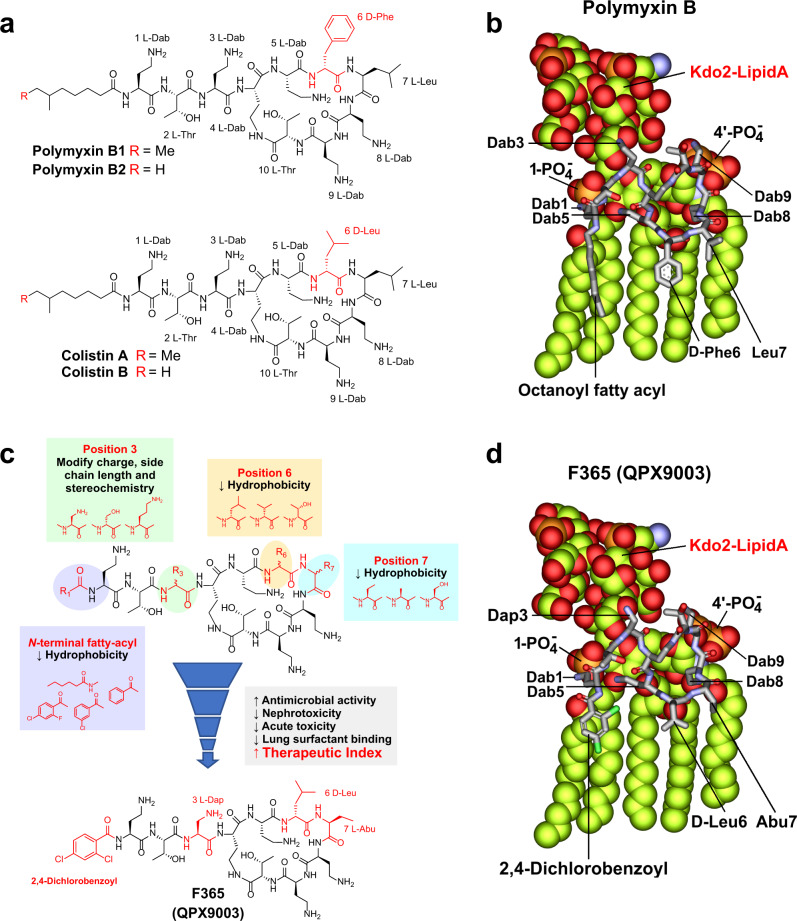
Chemical structures of the polymyxins and lipopeptide design strategy. **a** Structures of polymyxin B_1_, polymyxin B_2_, colistin A, and colistin B, the two major components in commercial polymyxin B and colistin drug products, respectively. Polymyxin B and colistin differ by only a single amino acid at position 6 (highlighted in red). The structures of the other minor components identified in commercial polymyxin B and colistin products are shown in Supplementary Table 1. **b** Molecular model of the interaction of a polymyxin B molecule with LPS showing key ionic and hydrophobic interactions of individual residues. **c** Optimization of SAR, STR, and SPR resulted in the discovery of the synthetic lipopeptide **F365** (**QPX9003**). **d** Molecular model of the interaction of the **F365** (**QPX9003**) molecule with LPS showing key ionic and hydrophobic interactions of individual residues. Although the hydrophobicity is decreased at both the N-terminus and positions 6 and 7 compared to polymyxin B, **F365** appears to retain the ability to form a similar folded conformation to polymyxin B upon interacting with the LPS. Substitution of the Dab residue at position 3 to Dap, does not appear to impact the electrostatic interaction of this position with the Kdo moiety.

Antimicrobial resistance is a major global health crisis requiring urgent attention^1,2^. Notably, hospital-acquired pneumonia and ventilator-associated pneumonia caused by multidrug-resistant (MDR) *Pseudomonas aeruginosa*, *Acinetobacter baumannii*, and Enterobacterales (e.g. *Klebsiella pneumoniae*) are major causes of morbidity and mortality^3–5^. Carbapenem-resistant isolates of these pathogens have been identified by the World Health Organization (WHO) as the top three priority pathogens to be targeted for antibiotic drug development^2^. The current dry drug development pipeline for new antibiotics has forced clinicians to resort to using the old polymyxin antibiotics (polymyxin B and colistin) as a last-line therapy against these difficult-to-treat Gram-negative pathogens^6–8^. First discovered in the 1940s, the polymyxins are a family of naturally occurring polybasic cyclic lipopeptides produced as non-ribosomal secondary metabolites by the soil bacterium *Paenibacillus polymyxa* (Fig. 1a and Supplementary Table 1)^9^. Polymyxin B and colistin (as its pro-drug colistin methansulfonate) were first introduced into clinical practice in the late 1950s^8,9^. They have very similar chemical structures, differing only by a single amino acid at position 6 in the cyclic ring of the polymyxin scaffold (Fig. 1a). Remarkably, since their introduction over 60 years ago, no new polymyxins have been approved for clinical use^9^.

The effective use of polymyxin B and colistin in the clinic is severely hampered by several major pharmacological issues. Firstly, their therapeutic window is very narrow due to significant dose-limiting nephrotoxicity, which can occur in up to 60% of patients receiving intravenous (IV) polymyxins^10,11^. Secondly, acute toxicity severely limits the maximal dose of intravenous administration^12^. Thirdly, limited exposure in the lungs following intravenous administration^13,14^ and binding to lung surfactant^15^ render them often inefficacious for pulmonary infections. Fourthly, polymyxin B and colistin products are still manufactured by fermentation as multicomponent mixtures (Fig. 1a), presenting pharmaceutical quality issues^16^. Therefore, there is an urgent need to develop new polymyxins with a much-improved therapeutic window and efficacy, in particular against pneumonia^17–21^. Here we present for the first time, a rational drug design strategy focused on concomitantly optimizing polymyxin structure-activity relationship (SAR), structure-toxicity relationship (STR), and structure-pharmacokinetic relationship (SPR, focusing on lung exposure) through modification of multiple non-conserved positions within the polymyxin scaffold (Fig. 1b). This led to the development of a synthetic lipopeptide, which has a wider therapeutic window, reduced nephrotoxicity and acute toxicity, improved drug exposure, and efficacy against lung infections compared to polymyxin B and colistin.

## Results

### Lipopeptide design strategy

Previously, we established an SAR-based mechanistic model of polymyxin antimicrobial activity built on the interaction of the polymyxin molecule with the lipid A component of LPS, the primary target of the polymyxins in the outer membrane of Gram-negative bacteria^19,21^. Here, binding of the polymyxin molecule to lipid A is characterized by a unique folded conformation wherein its N-terminal octanoyl fatty-acyl group and D-Phe^6^ and Leu^7^ make hydrophobic contacts with the hydrocarbon tails of lipid A; while the positively charged L-2,4-diaminobutyric acid (Dab) side-chains make ionic contact to the phosphates of lipid A and the ketodeoxyoctonic acid (Kdo) group. This ultimately results in displacement of divalent cations and disruption of membrane lipid packing leading to permeabilization of the outer membrane (Fig. 1b). We used this SAR-based mechanistic model to identify amino acid positions within the polymyxin scaffold that would be amenable to structural modification to modulate toxicity and lung pharmacokinetics without reducing antimicrobial activity. To this end, we identified the N-terminal fatty-acyl group, and positions 3, 6, and 7 (Fig. 1b, c), as being potential positions that can be targeted.

The amino side chain groups of Dab^1^, Dab^5^, Dab^8^, and Dab^9^ form critical ionic interactions with the 1- and 4′-phosphate groups of lipid A (Fig. 1b). These electrostatic interactions are the primary driver for initiating the interaction of the polymyxin molecule with lipid A^21^. Therefore, these four Dab residues, which are conserved residues in all native polymyxins (Supplementary Table 1), appear to be essential for antimicrobial activity and not tolerant to modification^19,21^. The hydrophobic N-terminal fatty-acyl group and residues at positions 6 and 7 form hydrophobic interactions with the fatty-acyl chains of lipid A. The Dab residue at position 3 appears to make hydrophilic contact with the Kdo sugars of lipid A (Fig. 1b)^21^. Importantly, these positions in the polymyxin scaffold are not structurally conserved in the native polymyxins (Supplementary Table 1), which potentially made them attractive sites to target for modification. At the N-terminus and positions 6 and 7, our strategy was to reduce the hydrophobicity at these sites (Fig. 1c). Conceivably, this would reduce membrane interactions with kidney tubular cells decreasing nephrotoxicity, lessen binding to lung surfactants and reduce acute toxicity. Previously, it has been shown that removing the hydrophobic N-terminal fatty-acyl group altogether from the polymyxin scaffold significantly reduces nephrotoxicity and acute toxicity^19^, but the loss of hydrophobicity significantly changes the interaction with LPS^21^ and is detrimental to antimicrobial activity^19,21^. Furthermore, while reducing the hydrophobicity of the N-terminal fatty-acyl group alone can maintain potency it does not overcome the nephrotoxicity^19^. Therefore, a possible solution would be to target a more balanced reduction of the hydrophobicity at the N-terminus as well as the hydrophobic motif at positions 6 and 7 in the polymyxin scaffold (Fig. 1c). Modeling suggested that this design strategy would potentially generate lipopeptides that retain the ability to adopt a folded conformation similar to polymyxin B upon interacting with the LPS and retain antibacterial activity (Fig. 1d). Furthermore, native polymyxin M, which contains the same N-terminal fatty-acyl groups as polymyxin B and colistin but has a less hydrophobic motif (D-Leu^6^-Thr^7^) at positions 6 and 7 (Supplementary Table 1), displayed similar LPS binding and antimicrobial potency against Gram-negative isolates to polymyxin B^21^. At position 3, we looked at removing the positive charge, changing the stereochemistry, and increasing or reducing the length of the residue side chain^22^ (Fig. 1c, d).

### Simultaneous optimization of the SAR, STR, and SPR

**Fig. 2:**
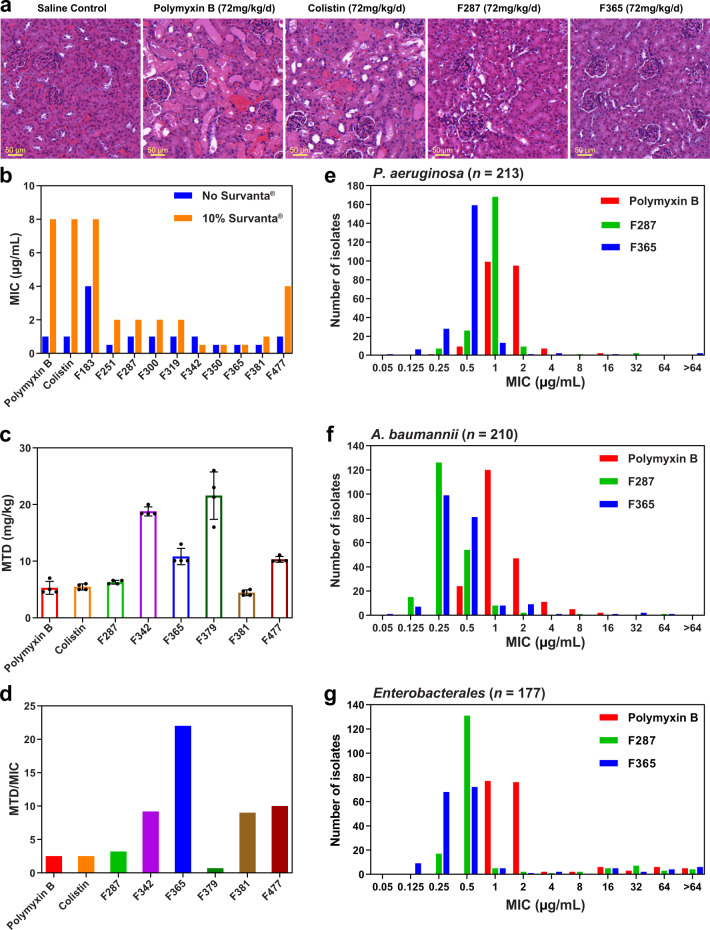
Nephrotoxicity and acute toxicity in mice and in vitro activity of polymyxin B, colistin, and synthetic lipopeptides. **a** Representative kidney histology images for saline control (*n* = 4), polymyxin B, colistin, **F287**, and **F365** treatment groups (*n* = 3) from the initial nephrotoxicity screening (Table 1). Images of saline control, **F287** and **F365** show no damage (SQS = 0) while polymyxin B and colistin caused severe damage (SQS = +5), including large tubular casts, areas of necrosis, acute cortical necrosis of tubules, and tubular degeneration. **b** MICs against *P. aeruginosa* ATCC 27853 for polymyxin B, colistin, and synthetic lipopeptides in the absence and presence of 10% Survanta®. **c** Maximum tolerated dose (MTD, mg/kg) for polymyxin B, colistin, and synthetic lipopeptides after a single IV bolus dose (*n* = 4, data are shown as mean ± s.d.). **d** Relative safety profiles for polymyxin B, colistin, and synthetic lipopeptides, measured as the ratio of average MTD to the MIC of an MDR clinical isolate *P. aeruginosa* FADDI-PA025 (an isolate in our initial screening panel that polymyxin B and colistin were the least active against, see Table 1). **e**–**g** MIC distributions of polymyxin B, **F287** and **F365** against panels of MDR clinical isolates of *P. aeruginosa* (*n* = 213, 55 carbapenem-resistant), *A. baumannii* (*n* = 210, 200 carbapenem-resistant) and *Enterobacterales* (*n* = 177, including *K. pneumoniae* [*n* = 129], *Escherichia coli* [*n* = 13]*, Enterobacter cloacae* [*n* = 27]*, K. oxytoca* [*n* = 3]*, En. aerogenes* [*n* = 1]*, Citrobacter freundii* [*n* = 4]; 152 carbapenem-resistant).

We concomitantly optimized for multiple parameters, including antimicrobial activity (against a panel of clinical MDR and ATCC reference strains, including carbapenem-resistant isolates of *P. aeruginosa*, *A. baumannii*, and *K. pneumoniae*), nephrotoxicity^16^, acute toxicity, and lung surfactant binding (Table 1, Fig. 2). An iterative design process was utilized, first looking at modifications to positions 6 and 7, followed by optimizing combinations of modifications to positions 3, 6, and 7, then the N-terminus, positions 3, 6, and 7 (Table 1). At positions 3, 6, and 7 we explored both proteogenic and non-proteogenic α-amino acid residues, while at the N-terminus we explored non-branched fatty acids and substituted aromatic-acyl groups. To generate our lipopeptide candidates a total synthesis approach was utilized using our efficient synthesis platform (Supplementary Fig. 1), which afforded lipopeptides in sufficient yields to enable preliminary in vitro and in vivo screening. Representative examples of the lipopeptides synthesized are shown in Table 1.

**Table 1:** Minimum inhibitory concentrations (MICs), in vivo efficacy and nephrotoxicity of polymyxin B, colistin, and representative synthetic lipopeptides.

					MIC (µg/mL)									
					*P. aeruginosa*			*A. baumannii*			*K. pneumoniae*			
Lipopeptide	Position				ATCC 27853	FADDI-PA025	FADDI-PA038*	ATCC 19606	FADDI-AB030*	FADDI-AB034*	FADDI-KP032*	FADDI-KP065*	Δlog_10_ CFU/mLblood**	Kidney histopathology (SQS Score)***
	N-terminus	3	6	7										
**PMB**	6-MO/7-MH	Dab	D-Phe	Leu	0.5	2	1	1	0.25	0.5	0.5	0.25	−2.69	+2 → +5 (3/3 mice)
**COL**	6-MO/7-MH	Dab	D-Leu	Leu	0.5	2	1	1	0.5	0.5	1	0.25	−2.39	+2 → +5 (3/3 mice)
**Modification of positions 6 and 7**														
**F100**	Octanoyl	Dab	D-Leu	Thr	0.5	>32	0.5	4	1	2	0.25	<0.125	−3.01	0 (3/3 mice)
**F124**	Octanoyl	Dab	D-Leu	Ala	0.5	>32	0.5	1	1	0.5	0.25	<0.125	−2.92	0 (3/3 mice)
**F224**	Octanoyl	Dab	D-Phe	Thr	1	16	0.5	1	0.25	0.5	0.5	<0.125	−3.38	0 (3/3 mice)
**F225**	Octanoyl	Dab	D-Leu	Val	1	4	1	0.5	0.25	0.25	<0.125	<0.125	−2.86	0 (3/3 mice)
**F226**	Octanoyl	Dab	D-Nle	Thr	1	16	1	2	0.5	1	<0.125	<0.125	−3.24	+1 (1/3 mice)
**F227**	Octanoyl	Dab	D-Leu	Ser	0.5	>32	>32	8	4	4	0.25	0.25	−1.04	0 (3/3 mice)
**F228**	Octanoyl	Dab	D-Thr	Leu	0.25	32	32	>32	16	32	2	16	−3.39	+1 (1/3 mice)
**F229**	Octanoyl	Dab	D-Val	Thr	>32	>32	>32	>32	32	>32	1	2	n.d.	0 (3/3 mice)
**F230**	Octanoyl	Dab	D-Thr	Thr	>32	>32	>32	>32	>32	>32	16	32	n.d.	0 (3/3 mice)
**F319**	Octanoyl	Dab	D-Leu	Abu	0.5	4	4	0.5	0.5	<0.125	0.25	<0.125	−3.44	+1 (1/3 mice)
**Modification of positions 3, 6, and 7**														
**F085**	Octanoyl	D-Dab	D-Leu	Thr	0.5	8	0.5	1	0.25	2	0.25	<0.125	−2.16	+2 (2/2 mice)†
**F183**	Octanoyl	D-Ser	D-Leu	Thr	4	>32	4	1	<0.125	0.25	<0.125	0.25	n.d.	0 (3/3 mice)
**F251**	Octanoyl	Dap	D-Leu	Thr	0.5	8	0.5	0.5	0.25	0.25	<0.125	<0.125	−1.57	0 (3/3 mice)
**F252**	Octanoyl	Orn	D-Leu	Thr	0.5	>32	2	1	0.5	1	<0.125	0.5	−1.98	+1 (2/3 mice)
**F271**	Octanoyl	Dap	D-Leu	Ala	0.5	16	1	0.5	0.25	0.25	<0.125	<0.125	−2.27	0 (3/3 mice)
**F287**	Octanoyl	Dap	D-Leu	Abu	0.5	2	0.5	0.25	0.25	<0.125	<0.125	<0.125	−2.42	0 (3/3 mice)
**F300**	Octanoyl	D-Dap	D-Leu	Thr	0.25	4	1	0.25	<0.125	<0.125	<0.125	<0.125	−1.53	+2 (3/3 mice)
**Modification of** **N-terminus positions 3, 6 and 7**														
**F314**	Nonanoyl	Dap	D-Leu	Abu	1	>32	2	0.5	0.5	2	<0.125	0.25	−2.82	+1 (3/3 mice)
**F342**	Hexanoyl	Dap	D-Leu	Abu	0.5	2	1	2	1	1	0.25	0.5	0.17	0 (3/3 mice)
**F350**	2-Chlorobenzoyl	Dap	D-Leu	Abu	0.5	4	2	8	4	4	1	0.5	n.d.	n.d.
**F360**	2,6-Dichlorobenzoyl	Dap	D-Leu	Abu	0.5	4	4	16	4	8	1	0.5	−0.07	0 (3/3 mice)
**F365**	2,4-Dichlorobenzoyl	Dap	D-Leu	Abu	0.5	0.5	0.5	0.25	0.25	0.25	<0.125	<0.125	−3.44	0 (3/3 mice)
**F371**	3-Chlorobenzoyl	Dap	D-Leu	Abu	0.5	1	2	4	1	1	0.5	1	−3.33	0 (3/3 mice)
**F378**	4-Chlorobenzoyl	Dap	D-Leu	Abu	0.5	2	1	1	1	0.25	0.25	1	−3.23	0 (3/3 mice)
**F379**	Benzoyl	Dap	D-Leu	Abu	1	>32	4	8	2	8	2	2	−2.67	n.d.
**F381**	3,5-Dichlorobenzoyl	Dap	D-Leu	Abu	0.25	0.5	1	0.5	0.25	0.25	<0.125	0.5	−3.45	+1 (1/3 mice)
**F383**	4-Methylbenzoyl	Dap	D-Leu	Abu	0.5	2	1	2	2	2	1	1	−3.02	0 (3/3 mice)
**F448**	4-Chloro-2-fluorobenzoyl	Dap	D-Leu	Abu	0.5	1	0.5	2	1	0.5	<0.125	0.25	−3.06	0 (3/3 mice)
**F449**	2-Chloro-4-fluorobenzoyl	Dap	D-Leu	Abu	0.5	2	1	4	1	1	0.25	0.25	−2.32	0 (3/3 mice)
**F477**	Biphenyl	Dap	D-Leu	Abu	1	1	1	1	0.5	0.25	0.25	0.25	−3.89	+1 (1/3 mice)

*PMB* polymyxin B, *COL* colistin, *6-MO* (*S*)-6-methyloctanoyl, *7-MH* 7-methylheptanoyl, *n.d.* not determined.

*Carbapenem-resistant isolates. ** Reduction in bacterial load of *P. aeruginosa* ATCC 27853 in blood (CFU/mL blood) at 4 h in a neutropenic mouse bloodstream infection model after a single IV dose of the lipopeptide (4 mg/kg, *n* = 3). Only lipopeptides with an MIC < 2 μg/mL against *P. aeruginosa* ATCC 27853 were tested. Δlog_10_ CFU/mL blood = log_10_ [treated] CFU/mL blood – log_10_ [control] CFU/mL blood. Mean values of three replicates are shown. ***Mouse nephrotoxicity model (*n* = 3). Lipopeptides were dosed 72 mg/kg/d subcutaneously in six divided doses every 2 h.

Semi-Quantitative Score (SQS): SQS 0 = no significant change (overall score, <1); SQS + 1 = mild damage (overall score, 1 to <15);  SQS + 2 = mild to moderate damage (overall score, 15 to <30); SQS + 3 = moderate damage (overall score, 30 to <45); SQS + 4 = moderate to severe damage (overall score, 45 to <60); and SQS + 5 = severe damage (overall score, >60)^16^.

†One mouse had to be humanely euthanized before completion of the study.

Examining modifications to the hydrophobic motif at positions 6 and 7 revealed that decreasing the hydrophobicity of the residue at position 7 generally decreased potency relative to polymyxin B and colistin, particularly against *P. aeruginosa* and *A. baumannii* isolates; with valine (**F225**) or L-2-aminobutyric acid (Abu) (**F319**) having the least impact on potency (Table 1). Reducing the hydrophobicity of position 6 alone (**F228**) or in combination with decreased hydrophobicity at position 7 (**F229** and **F230**) could result in an even greater decrease in potency relative to polymyxin B and colistin (Table 1). However, incorporation of less hydrophobic residues at position 6 (e.g. **F228**) or 7 (e.g. **F100**, **F124**, **F224**, **F225**, **F226**, **F227**, **F319**) alone or in combination (e.g. **F229**, **F230**) showed a remarkable reduction of nephrotoxicity relative to polymyxin B and colistin in our mouse model, with no nephrotoxicity being observed in most cases within the tolerated doses (Table 1, Fig. 2a). Examination of modifications to position 3 in combination with D-Leu at position 6 and less hydrophobic modifications (Thr or Abu) to position 7 (Table 1), revealed that antimicrobial activity and nephrotoxicity could be modulated by structural changes at this position. Shortening the length of the side chain of Dab^3^ through the incorporation of L-2,3-diaminopropionic acid (Dap), improved antimicrobial activity (e.g. **F100** vs **F251** and **F319** vs **F287**), consistent with previously reported data^22^. Increasing the length of the side chain at position 3 by one carbon (**F252**) did not impact potency but appeared to increase nephrotoxicity slightly (e.g. **F100** vs **F252**; Table 1). Changing the stereochemistry at position 3 from the L- to the D-configuration did not have a significant impact on potency overall against the panel of bacterial isolates, but substantially increased nephrotoxicity (e.g. **F100** vs **F085** and **F251** vs **F300**), showing that the stereochemistry of position 3 is a key structural feature of the polymyxin scaffold that influences nephrotoxicity (Table 1). This explains the nephrotoxicity of polymyxin A, the first native polymyxin to be discovered, which has a D-Dab residue at position 3 and a D-Leu^6^-Thr^7^ motif at positions 6 and 7 (Supplementary Table 1). It was never introduced into clinical practice because it was believed to be more nephrotoxic than polymyxin B and colistin^9^. Compared with polymyxin B and colistin, **F287** was the best lipopeptide of this series having comparable potency against the panel of bacterial isolates and no nephrotoxicity at a dose up to 72 mg/kg/d in our mouse model (Table 1, Fig. 2a). Finally, we optimized the N-terminus of **F287**. Replacement of the octanoyl fatty-acyl group of **F287** with a less hydrophobic C6-alkyl chain (**F342**) slightly decreased potency against several *A. baumannii* isolates (Table 1); whereas replacement with an unsubstituted phenyl group (**F379**) significantly decreased potency (>8 fold) against most isolates relative to **F287** (Table 1). Increasing the hydrophobicity of **F379** with the incorporation of an additional phenyl ring at the *para* position (**F477**) did improve potency against all isolates compared to **F379**; however, **F477** was not more active than **F287** and also resulted in slightly increased nephrotoxicity (Table 1). Monochloro substitution on the phenyl group of **F379** afforded lipopeptides with improved potency (e.g. **F371** and **F378**) relative to **F379** (Table 1). Dichloro-substitution on the phenyl ring could yield more potent lipopeptides (e.g. **F365**, **F381**) than the mono-chlorinated analogs (Table 1). **F365** and **F381** were also the most active of all the lipopeptides tested against *P. aeruginosa* FADDI-PA025 (Table 1). Notably, with the chloro-substituted analogs, the potency, nephrotoxicity, and acute toxicity were dependent on the positions of chloro-substitution (e.g. **F365** vs **F360** and **F365** vs **F381**) (Table 1 and Fig. 2c). Substitution of a chlorine atom on the phenyl ring of **F365** with fluorine at either the 2 or 4-position (e.g. **F448** and **F449**) appeared to decrease potency relative to **F365** against several isolates of *P. aeruginosa* and *A. baumannii* (Table 1). Overall, **F365** was the best of this series of N-terminal analogs having similar potency to **F287** (slightly better than polymyxin B and colistin) and no nephrotoxicity even at a dose up to 72 mg/kg/d in our mouse model (Fig. 2a). Subsequently, we evaluated the efficacy of the potential lead lipopeptides in a neutropenic mouse bloodstream infection model developed by our group^16^ to allow for rapid screening of large numbers of lipopeptides (Table 1). Several of our lipopeptides (e.g. **F365**, **F371**, **F381**, and **F477**) showed a greater reduction in the bacterial burden in blood than polymyxin B and colistin (Table 1).

To predict the in vivo efficacy against lung infections, the in vitro antibacterial activity of our lipopeptides was further examined in the presence of Survanta^®^, a natural bovine lung surfactant extract that has been used to examine the potential lung surfactant binding of lipopeptides in antibiotic development^23^. MICs of both polymyxin B and colistin increased 8-fold in the presence of the lung surfactant; whereas the MICs of our synthetic lipopeptides were not impacted (e.g. **F365**) or slightly impacted (e.g. **F251**) in the presence of the lung surfactant (Fig. 2b). It appears that position 7 is a driver of lung surfactant binding of polymyxin B and colistin, as substitution with a less hydrophobic residue at position 7 helped to restore activity in the presence of lung surfactant (e.g. polymyxin B/colistin vs **F319** in Fig. 2b). The N-terminus also had an impact on lung surfactant binding, which appeared to be dependent on the hydrophobicity of the N-terminal group, e.g. **F477** (N-terminal biphenyl [C12], D-Leu^6^-Abu^7^) vs **F342** (N-terminal hexanoyl [C6], D-Leu^6^-Abu^7^). Modification to position 3 did not appear to have any significant influence on binding to lung surfactant (e.g. **F287** vs **F319** and **F183** vs **F251**, Fig. 2b). Together, the results here indicate that the combined overall hydrophobicity of the position 7 residue and N-terminal group is the primary driver of lung surfactant binding. In a mouse, acute toxicity model, both polymyxin B, and colistin had an average maximum tolerated dose (MTD) of 5.4 and 5.3 mg/kg respectively, while our lipopeptides achieved up to a 4-fold increase in the MTD relative to polymyxin B and colistin (Fig. 2c). Our results clearly show that the N-terminal fatty-acyl group is the primary structural component of the polymyxin scaffold that drives the acute toxicity (e.g. **F287** vs **F342** and **F379**, Fig. 2c), with position 3 and positions 6/7 having less impact (e.g. **F287** vs polymyxin B, Fig. 2c). Using the ratio of MTD/MIC as an indicator of the relative safety (Fig. 2d), it is evident that these lipopeptides have the potential for much better safety than polymyxin B and colistin, with **F365** being a stand-out candidate.

### Selection of a lead candidate with a significantly improved safety profile

The therapeutic potential of **F287** and **F365** was further evaluated to select a lead candidate to move forward. To this end, extended MIC screening was conducted against a large panel (*n* = 600) of MDR clinical isolates, including 407 carbapenem-resistant isolates (Fig. 2e–g). Specifically, against *P. aeruginosa* (*n* = 213), **F365** (MIC_50_/MIC_90_, 0.5/1 μg/mL) was 2-fold more potent than polymyxin B (MIC_50_/MIC_90_, 1/2 μg/mL) and slightly more potent than **F287** (MIC_50_/MIC_90_, 1/1 μg/mL). Against *A. baumannii* (*n* = 210), **F287** (MIC_50_/MIC_90_, 0.25/0.5 μg/mL) and **F365** (MIC_50_/MIC_90_, 0.25/1 μg/mL) were up to 8-fold and 4-fold more potent than polymyxin B (MIC_50_/MIC_90_, 1/4 μg/mL), respectively. In addition, a 7-day in vitro passaging study showed that resistance to polymyxin B, colistin, **F287**, and **F365** at 4× MIC emerged within 3 days; while at 16× MIC, regrowth was observed from day 3 and 4 for polymyxin B and colistin respectively; but not for **F287** and **F365** (Supplementary Fig. 2), indicating a lower potential of resistance for both lipopeptides than polymyxin B and colistin. No growth was observed over 7 days with polymyxin B, colistin, **F287**, or **F365** at 32× MIC. Overall, **F287** and **F365** had very similar in vitro antimicrobial activity profiles. To gauge their potential therapeutic windows, **F287** and **F365** were administered intraperitoneally (IP) in mice at a total dose of 120 mg/kg (as 6 divided doses every 2 h). At 20 h after the last dose, **F365** showed no damage to the kidneys, similar to the saline control, whereas mice treated with **F287** at the same dose, showed minor signs of damage to the kidneys (Supplementary Table 2). The nephrotoxicity profile of **F365** was further evaluated in mice at a higher total dose of 150 mg/kg IP with treated mice showing no significant damage to the kidneys, similar to the saline control (Supplementary Fig. 3). Considering the in vitro potency and the higher dose achieved without observing nephrotoxicity, it was evident that **F365** had a greater relative safety than **F287** and therefore was selected as the lead candidate to progress forward.

### Efficacy and pharmacokinetics of F365

**Fig. 3:**
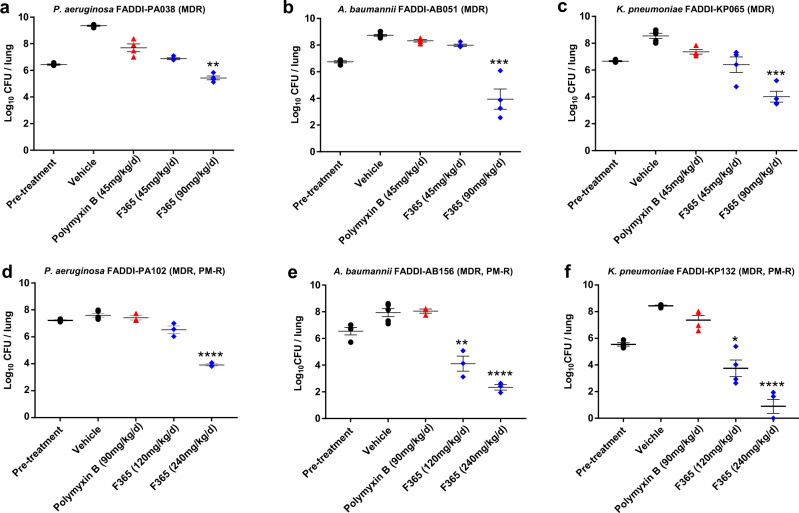
Efficacy of F365 and polymyxin B in a neutropenic mouse pneumonia model against polymyxin-susceptible (a–c) and polymyxin-resistant (PM-R) (d–e) MDR clinical isolates. **a** *P. aeruginosa* FADDI-PA038 (**F365** MIC = 0.5 μg/mL, polymyxin B MIC = 0.5 μg/mL; *n* = 4). **b** *A. baumannii* FADDI-AB051 (carbapenem-resistant, **F365** and polymyxin B MIC = 0.5 μg/mL; *n* = 4). **c** *K. pneumoniae* FADDI-KP065 (carbapenem-resistant, **F365** MIC  <0.125 μg/mL, polymyxin B MIC = 0.25 μg/mL; *n* = 4). **d** *P. aeruginosa* FADDI-PA102 (carbapenem-resistant, **F365** and polymyxin B MIC = 4 μg/mL; *n* = 3). **e** *A. baumannii* FADDI-AB156 (carbapenem-resistant, **F365** MIC = 4 μg/mL, polymyxin B MIC = 8 μg/mL; *n* = 3). **f** *K. pneumoniae* FADDI-KP132 (*mcr-1* positive, **F365** and polymyxin B MIC = 8 μg/mL; *n* = 4). **F365**, polymyxin B or the vehicle was administered (in three divided doses) intraperitoneally (**a**–**c**) or subcutaneously (**d**–**f**) every 8 h over 24 h. Data are shown as mean ± s.e.m. Two-tailed one-way ANOVA with Tukey HSD post-hoc test (FDR adjustment for multiple comparisons) was used to compare different groups. Statistical details are provided as a Source Data file. FDR-adjusted *P*-values ^*^P < 0.05, ^**^P < 0.01^, ***^P < 0.001, ^****^P < 0.0001 relative to the pre-treatment group. EUCAST breakpoints of colistin were employed: Susceptible ≤ 2 μg/mL, Resistant > 2 μg/mL.

The in vivo efficacy of **F365** was firstly examined in a neutropenic mouse pneumonia model against polymyxin-susceptible MDR clinical isolates of *P. aeruginosa*, *A. baumannii*, and *K. pneumoniae*, which also included carbapenem-resistant isolates (Fig. 3a–c). At 45 mg/kg/d, the highest IP dose that could be safely administered for polymyxin B, both polymyxin B and **F365** did not show any significant killing at 24 h (compared to the pre-treatment bacterial inoculum) against the polymyxin-susceptible MDR clinical isolates (Fig. 3a–c). However, the improved acute tolerability of **F365** allowed us to safely increase the IP dose to 90 mg/kg/d to achieve bacterial killing at 24 h against *P. aeruginosa*, *A. baumannii*, and *K. pneumoniae* (Fig. 3a–c). In a neutropenic mouse thigh infection model against a polymyxin-susceptible carbapenem-resistant *A. baumannii* clinical isolate FADDI-AB30, **F365** also showed >2.0 log_10_ reduction in CFU/thigh than polymyxin B (Supplementary Fig. 4). Considering the superior safety profile of **F365** and its lack of binding to lung surfactant, we further examined its efficacy against polymyxin-resistant isolates of *P. aeruginosa, A. baumannii*, and *mcr-1* positive *K. pneumoniae* in the neutropenic mouse pneumonia model (Fig. 3d–f; MICs are shown in Supplementary Table 3). Pleasingly, **F365** (240 mg/kg/d subcutaneously) displayed significant bacterial killing (up to 4.65 log_10_ reduction in CFU/lung) against the polymyxin-resistant isolates, highlighting the advantage of the increased therapeutic index and lower lung surfactant binding of **F365** to achieve efficacious drug exposure at the infection site.

**Fig. 4:**
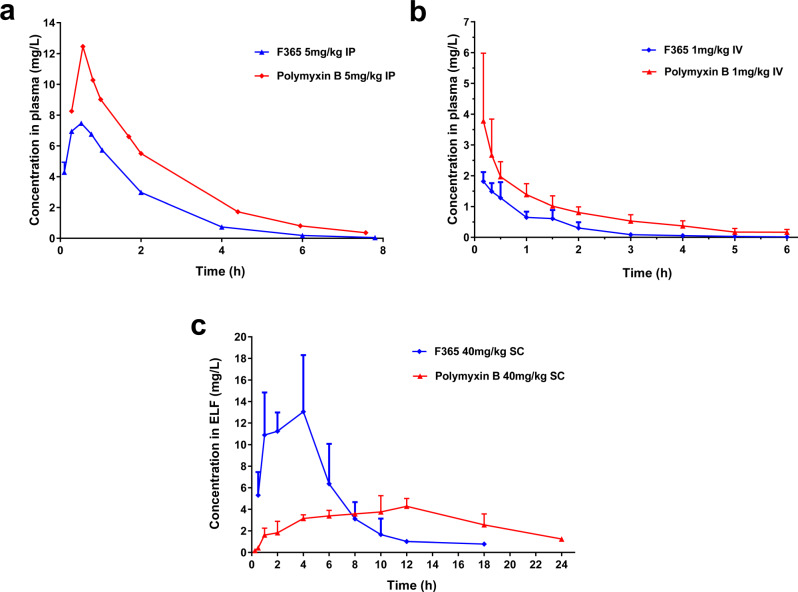
Pharmacokinetics of **F365** and polymyxin B in rodents. **a** Plasma concentration versus time profiles of **F365** and polymyxin B (*n* = 3 mice) following an intraperitoneal dose (5 mg/kg) in mice. **b** Plasma concentration versus time profiles of **F365** (*n* = 5 rats) and polymyxin B (*n* = 11 rats) following an intravenous dose (1 mg/kg) in rats. **c** ELF concentration versus time profiles following a subcutaneous dose (40 mg/kg) in mice (*n* = 3 mice). All data are shown as mean ± s.d.

Protein binding of **F365** in mouse (36%), rat (33%), and human (45%) plasma were similar and substantially lower than polymyxin B (95%, 80%, and 68%, respectively). The pharmacokinetics of **F365** and polymyxin B in both mice and rats showed that **F365** had a higher total body clearance and shorter half-life than polymyxin B (Fig. 4a, b and Supplementary Table 4). However, **F365** had a higher free (unbound) drug exposure (*f*AUC) in plasma than polymyxin B in mice at 5 mg/kg and also in rats at 1 mg/kg. In rats we observed higher (>4-fold) urinary recovery for **F365** than polymyxin B, suggesting decreased reabsorption of **F365** by the kidneys (Supplementary Table 4). We further examined the pharmacokinetics of **F365** and polymyxin B in mouse pulmonary epithelial lining fluid (ELF) (Supplementary Table 4 and Fig. 4c). After a single subcutaneous dose of 40 mg/kg, **F365** and polymyxin B had very different concentration-time profiles, with **F365** having a 3-fold higher C_max,_ than polymyxin B. While the AUC_ELF_ of **F365** (86.2 mg·h/L) was comparable to that of polymyxin B (80.0 mg·h/L), based on the marked loss of antibacterial activity (87.5%) of polymyxin B due to binding to lung surfactant (Fig. 2b), the estimated *f*AUC_ELF_ of **F365** would be approximately 8-fold higher than that for polymyxin B (Supplementary Table 4). Collectively, our results demonstrated significantly different pharmacokinetics in the lungs, which explains the much-improved efficacy of **F365** in the mouse pneumonia model, including against polymyxin-resistant isolates with polymyxin B MICs of 4–8 μg/mL.

### Mechanisms of improved activity and reduced nephrotoxicity

**Fig. 5:**
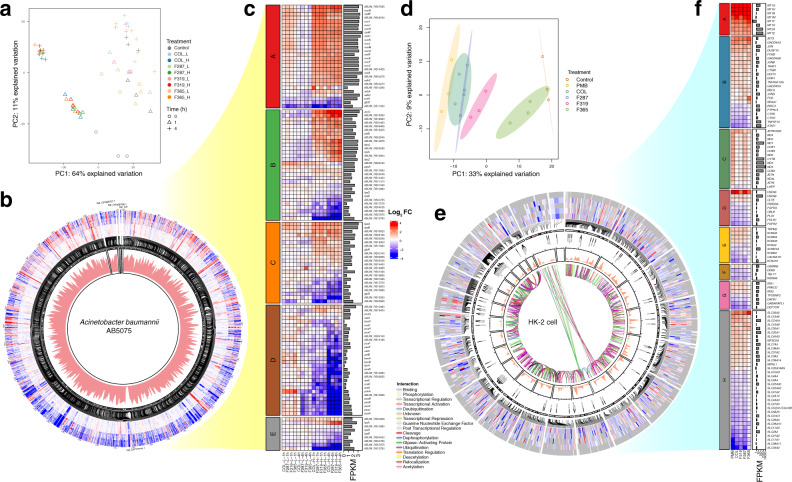
Gene expression changes in *A. baumannii* AB5075 and HK-2 following treatments with polymyxin B (PMB, HK-2 only), colistin (COL), **F287**, **F319** and **F365**. **a** PCA plot of gene expression in *A. baumannii* AB5075. **b** Gene expression changes in *A. baumannii* AB5075 following treatments with colistin and synthetic lipopeptides at low (L) and high (H) concentrations at 1 and 4 h (*n* = 3 biologically independent samples). Data from innermost-to-outermost circles are the average values of normalized read abundance (FPKM) under all treatments, gene names, and heatmap of gene expression changes (linear model and empirical Bayes statistics for differential expression in limma package, fold change ≥ 2, FDR-adjusted *P*-value ≤ 0.05). Enlarged for details. **c** Heatmap of key gene expression changes and average values of normalized read abundance (FPKM, in log_10_ scale) under all treatments. Groups of genes: (A) respiration; (B) fatty acid biosynthesis; (C) fatty acid degradation; (D) aromatic compound degradation; (E) lipid A biosynthesis. **d** PCA plot of gene expression in HK-2. **e** Gene expression changes in HK-2 cells following treatments with colistin and synthetic lipopeptides at 24 h (*n* = 3 biologically independent samples). Data from innermost-to-outermost circles are gene regulation pairs from Signor database, averaged read abundance (FPKM), critical genes of polymyxin toxicity identified by previous CRISPR screening^27^, chromosome cytotypes, and heatmap of gene expression changes (linear model and empirical Bayes statistics for differential expression in limma package, fold change ≥ 1.5, FDR-adjusted *P*-value < 0.05) under treatments with polymyxin B, colistin, **F287**, **F319**, and **F365**, with insignificant genes indicated by gray. Enlarged for detail. **f** Heatmap of key gene expression changes and averaged values of normalized read abundance (FPKM, in log_10_ scale) under all treatments. Groups of genes: (A) metallothionein; (B) apoptosis; (C) mitochondrial respiratory chain; (D) endocytosis; (E) ion channels; (F) ubiquitination; (G) cell proliferation; (H) SLC transporter family. Fig. 5c, f share the same Log_2_FC scale bar. Source data are provided as a Source Data file.

Polymyxins exert their antibacterial effect by disrupting the Gram-negative outer membrane^21^. We demonstrated that **F365** was able to permeabilize the outer membrane of *P. aeruginosa* and inhibit efflux to a greater extent than polymyxin B (Supplementary Fig. 5). We also conducted transcriptomics analyses to examine the mechanism underlying improved potency of **F365**. Treatments of *A. baumannii* AB5075 with **F365**, **F287**, **F319**, and colistin induced significant perturbations to bacterial gene expression in a concentration- and time-dependent manner (Fig. 5a, b). A low concentration (2 μg/mL) of all lipopeptides resulted in enhanced cell envelope biogenesis, expression of membrane transporters, fatty acid degradation, and arginine metabolism, and reduced trehalose biosynthesis (Fig. 5c). At the high concentration (8 μg/mL), all lipopeptides caused extensive transcriptomic perturbations, with approximately 33% and 50% of the genome differentially expressed at 1 and 4 h, respectively (Fig. 5b); particularly upregulated biosynthesis of major cell constituents, carbohydrate, and energy metabolism; and downregulated lipid A biosynthesis (*lpxC, lpxH, lpxB*). Notably, at 8 μg/mL **F365** caused the highest upregulation of fatty acid biosynthesis genes (e.g. *fabG, fabZ*, *fabD*) (Fig. 5c), indicating that **F365** caused greater membrane destabilization consistent with its increased potency (Table 1, Fig. 2e–g).

**Fig. 6:**
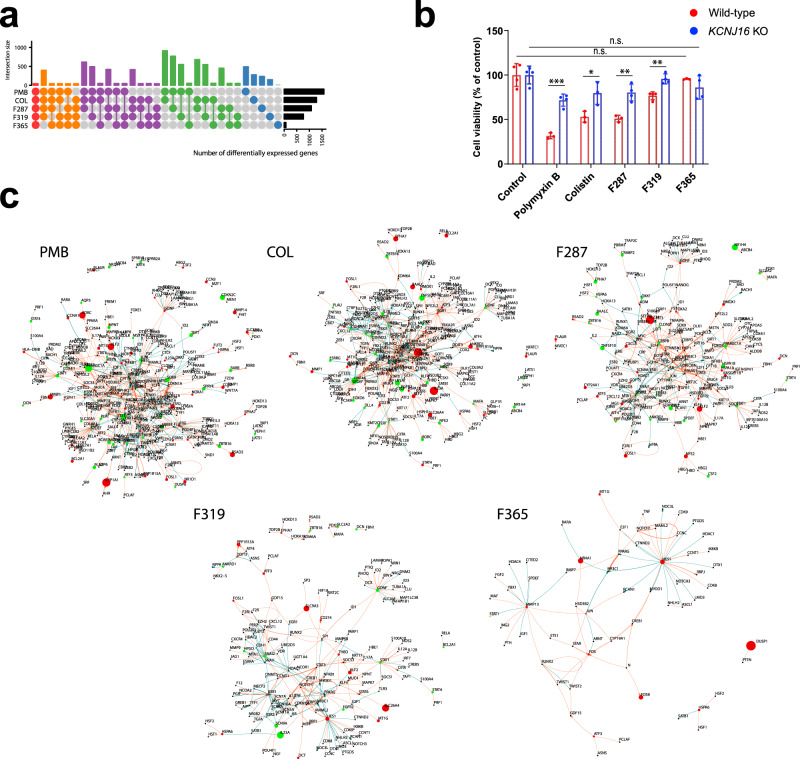
Transcriptomic changes in human kidney proximal tubular HK-2 cells following treatments with polymyxin B (PMB), colistin (COL), **F287**, **F319**, and **F365**. **a** Numbers of differentially expressed genes. Distinctive intersections are color-coded. **b** Cell viability difference (two-tailed Student’s *t*-test, *n* = 4 biologically independent cells, Benjamini–Hochberg adjusted *P*-values) between wild-type HK-2 and *KCNJ16* knockout (KO) cells following 24 h treatment with lipopeptides. n.s., not significant; ^*^adjusted *P* < 0.05, ^**^adjusted *P* < 0.01, ^***^adjusted *P* < 0.001. Data are shown as mean ± s.d. and individual data points. **c** Extracted transcriptional regulatory network from Signor database with 1-step neighbor constraints (enlarged for detail). Source data are provided as a Source Data file.

Polymyxin nephrotoxicity is associated with high levels of accumulation in renal proximal tubular cells in which transporters (megalin/PEPT/OCTN2) and endocytosis play key roles^24^. We have demonstrated that intracellular accumulation of polymyxin B and colistin results in oxidative stress and apoptosis^25^. Here we examined the impact of the synthetic lipopeptides **F365**, **F287**, **F319**, polymyxin B, and colistin on the transcriptomes of human HK-2 cells (Fig. 5d–f). At 100 μM, polymyxin B, colistin, **F287**, and **F319** significantly changed the expression of 1576, 1282, 1076, and 763 genes, respectively, while **F365** only changed the expression of 70 genes (Fig. 6a). Gene expression following treatment with polymyxin B, colistin, **F287**, or **F319** are close to each other, but are well segregated from **F365** and the untreated control in the Principal Component Analysis plot (Fig. 5d). Consistently, **F365** had a relatively low similarity (0.78-0.82, semantic analysis) of perturbed gene ontology compared with polymyxin B, colistin, **F287**, and **F319**. Polymyxin B and colistin caused the most significant transcriptomic perturbations (Fig. 5e). Notably, all lipopeptides except **F365** altered the expression of potassium channels (Fig. 5f), including *KCNJ16* (inwardly rectifying potassium channel Kir5.1). Furthermore, **F365** had no effect on cell viability of HK-2 cells or *KCNJ16* knockout, while *KCNJ16* knockout protected HK-2 cells against 24 h treatment with all other lipopeptides (Fig. 6b). These results are consistent with our recent CRISPR screen findings where *KCNJ16* was top-ranked in mediating polymyxin-induced toxicity in HK-2 cells^26^. All lipopeptides except **F365** repressed the expression of key endocytic genes (Fig. 5f), but upregulated HSC70 chaperone genes associated with clathrin coat disassembly. Overall, **F365** caused minimum perturbations to HK-2 transcriptome compared to polymyxin B, colistin, **F287**, and **F319** (Fig. 6c), consistent with its significantly reduced nephrotoxicity.

**Fig. 7:**
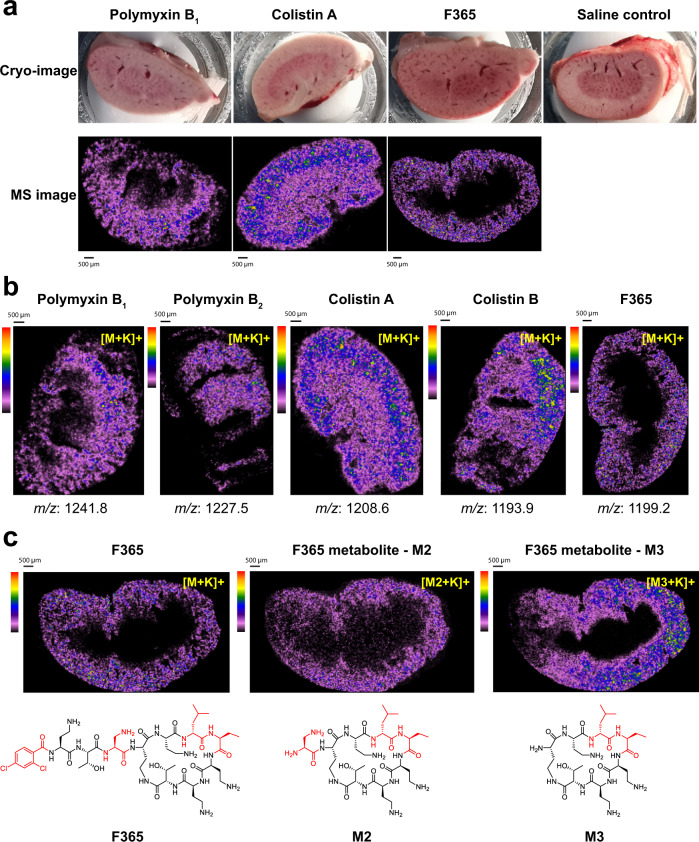
ME-SALDI-MS imaging of polymyxin distribution, accumulation, and metabolism in mouse kidneys. Mice (*n* = 3) were administered lipopeptides (10 μmol/kg) subcutaneously, 6 doses (2-h interval) on Day 1 and 3 doses (2-h interval) on Day 2. Histological examination revealed that all the kidneys exposed to polymyxin B_1_, polymyxin B_2_, colistin A, and colistin B had severe microscopic damage with SQS scores up to +5; whereas the kidneys exposed to **F365** showed no significant microscopic damage and were not graded for damage. **a** Representative cryo-images of mouse kidney tissue sections after treatment with polymyxin B_1_, colistin A, **F365**, and saline control; and the corresponding ME-SALDI-MS image of the tissue sections after scanning for the parent ions [M + K]^+^ for polymyxin B_1_, colistin A, **F365**, respectively. **b** Representative ME-SALDI-MS images highlighting the distribution and accumulation of polymyxin B_1_, polymyxin B_2_, colistin A, colistin B, and **F365** in mouse kidney tissue. **c** Representative ME-SALDI-MS images showing the distribution and accumulation of **F365** and its metabolites **M2** and **M3** in mouse kidney tissue after treatment. All ME-SALDI-MS images were normalized to total ion count based on the highest intensity peak across each tissue section and its corresponding concentration curve. Selected lipopeptide-related ions are displayed with a scale between 0 and 40% relative to the highest intensity peak in the summed spectrum of the whole tissue. A default color intensity gradient is used to visualize and differentiate low concentrations (purple to blue), mid-range concentrations (green to yellow), and high concentrations (orange to red) of lipopeptide disposition.

Matrix enhanced-surface-assisted laser desorption/ionization mass spectrometry (ME-SALDI-MS) imaging revealed very different distribution, accumulation, and metabolism of **F365** compared to polymyxin B and colistin in mouse kidney tissues (Fig. 7). MS signals corresponding to the [M + K]^+^ and [M + Na]^+^ molecular ions for each lipopeptide were observed in the kidney tissue samples. Polymyxin B_1_/B_2_ and colistin A/B had a similar level of distribution in both cortical and medullary regions; whereas **F365** was primarily in the cortex with very little medullary distribution (Fig. 7a, b). Concentrations of polymyxin B_1_/B_2_ (38.2 and 70.9 µM, respectively) and colistin A/B (86.7 and 140.8 µM) in the cortex region were ~2–7 fold higher than **F365** (20.8 µM) (Fig. 7b). Ion signals were observed for putative metabolites (**M1**, **M2**, **M3**) of polymyxin B_1_, colistin, and **F365** that lacked the *N*-terminal region (Fig. 7c, Supplementary Figs. 6, 7). Polymyxin B_2_ and colistin B appeared to undergo less metabolism than polymyxin B_1_ and colistin A, respectively (Supplementary Fig. 7). This potentially explains the differences in the concentrations observed here for polymyxin B_1_/B_2_ and colistin A/B in the kidney tissue. Metabolism of **F365** in the kidneys differed from polymyxin B and colistin, with **M3** being the major metabolite and no **M1** metabolite detected (Fig. 7c and Supplementary Fig. 7). Collectively, the pharmacokinetic and mechanistic results show that the very low potential for nephrotoxicity of **F365** is a combination of less kidney reabsorption (and accumulation in tubular cells) and less intracellular toxicity.

### F365 safety in a primate model

To further evaluate the safety of **F365**, a 14-day repeat-dose GLP toxicology study was undertaken in cynomolgus monkeys (Supplementary Table 5). **F365** was well tolerated with no adverse clinical signs, including signs of acute toxicity, observed at any dose level. Kidney histopathological results revealed minimal tubular degeneration in 2 of 8 monkeys at the dose of 20 mg/kg/day; and minimal (4 of 8 monkeys), mild (1 of 8 monkeys), and moderate (1 of 8 monkeys) tubular degeneration at the highest examined dose of 50 mg/kg/day. Histopathological changes correlated with elevated serum creatinine and urea in 2 of 8 monkeys that received 50 mg/kg/day for 14 days. From these findings above, the No-Observed-Adverse-Effect-Level (NOAEL) for **F365** was considered to be at least 20 mg/kg/day. It has been previously reported that polymyxin B administered to female cynomolgus monkeys at 3.8 mg/kg/12 h (7.6 mg/kg/day) for a shorter time period of 7 days, showed increased histological damage in all test animals along with mild increases in serum creatinine and blood urea nitrogen concentrations^27^. In patients, the maximum recommended dose for intravenous polymyxin B is 2.5 mg/kg/day, with nephrotoxicity and acute toxicity being observed at these doses^12,28–30^. Based on allometric scaling and the No-Observed-Adverse-Effect-Level (NOAEL)^31^ in monkeys of 20 mg/kg/day from our 14-day GLP toxicity study of **F365**, it is estimated that **F365** can be safely administered to patients at a dose of at least 6.5 mg/kg/day without any acute toxicity or nephrotoxicity.

## Discussion

Developing safer, potent polymyxin lipopeptides is very challenging due to the complex interrelationships between structure and antibacterial activity, nephrotoxicity, acute toxicity, lung drug exposure, and lung surfactant binding^19^. There have been efforts to address their pharmacological liabilities with the development of new polymyxin analogs derived from polymyxin B and colistin^8,17–22,32–34^. While many novel polymyxin B and colistin derivatives have been generated using this approach, very few have entered clinical evaluation^19,35^. The polymyxin B analog SPR206 has successfully completed a Phase 1 study of both single- and multipleascending dose cohorts^35^. While no nephrotoxicity was observed at up to 100 mg q8h in the multiple-ascending dose cohort, neurotoxicity at single doses exceeding 200 mg was observed^35^. These drug development efforts have primarily focused on modification of the N-terminus of the polymyxin B and colistin scaffold with very limited exploration of the remaining lipopeptide scaffold, especially the cyclic ring^19,21^. Furthermore, previous polymyxin drug discovery programs have traditionally relied on cell-based assays using human kidney cells to screen for nephrotoxicity. However, there is a growing consensus that the results from these in vitro cytotoxicity assays do not translate to in vivo models and are not able to reliably differentiate the nephrotoxicity of different polymyxins^16,22,32^. To address this, our group was the first to develop a mouse model to screen for potential nephrotoxicity^16^, which was also utilized in this work. Finally, while previous polymyxin drug discovery efforts have focused on trying to ameliorate nephrotoxicity, this has been done without also targeting acute toxicity and the high lung surfactant binding.

Our approach of systematically modifying multiple positions throughout the polymyxin scaffold, while optimizing for multiple parameters (antibacterial activity, nephrotoxicity, acute toxicity, lung drug exposure, and lung surfactant binding) concomitantly, represents a different strategy for the discovery of new polymyxin lipopeptides. This is the first time that positions 6 and 7 in the polymyxin scaffold have been specifically targeted to modulate their pharmacological properties. As a result, we identified several new structural hotspots driving antimicrobial activity (positions 6 and 7), nephrotoxicity (positions 3, 6, and 7), acute toxicity (N-terminal fatty-acyl group), and lung surfactant binding (N-terminus and position 7). Additionally, our results highlight how narrow the chemical space can be for exploring modifications to the polymyxin scaffold when optimizing for multiple parameters simultaneously. Optimizing the combination of modifications at all the positions targeted (N-terminus, positions 3, 6, and 7) was critical for providing lipopeptides with the best balance between potency and toxicity. Subtle structural changes to individual positions of the lipopeptide scaffold could cause pronounced changes to activity and toxicity. This was nicely exemplified by simply changing the positions of the chloro-substituents in the phenyl ring at the N-terminus, whereby going from the 2,4-dichloro substitution (**F365**) to the 2,6-dichloro substitution (**F360**), resulted in a significant decrease in potency, but did not impact nephrotoxicity (**F360** vs **F365** in Table 1). However, going from the 2,4-dichloro substitution (**F365**) to the 3,5-dichloro substitution (**F381**) maintained potency but increased nephrotoxicity and acute toxicity (**F365** vs **F381** Table 1 and Fig. 2c). Similarly, switching of the stereochemistry of a Dab or Dap residue at position 3 from the L- to D-stereoisomer led to increased nephrotoxicity, without impacting potency (e.g. **F085** vs **F100** and **F251** vs **F300** in Table 1). The findings here indicate that along with hydrophobicity, molecular conformation may also play an important role in polymyxin activity and toxicities.

Importantly, for the first time, we have been able to structurally disconnect the antimicrobial activity from nephrotoxicity, acute toxicity, and lung surfactant binding to develop a synthetic lipopeptide that is pharmacologically distinct from the existing polymyxin drugs. The improved activity, safety, and minimal lung-surfactant binding of **F365** allowed us to achieve sufficient drug exposure following parenteral administration to target lung infections, even those caused by polymyxin-resistant Gram-negative isolates. Considering the increased potency and efficacy, decreased acute toxicity and nephrotoxicity, and the higher unbound lung exposure, **F365** represents a promising synthetic lipopeptide drug candidate for targeting problematic carbapenem-resistant pulmonary infections in humans. To this end, **F365**, now known as **QPX9003**, has completed IND-enabling studies and has commenced Phase 1 clinical development (NCT04808414).

## Methods

### Materials

Polymyxin B sulfate and colistin sulfate were obtained from BetaPharm (Shanghai, China). The isolation of purified polymyxin B_1_ and polymyxin B_2_, colistin A and B from commercial preparations of polymyxin B and colistin, respectively, was conducted as previously described^16^.

### Modeling of the polymyxin B and F365-Lipid A complex

The methodology used for the construction of the models of polymyxin B and **F365** in complex with Kdo-lipid has been previously described by our group^36^.

### Lipopeptide synthesis

The general methodology used for the preparation of the synthetic lipopeptides has been previously reported by our group^37^. A full description of the materials used, synthesis protocol, and analytical data obtained are presented in the Supplementary Information.

### Measurements of minimum inhibitory concentrations (MICs)

MICs were determined in cation-adjusted Mueller–Hinton broth (CaMHB) by the broth micro-dilution method against ATCC strains and clinical isolates^16^. MICs in the presence of Survanta^®^ were determined as described above against *P. aeruginosa* ATCC 27853. In the 100 μL of fresh CaMHB containing polymyxins, 10% Survanta^®^ (v/v, 2.5 mg/mL phospholipids) was added. MICs in the presence and absence of Survanta^*®*^ were compared to determine the effect of binding to lung surfactant on the antibacterial activity of polymyxins.

### In vitro permeabilization and efflux inhibition assays

Bacterial strains were seeded in 50 mL of Luria-Bertani (LB) broth at OD_600_ 0.05 and grown at 37 °C for 3-4 h until an OD_600_ of 0.6–0.8 was achieved. Bacterial cells were washed twice with 25 mL of buffer (50 mM sodium phosphate, 0.5% glucose, pH 7, with or without 1 mM Mg^2+^), resuspended in 10 mL of the buffer, and then adjusted to an OD_600_ of 0.4. **F365** and polymyxin B (*n* = 3) were titrated in 2-fold dilutions starting with 40 μg/mL in 50 μL of buffer; 50 μL of the substrate (4 × nitrocefin or Leu-Nap) at 256 μg/mL in buffer was added to each concertation followed dilution with 50 μL of the buffer. Bacterial suspension (50 μL, OD_600_ 0.4) in buffer was added to the lipopeptide solutions and the rate of substrate hydrolysis was measured at 490 nm.

### In vitro passaging

Log-phase cultures (OD_600_ = 0.5, ~10^8^ CFU/mL) of *A. baumannii* AB5075 were inoculated (1:10, v/v) in 200 μL fresh CaMHB (cation-adjusted Mueller–Hinton broth) containing 2, 8 and 16 μg/mL polymyxin B, colistin, **F287**, and **F365** in a 96-well microplate with 4 biological replicates. The cultures were incubated at 37 °C for 12 h before the next dilution (1:10, v/v) with fresh CaMHB. Successive subculturing was conducted for 7 days.

### Animal experiments

All animal studies conducted by Monash University and Qpex Biopharma were approved by the Monash Animal Ethics Committee and Qpex Institutional Animal Care and Use Committee, respectively. Mice were housed in micro-isolators in a PC2 animal laboratory with a 12 h/12 h (6 pm, 6 am) dark/light cycle; the room temperature was controlled between 20 and 24 °C and a relative ambient humidity was between 50-70%.

### Acute toxicity in mice

Lipopeptide solutions were prepared in 0.9% saline and stored at 4 °C before use. Swiss mice (female, 7-week-old, 22–28 g) were used in the study. A mouse was administered an intravenous bolus of the lipopeptide (mg/kg free base) through a lateral tail vein (≤0.1 mL). After the injection, the mouse was released back into the cage and monitored for clinical signs of toxicity from 0 to 24 h after injection as follows: every 10 min in the first 2 h, every half hour from 2 to 4 h, every hour from 4 to 12 h, and every 2 h from 12 to 24 h. If the mouse displayed any sign of toxicity, the mouse was humanely sacrificed immediately according to the recommended Euthanasia/Humane Experimental Endpoint Criteria. The maximum dose (mg of free base per kg) that did not cause any side effect was regarded as the MTD (*n* = 4).

### Nephrotoxicity in mice

The experimental methodology has been previously reported by our group^16^. Briefly, individual lipopeptide solutions were prepared in sterile saline (6 mg/mL). Swiss mice (female, 7-week-old, 22–28 g) were subcutaneously administered at 12 mg/kg in, 6 doses every 2 h to reach a total dose of 72 mg/kg (*n* = 3). For high-dose nephrotoxicity experiments, mice were intraperitoneally administered at 20 or 25 mg/kg, in 6 doses every 2 h to reach a total dose of 120 mg/kg (*n* = 6) or 150 mg/kg (*n* = 3). At 20 h after the last dose, the right kidney from each mouse was collected and placed in 10% buffered (pH 7.4) formalin (Sigma-Aldrich, Castle Hill, NSW, Australia). The kidneys were subjected to histological examination at the Australian Phenomics Network-Histopathology and Organ Pathology (University of Melbourne, Parkville, VIC, Australia), who was blind to the treatment groups^16^.

### Neutropenic mouse bloodstream infection model

The experimental methodology for the neutropenic mouse bloodstream infection model was described previously^16^. Briefly, 7-week-old female Swiss mice (22–28 g) were rendered neutropenic by intraperitoneal administration of cyclophosphamide, 4 days (150 mg/kg) and 1 day (100 mg/kg) prior to inoculation. Bloodstream infection was established by intravenous administration of a 50 µL bolus of early log-phase bacterial suspension (4 × 10^8^ CFU/mL). Lipopeptide solutions were prepared at 1 mg/mL (free base) in sterile saline. At 2 h after inoculation, mice (*n* = 3) were injected intravenously with lipopeptide solution at 4 mg/kg (free base) or saline control. At 0 and 4 h after administration, blood was collected, diluted serially in sterile saline, and plated on nutrient agar plates. Agar plates were incubated at 37 °C overnight for bacterial colony counting and the bacterial load in the blood (log_10_ CFU/mL) in each mouse was calculated. In vivo efficacy was calculated as the difference of the log_10_ CFU/mL blood values between the treated mice and the saline-treated mice at 4 h post dosing (Δlog_10_ CFU/mL blood = log_10_ [treated] CFU/mL blood − log_10_[control] CFU/mL blood).

### Neutropenic mouse pneumonia model

The experimental methodology for the neutropenic mouse lung infection model was previously described in detail^13^. Briefly, Swiss mice (female, 7-week-old, 22–28 g) were rendered neutropenic by intraperitoneal administration of cyclophosphamide, 4 days (150 mg/kg) and 1 day (100 mg/kg) prior to inoculation. Lung infection was established by the administration of 25 µL of early log-phase bacterial suspension (polymyxin-susceptible or resistant isolate, 4 × 10^7^ CFU/mL), directly into the lungs via the trachea using a MicroSprayer™ device. Lipopeptide solutions were prepared in sterile saline. At 2 h after inoculation, mice (*n* = 3 or 4) were administered lipopeptide (45–240 mg/kg/d free base) or control (sterile saline) via an intraperitoneal or subcutaneous bolus injection in three divided doses every 8 h. At 0 or 24 h after the administration of lipopeptide solution or saline (control), animals were euthanized and lungs were collected and homogenized under sterile conditions. Homogenate was filtered and the filtrate was collected, serially diluted in sterile saline, and spread on nutrient agar plates. Agar plates were incubated at 37 °C overnight for bacterial colony counting and the bacterial load of the lung (log_10_ CFU/lung) in each mouse was calculated. In vivo efficacy was calculated as the difference of the log_10_ CFU/lung values between the treated mice at 24 h and the pre-treatment (control) at 0 h (Δlog_10_ CFU/lung = log_10_ [treated] CFU/lung − log_10_ [pre-treatment] CFU/lung).

### Neutropenic mouse thigh infection model

The experimental methodology for the neutropenic mouse thigh infection model was previously described in detail^38^. Briefly, Swiss mice (female, 7-week-old, 22–28 g) were rendered neutropenic by intraperitoneal administration of cyclophosphamide 4 days (150 mg/kg) and 1 day (100 mg/kg) prior to inoculation. Thigh infection was established by the administration of a 50 µL bolus of early log-phase bacterial suspension (4 × 10^8^ CFU/mL) directly into each thigh of two mice in each group (*n* = 4 thighs); this method is well accepted in antibiotic pharmacokinetic/ pharmacodynamic studies to minimize animal usage. Lipopeptide solutions were prepared in sterile saline. At 2 h after inoculation, mice were administered a lipopeptide or control solution via an intraperitoneal bolus injection at 15 mg/kg, three doses every 8 h. At 0 and 24 h after the administration, animals were euthanized and each thigh was collected and homogenized under sterile conditions. Homogenate was filtered and the filtrate was collected, serially diluted in sterile saline, and spread on nutrient agar plates. Agar plates were incubated at 37 °C overnight for bacterial colony counting and the bacterial load of both thighs (log_10_ CFU/thigh) in each mouse was calculated. In vivo efficacy was calculated as the difference of the log_10_ CFU/thigh values between the treated mice at 24 h and the pre-treatment (control) at 0 h (Δlog_10_ CFU/thigh = log_10_ [treated] CFU/thigh − log_10_ [pre-treatment] CFU/thigh).

### Pharmacokinetic studies

The plasma pharmacokinetic profiles of **F365** and polymyxin B were assessed in Swiss mice (female, 7-week-old, 22–28 g) after a single intraperitoneal dose (5 mg/kg, *n* = 3). Blood samples were collected at various time points over 24 h post dosing for analysis. The plasma pharmacokinetic profiles of **F365** (*n* = 5) and polymyxin B (1 mg/kg, *n* = 11) in Sprague-Dawley rats (male, 10-week-old, 280 to 330 g) were assessed after intravenous administration (1 min) of **F365** and polymyxin B via an indwelling jugular vein cannula. Blood samples (~0.3 mL) were collected via an indwelling carotid artery cannula at various time points over 24 h post dosing. Urine samples were collected at 4 °C using a metabolic cage during the period between 0 and 24 h post dosing. Concentrations of **F365** and polymyxin B in plasma and urine samples were determined using LC-MS/MS and pharmacokinetic analysis was conducted using WinNonlin (Pharsight Corp., Mountain View, CA). The ELF pharmacokinetic profiles of **F365** and polymyxin B was determined in neutropenic Swiss mice (female, 7-week-old, 22–28 g) after subcutaneous injection (40 mg/kg, *n* = 3). Bronchoalveolar lavage (BAL) samples were collected at various time points over 24 h post dosing from the cannulated trachea using Milli-Q water as BAL washing media. The average volume of ELF in mice was calculated by the ratio of urea concentrations in plasma and BAL samples. Concentrations of **F365** and polymyxin B in plasma and BAL were analyzed by LC-MS/MS. Pharmacokinetic parameters were calculated using a non-compartment model with WinNonlin. For mouse, rat, and human plasma protein binding of **F365** and polymyxin B, pooled plasma samples (*n* = 3) from each species were spiked with **F365** or polymyxin B. The spiked plasma samples were subjected to ultrafiltration and the corresponding filtrates were analyzed by LC-MS/MS to determine the concentration of **F365** and polymyxin B and the protein binding in each species.

### Distribution, accumulation, and metabolism in mouse kidneys

Individual solutions of polymyxin B, colistin, or **F365** were subcutaneously (SC) administered to Swiss mice (female, 7-week-old, 22–28 g) at 10 μmol/kg, 6 doses on Day 1 and 3 doses on Day 2 at every 2 h (*n* = 3). At 2 h after the last dose on Day 2, mice were rapidly euthanized. The left kidney was collected, flash-frozen in liquid nitrogen, and stored at -80˚C for MS imaging analysis. The right kidney was fixed in 10% formalin for histological examination (Histopathology and Organ Pathology Unit at the University of Melbourne). The level of histological damage to the kidneys was graded as previously described^16^. The distribution of lipopeptides in kidney tissues from dosed animals was analyzed by ME-SALDI-MSI on a Shimadzu MALDI-7090, tandem time-of-flight (TOF)-MS instrument in reflectron positive mode at a spatial resolution of 50 µm. Validation of the detected lipopeptides was performed comparing MS/MS analysis results with lipopeptide standards of polymyxin B_1_, polymyxin B_2_, colistin A, colistin B, and **F365**. MS analysis was conducted using 5 mg/mL α-cyano-4-hydroxycinnamic acid (α-CHCA) dissolved in a mixture of 70% acetonitrile and 30% water with 0.1% trifluoroacetic acid (TFA), mixed 1:1 with purified lipopeptide standards. A total of 1 µL was spotted onto electrochemically etched, porous-silicon semiconductor substrates for desorption/ionization on silicon (DIOS)^39^. MS/MS fragmentation experiments were conducted using an ion selection window of ±1.5 Da and helium as the collision gas to generate collision-induced dissociation (CID) spectra. For lipopeptide distribution in mouse kidneys, serially sectioned 8-µm frozen slices of the kidney were loaded onto DIOS substrates and coated with a thin layer of α-CHCA using a matrix sprayer (TM-Sprayer, HTX) for MSI analysis. A thin α-CHCA layer was applied using two spray passes of 5 mg/mL, dissolved as above, at a flow rate of 0.12 mL/min and velocity of 1200 mm/min at 75 °C. Mounted tissue was scanned for imaging alignment (Epson Perfection V370) and calibrated as previously described. Resulting polymyxin maps were visualized and normalized against the total ion current (TIC) of each section using IonView software (Shimadzu). Lipopeptide accumulation in mouse kidneys was determined by simultaneous ME-SALDI-MS analysis of kidney tissue from treated animals and serial dilutions of the drug deposited on control kidney tissue. Two kidney tissue sections (one for the control and one from the treated kidney sample) were placed on the same DIOS substrate for imaging with an adjacent standard curve (12.5–250 µM). The signal intensities were compared to the corresponding concentration standard curve to calculate the concentration of lipopeptide present. Putative metabolites in the kidneys treated with lipopeptides were identified by ME-SALDI-MSI. Control kidney sections were used to differentiate lipopeptide metabolites from endogenous metabolites and MS/MS data from lipopeptide standards were used to differentiate lipopeptide metabolites from the parent ion generated via CID processed in the acquisition software (MALDI Solutions, Shimadzu). Levels of accumulated lipopeptide metabolites were determined by normalization to the parent ion in MSI data.

### Transcriptomic analysis

Mid-log phase *A. baumannii* AB5075 (~10^8^ CFU/mL) in CaMHB was treated with colistin (2 and 8 μg/mL), **F287**, **F319** or **F365** (equimolar concentration to colistin) for 1 and 4 h. Total RNA was extracted using TRIzol Reagent (Invitrogen, USA) and RNeasy Mini Kit (Qiagen, Germany). Quality check of RNA samples was conducted with NanoDrop (Thermo Fisher Scientific, USA) and Agilent 2100 Bioanalyzer (Agilent Technologies, USA) for sequencing (150 bp paired-end) at Genewiz (Shanghai, China). Raw reads were aligned to *A. baumannii* AB5075 genome (GCF_000963815, https://www.ncbi.nlm.nih.gov/assembly/GCF_000963815.1/) using subread-aligner (subread 2.0.1) and summarized by featureCounts (subread 2.0.1). Principal component analysis (PCA) was conducted with mixOmics 6.14.1 in R. Differentially expressed genes were determined using limma 3.46.0 with a combination of fold change ≥ 2 and false discovery rate (FDR, by Benjamini–Hochberg algorithm) adjusted *P*-value ≤ 0.05 (*n* = 3). Enrichment analysis of gene differential expression in AB5075 was conducted using BioCyc (*A. baumannii* AB5075, version 25.5). UpSetR 1.4.0, ComplexHeatmap 2.6.2 and circlize 0.4.13 were used for data visualization. Raw data were deposited in Short Reads Archive (SRA) database under accession numbers SRR15235669-SRR15235725.

Human Kidney-2 (HK-2) cells (CRL-2190^™^, American Type Culture Collection) were treated with 100 μM colistin, **F287**, **F319**, and **F365** for 24 h. Previous transcriptomic data of HK-2 cells following 24 h treatment with polymyxin B^26^ were also employed for comparative bioinformatic analysis. RNA extraction was conducted as described above. Raw reads were aligned to genome GRCh38.94 (Ensembl genome database, http://ftp.ensembl.org/pub/release-94/fasta/homo_sapiens/dna/) using subjunct and summarized by featureCounts. Differentially expressed genes were determined using limma 3.46.0, with a combination of fold change ≥ 1.5 and FDR (by Benjamini–Hochberg algorithm) adjusted *P*-value ≤ 0.05 (*n* = 3). Key regulatory genes of HK-2 were analyzed in the context of signaling network in the Signor 2.0 database. Enrichment analysis of gene differential expression in HK-2 cells was conducted using enrichR 2.1. UpSetR 1.4.0, ComplexHeatmap 2.6.2, circlize 0.4.13 and iGraph 1.2.11 were used for data visualization. Raw data were deposited in the Short Reads Archive database under accession numbers SRR15239061-SRR15239078.

### Toxicology in monkeys

A 14-day repeat-dose GLP toxicology study of **F365** was conducted in Cynomolgus monkeys. The study complied with the OECD Principles of Good Laboratory Practice. Non-GMP grade **F365** HCl salt (95.8% purity) manufactured by PolyPeptide (San Diego, USA) was used as the test material for the study. Cynomolgus monkeys were approximately 2–4 years of age and weighed between 2.0 and 2.9 kg at the start of treatment. Four male and four female cynomolgus monkeys were used for each dose group. **F365** (in 0.9% sodium chloride for injection) was administered four times daily (i.e. 6 h ± 15 min apart) as 60-min intravenous infusion at 5, 20, and 50 mg/kg/day for 14 consecutive days; the reference item (vehicle control) was also administered. Assessments of mortality checks, clinical observations, body weight, ophthalmology, and electrocardiography were conducted. Blood and urine samples were collected pre-treatment and at the end of the dosing period for hematology, clinical chemistry, coagulation, and urinalysis assessments. Other blood samples were collected after the first and last dose for toxicokinetic assessment. Following the end of the 14-day dosing period, animals were euthanized on Day 16 and examined macroscopically. Organ weights and macroscopic observations were recorded at necropsy for all groups. Histopathological examinations were performed on the specified tissues and organ list for animals across all groups.

### Statistics

All statistical analyses including two-detailed Student’s *t*-test and one-way analysis of variance (ANOVA) were performed in R. Differentially expressed genes were identified using limma package^40^. For ANOVA, Tukey’s honestly significant difference (HSD) *post-hoc* tests were conducted for pairwise comparisons. Benjamini–Hochberg procedure was used to FDR adjust *P*-values in multiple tests. Unless otherwise stated, all data are shown as mean ± standard deviation (s.d.).

### Reporting summary

Further information on research design is available in the Nature Research Reporting Summary linked to this article.

## Supplementary information

Supplementary Information

Reporting Summary

## Data Availability

Data supporting the findings of this study are available within the article and the supplementary information or from the corresponding authors on reasonable request. Transcriptomics were deposited in the Short Reads Archive database under accession numbers SRR15235669-SRR15235725 and SRR15239061-SRR15239078. Source data are provided with this paper.

## Source data

Source Data

## Code availability

All codes for RNA-Seq data processing and analysis are available at 10.5281/zenodo.5915369^41^.
