# Supplementary material for: A synthetic lipopeptide targeting top-priority multidrug-resistant Gram-negative pathogens
Source: Nat Commun. 2022 Mar 25;13:1625. doi: 10.1038/s41467-022-29234-3 (PMC8956739; doi:10.1038/s41467-022-29234-3)
Supplement: Supplementary file 1 — Supplementary Information [file 41467_2022_29234_MOESM1_ESM.pdf]

Supplementary Information

A synthetic lipopeptide targeting top-priority multidrug-resistant Gram-negative pathogens

Kade D. Roberts, Yan Zhu, Mohammad A. K. Azad, Mei-Ling Han, Jiping Wang, Lynn Wang, Heidi H. Yu, Andrew S. Horne, Jo-Anne Pinson, David Rudd, Nicolas H. Voelcker, Nitin A. Patil, Jinxin Zhao, Xukai Jiang, Jing Lu, Ke Chen, Olga Lomovskaya, Scott J. Hecker, Philip E. Thompson, Roger L. Nation, Michael N. Dudley, David C. Griffith, Tony Velkov, Jian Li

Correspondence should be addressed to J.L. (email: jian.li@monash.edu) or to T.V. (email: tony.velkov@unimelb.edu.au).

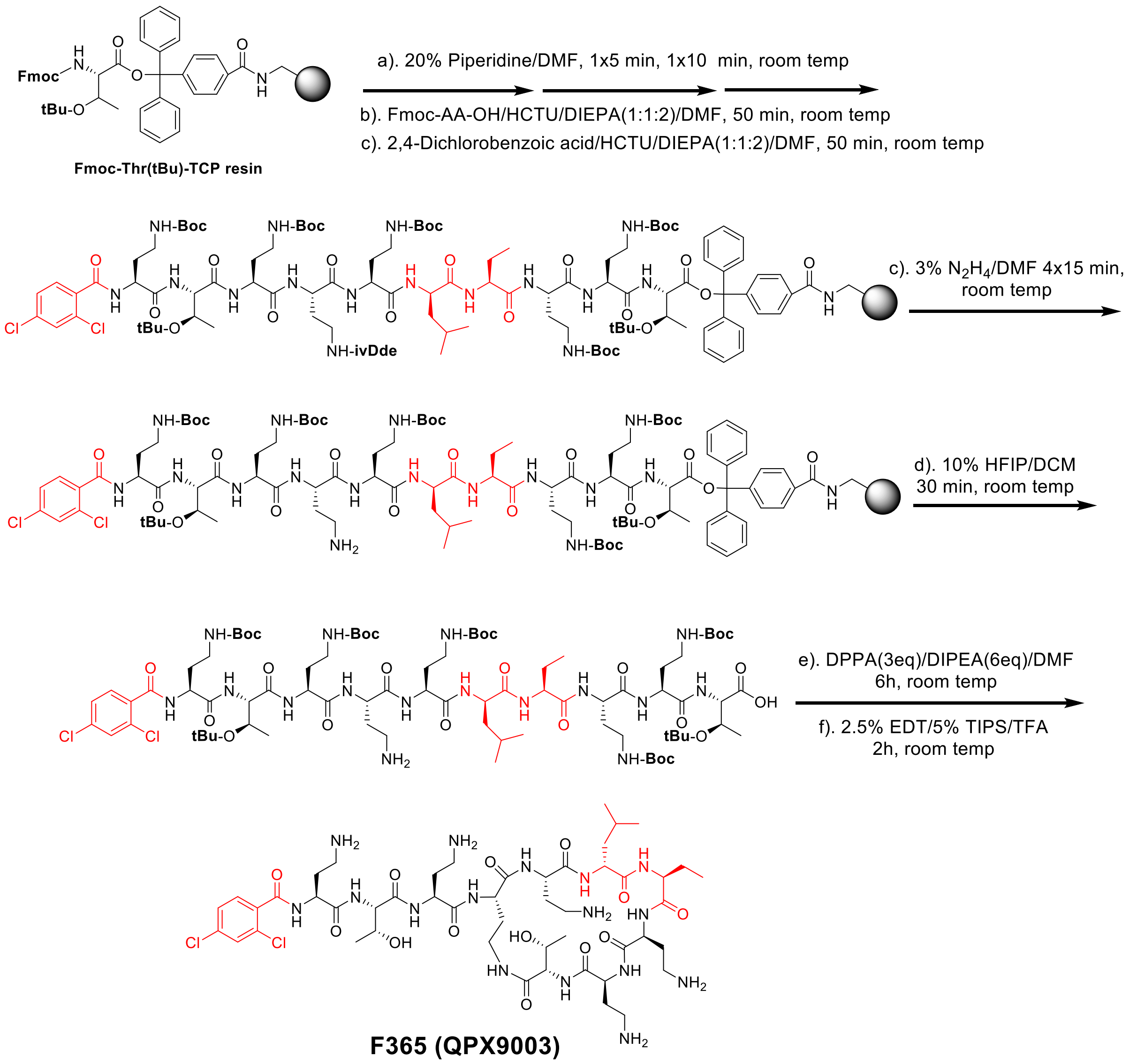

Supplementary Fig. 1 Lipopeptide synthesis platform highlighting the synthesis of **F365** (**QPX9003**).

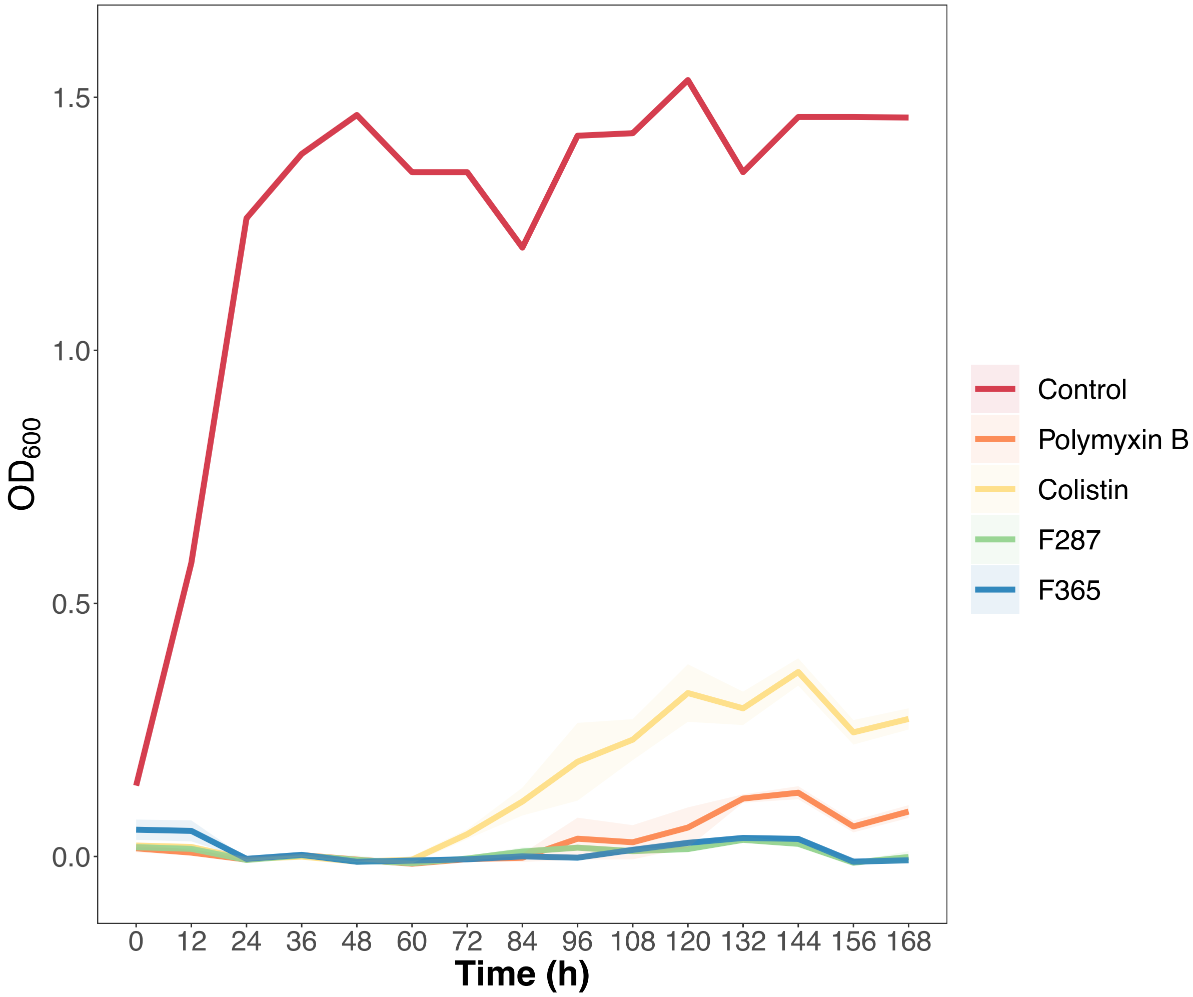

Supplementary Fig. 2 Emergence of resistance during *in vitro* passaging of *A. baumannii* AB5075 every 12 h in CaMHB with 16× MIC (i.e. 8 μg/mL) polymyxin B, colistin, **F287**, and **F365** (n = 4; control group, n = 1). Data are shown as mean ± s.d. (solid line and shading, respectively). MICs of **F365**, **F287**, polymyxin B and colistin are 0.5 μg/mL.

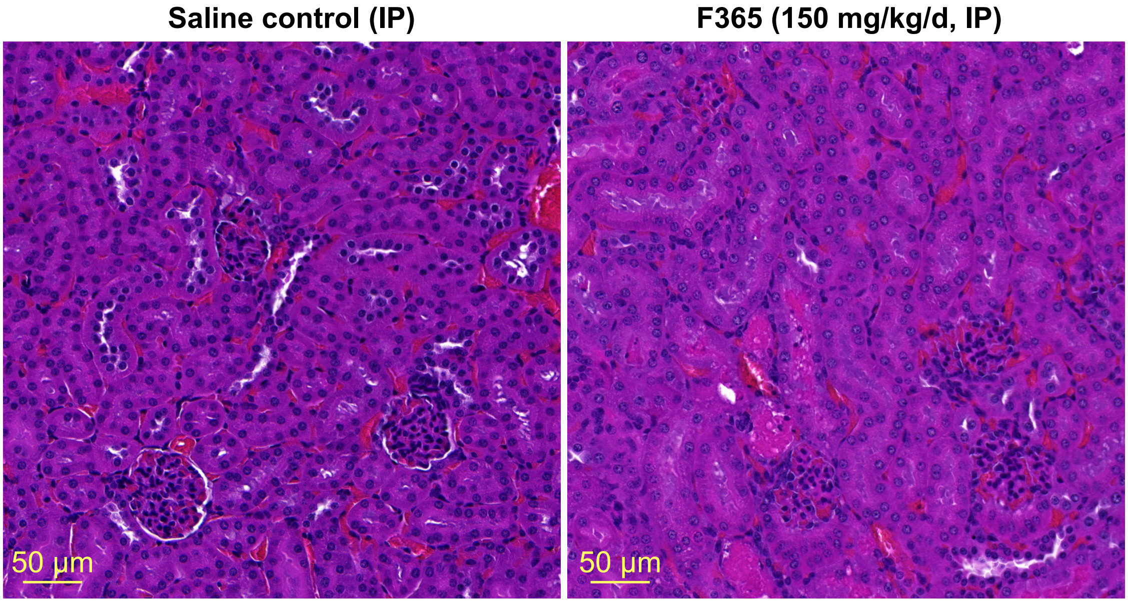

Supplementary Fig. 3 High-dose nephrotoxicity in mice. Representative histology images of mouse kidney tissue after dosing with the saline control (n = 4) or 150 mg/kg/d **F365** (*n* = 3 mice) intraperitoneally (in 6 divided doses every 2 h) showing comparable histology. All mice (3/3) in the **F365** treatment group had an SQS score = 0, i.e. no significant damage to the kidneys.

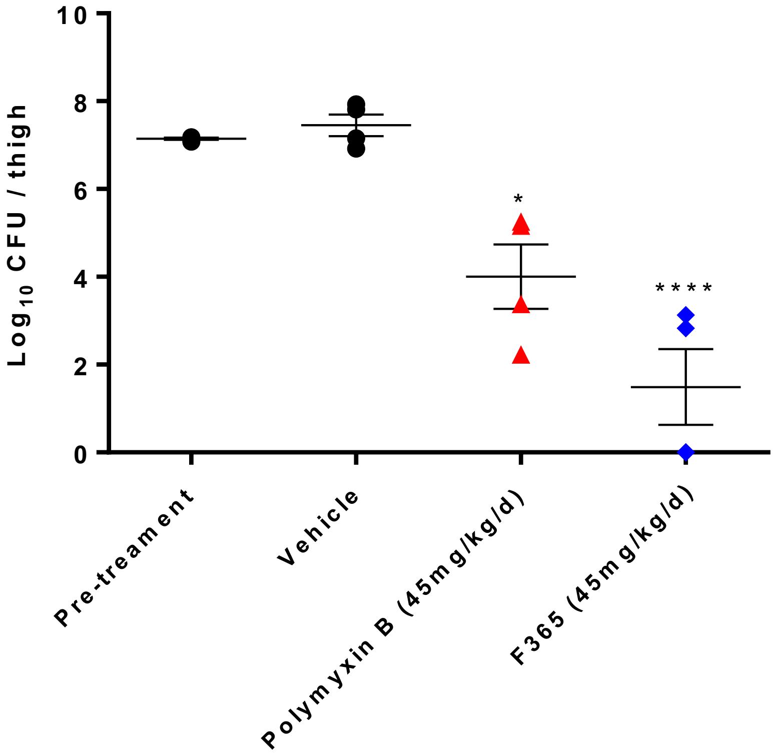

Supplementary Fig. 4. Efficacy of **F365** and polymyxin B in a neutropenic mouse thigh infection model against a polymyxin-susceptible MDR clinical isolate *A. baumannii* FADDI-AB30 (carbapenem-resistant, **F365** and polymyxin B MIC = 0.25 µg/mL). **F365**, polymyxin B or the vehicle was administered (in 3 divided doses) intraperitoneally every 8 h over 24 h. Each data point is from an independently infected thigh in a neutropenic mouse and two mice were infected for each treatment to minimize the use of animals. Two-sided one-way ANOVA (F=23.79, P=2.44e-05, DF=3) was conducted to compare multiple groups, followed by Tukey's HSD (honestly significant difference) tests for pairwise comparisons (with FDR adjustment for multiple comparisons, P-values are provided in Source Data). Data are represented as mean ± s.e.m. (n = 4 thighs). FDR-adjusted P-values * $P < 0.05$, **** $P < 0.0001$ relative to the pre-treatment group.

Activity of F365 and polymyxin B in the outer membrane permeabilization assay.

The outer and inner membranes of gram-negative bacteria are the sites of interaction and action of polymyxin antibiotics, respectively. These polycationic compounds bind to phosphate residues of lipid A and displace the magnesium and calcium ions that cross-bridge adjacent lipid A molecules thereby stabilizing the outer membrane. When these divalent cations are displaced with the bulkier polycationic polymyxin, the membrane becomes weakened, and the permeability barrier is disrupted allowing for uptake of previously nonpermeating or weakly permeating molecules as well as leakage of periplasmic proteins. The displacement of divalent cations and permeabilization allows for self-promoted uptake of the polymyxin molecule itself enabling it to penetrate the periplasm and approach the cytoplasmic membrane. Disruption and/or permeabilization of Gram-negative bacterial cytoplasmic membrane has been suggested as the main mechanism of action by polymyxin B and colistin. The outer membrane-permeabilizing effect of **F365** at a wide range of concentrations was assessed by examining the rates of hydrolysis of a chromogenic β-lactam, nitrocefin, by intact cells of *Pseudomonas aeruginosa*. For these experiments we used the strain of *P. aeruginosa* PAM2035 that constitutively produces the β-lactamase AmpC, encoded by the corresponding gene normally present in the genome of this bacterium. An increased rate of hydrolysis in intact cells is indicative of increased permeation of nitrocefin to the periplasmic β-lactamase since the rate of hydrolysis is limited by the rate of diffusion across the outer membrane. Polymyxin B was used as a positive control. Both **F365** and polymyxin B demonstrated the ability to permeabilize the outer membrane of *P. aeruginosa* (Supplementary Fig. 5a). IC_{50} values for membrane permeabilization were 0.73 ± 0.07 and 1.15 ± 0.22 μg/mL for **F365** and polymyxin B, respectively. As expected, the permeabilizing effects of both polymyxin B and **F365** were significantly decreased by the addition of 1 mM Mg^{2+} (Supplementary Fig. 5a).

Activity of F365 and polymyxin B in the efflux inhibition assay.

One of the consequences of disruption/permeabilization of the cytoplasmic membrane by polymyxins is the dissipation of the proton gradient across the cytoplasmic membrane. Hence, these compounds are expected to inhibit proton-dependent transport activity across the cytoplasmic membrane including efflux. Efflux activity and its inhibition by **F365** was assessed by examining the rates of hydrolysis of a fluorogenic substrate of efflux pumps, Leu-Nap, by intact cells of *P. aeruginosa*. For these experiments we used two strains of *P. aeruginosa*, PAM1723 and PAM1626. PAM1723 overexpresses the efflux pump MexAB-OprM and PAM1626 lacks this pump as well as several other pumps that extrude Leu-Nap. Leu-Nap, which is not fluorescent in solution, is cleaved enzymatically inside the cells to produce the highly fluorescent compound β-naphthylamine. The rate of production of β-naphthylamine (recorded as an increase in fluorescence) is limited by the rate of entry of Leu-Nap into the cells. The rate of cleavage of Leu-Nap was much higher in PAM1626 than in PAM1723, indicating efflux of Leu-Nap by the MexAB-OprM efflux pump produced in by PAM1723. Both **F365** and Polymyxin B increased the rate of Leu-Nap hydrolysis by *P. aeruginosa* PAM1723 by inhibiting Leu-Nap efflux and had no effect on the rate of Leu-Nap hydrolysis in the efflux-deficient strain PAM1626 (Supplementary Fig. 5b). The effect of **F365** on the inhibition of efflux activity was approximately two-fold higher than that of polymyxin B, with IC_{50} values of 4.2 ± 1.5 and 7.6 ± 1.4 µg/mL, respectively (Supplementary Fig. 5b).

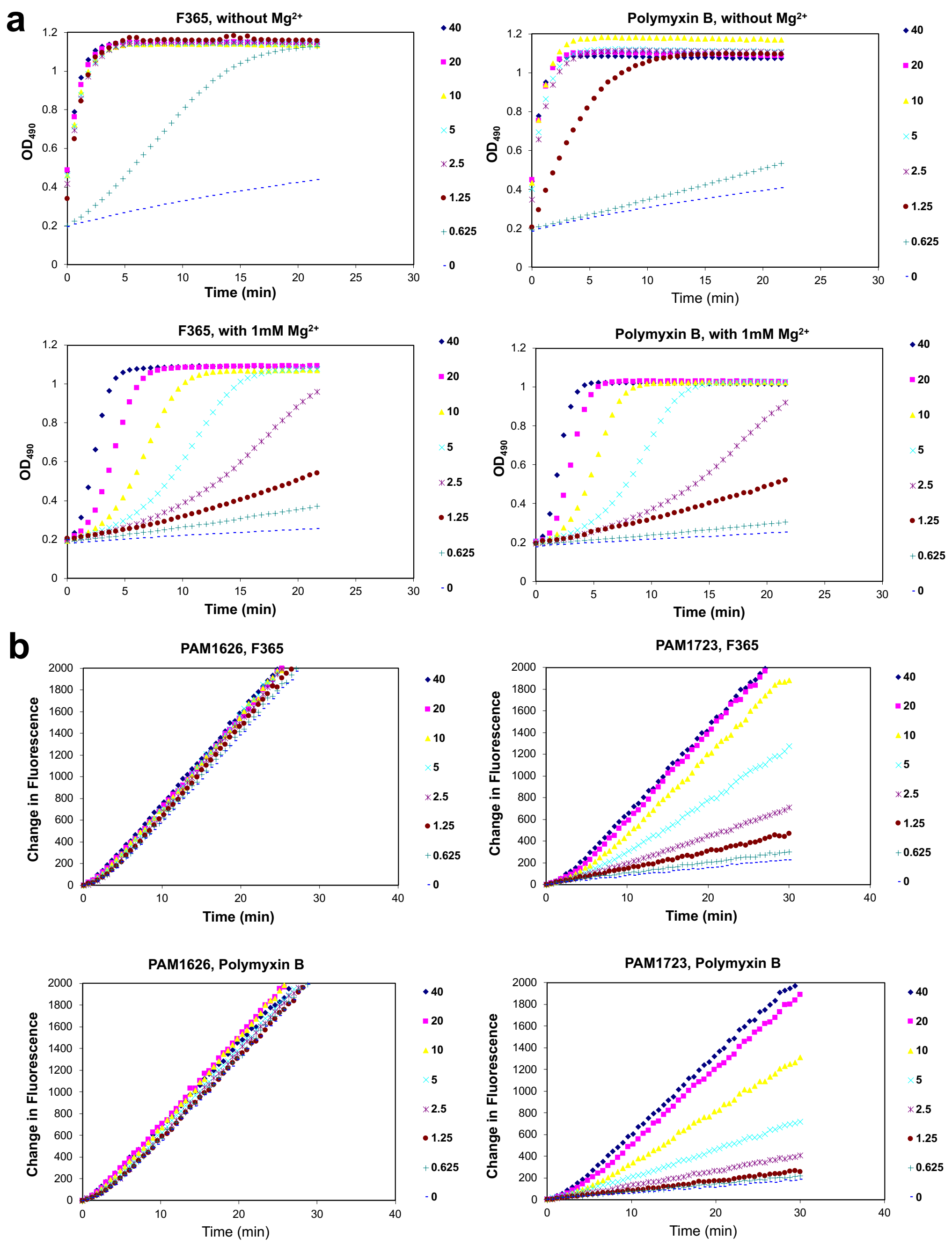

Supplementary Fig. 5 Outer membrane permeabilization and efflux inhibition activity of **F365** and polymyxin B. Data are shown as mean of three replicates and the unit in the legend is μg/mL. **a** Activity (μg/mL) of **F365** and polymyxin B in an outer membrane permeabilization assay. Assessment of the rate of hydrolysis of a chromogenic β-lactam, nitrocefin, by periplasmic β-lactamase in intact cells of *P. aeruginosa* PAM2035 in the presence and absence of 1 mM Mg^{2+}. **b** Activity (μg/mL) of **F365** and polymyxin B (n = 3) in an efflux inhibition assay. Examination of the rates of intracellular hydrolysis of a fluorogenic substrate Leu-Nap after uptake by efflux pumps. *P. aeruginosa* PAM1723 overexpresses the efflux pump MexAB-OprM, while *P. aeruginosa* PAM1626 lacks the efflux pump as well as several other pumps that are capable to promote the uptake of Leu-Nap.

a

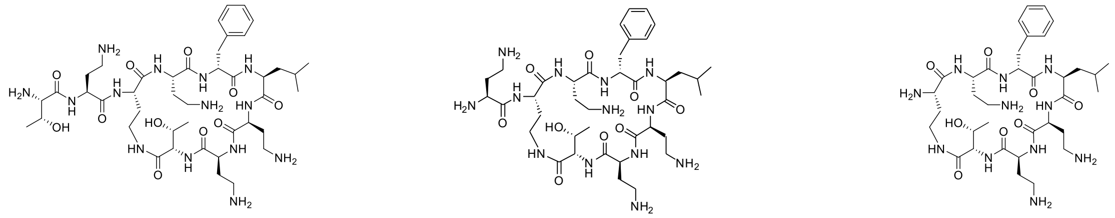

Polymyxin B metabolite - M1 **Polymyxin B metabolite - M2** **Polymyxin B metabolite - M3**

Ion signals - Polymyxin B_1			
**Metabolites**	**[M+H]⁺**	**[M+Na]⁺**	**[M+K]⁺**
M1	963.5734	985.5734	**1001.6734**
M2	862.5257	**884.5257**	**900.6257**
M3	762.4621	**784.4621**	**800.5621**

Ion signals - Polymyxin B_2			
**Metabolites**	**[M+H]⁺**	**[M+Na]⁺**	**[M+K]⁺**
M1	963.5734	985.5734	**1001.6734**
M2	862.5257	884.5257	**900.6257**
M3	762.4621	784.4621	**800.5621**

b

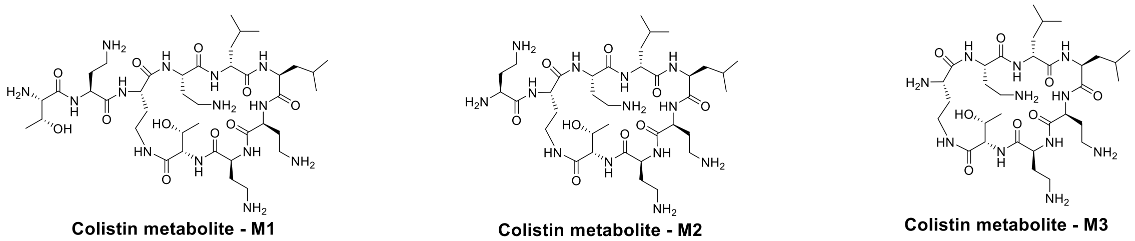

Colistin metabolite - M1 **Colistin metabolite - M2** **Colistin metabolite - M3**

Ion signals - Colistin A			
**Metabolites**	**[M+H]⁺**	**[M+Na]⁺**	**[M+K]⁺**
M1	929.5891	951.5891	**967.6891**
M2	828.5414	850.5414	**866.6414**
M3	728.4778	**750.4778**	**766.5778**

Ion signals - Colistin B			
**Metabolites**	**[M+H]⁺**	**[M+Na]⁺**	**[M+K]⁺**
M1	929.5891	951.5891	**967.6891**
M2	828.5414	850.5414	**866.6414**
M3	728.4778	750.4778	**766.5778**

c

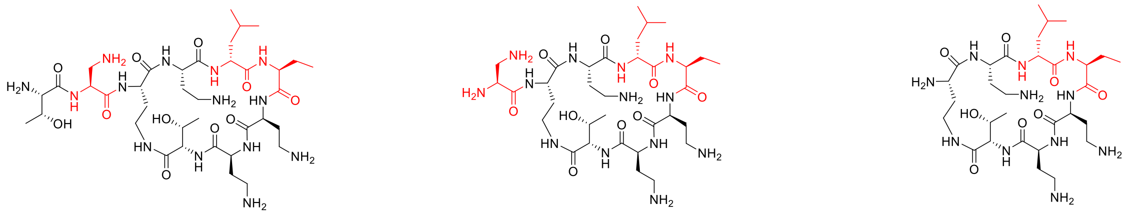

F365 metabolite - M1 **F365 metabolite - M2** **F365 metabolite - M3**

Ion signals - F365			
**Metabolites**	**[M+H]⁺**	**[M+Na]⁺**	**[M+K]⁺**
M1	887.5422	909.5422	925.6422
M2	786.4945	808.4945	**824.5945**
M3	700.4465	**722.4465**	**738.5465**

Supplementary Fig. 6 Metabolism of polymyxin B and colistin and **F365** in mouse kidneys. Mice were administered 10 µmol/kg subcutaneously, 6 doses (2-h interval) on Day 1 and 3 doses (2-h interval) on Day 2 (n = 3). **a** Structures of putative metabolites (**M1**, **M2**, **M3**) of polymyxin B_1 and B_2 and their corresponding $[M+H]^+$, $[M+Na]^+$ and $[M+K]^+$ molecular ions. Actual molecular ions observed are highlighted in red. **b** Structures of potential metabolites (**M1**, **M2**, **M3**) of colistin A and B and their corresponding $[M+H]^+$, $[M+Na]^+$ and $[M+K]^+$ molecular ions. Actual molecular ions observed are highlighted in red. **c** Structures of potential metabolites (**M1**, **M2**, **M3**) of **F365** and their corresponding $[M+H]^+$, $[M+Na]^+$ and $[M+K]^+$ molecular ions. Actual molecular ions observed are highlighted in red.

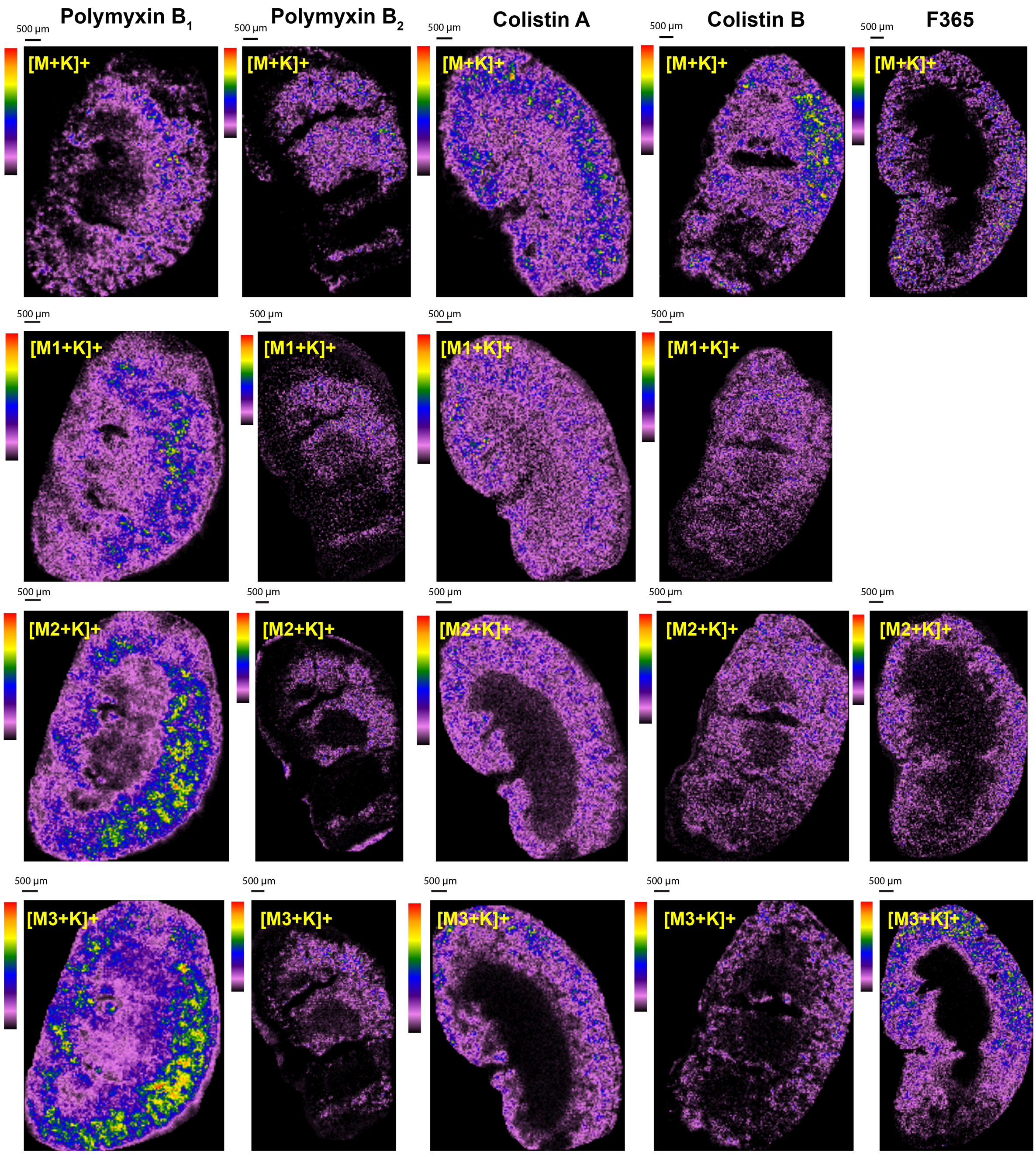

Supplementary Fig. 7 Representative ME-SALDI-MS images highlighting the distribution and accumulation of the metabolites (**M1**- **M3**) for polymyxin B_1, polymyxin B_2, colistin A, colistin B and **F365** in mouse kidney tissue. Mice were administered 10 µmol/kg subcutaneously, 6 doses on Day 1 (2-h interval) and 3 doses on Day 2 (2-h interval) (n = 3). No ion signals corresponding to the **M1** metabolite for **F365** were observed. All ME-SALDI-MS images were normalized to total ion count based on the highest intensity peak across each tissue section and its corresponding concentration curve. Selected lipopeptide related ions are displayed with a scale between 0 and 40% relative to the highest intensity peak in the summed spectrum of the whole tissue. A default colour intensity gradient is used to visualize and differentiate low concentrations (purple to blue), mid-range concentrations (green to yellow) and high concentrations (orange to red) of lipopeptide disposition.

Supplementary Table 1 Structures of native polymyxins, highlighting the non-conserved regions at the *N*-terminus (R_1), position 3 (R_3), position 6 (R_6), and position 7 (R_7).

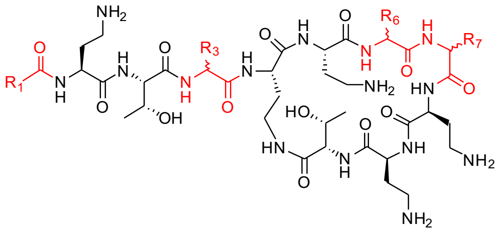

Polymyxin	(R_1)	(R_3)	(R_6)	(R_7)
**B1**	(*S*)-6-Methyloctanoyl	Dab	D-Phe	Leu
**B2**	6-Methylheptanoyl	Dab	D-Phe	Leu
**B1 (Ile)**	(*S*)-6-Methyloctanoyl	Dab	D-Phe	**Ile**
**B1 (Dab^3)**	(*S*)-6-Methyloctanoyl	**D-Dab**	D-Phe	Leu
**B2 (Ile)**	6-Methylheptanoyl	Dab	D-Phe	**Ile**
**B2 (Dab^3)**	6-Methylheptanoyl	**D-Dab**	D-Phe	Leu
**B3**	**Octanoyl**	Dab	D-Phe	Leu
**B4**	**Heptanoyl**	Dab	D-Phe	Leu
**B5**	**Nonanoyl**	Dab	D-Phe	Leu
**B6**	**3-Hydroxy-6-methyloctanoyl**	Dab	D-Phe	Leu
**A1**	(*S*)-6-Methyloctanoyl	**D-Dab**	**D-Leu**	**Thr**
**A2**	6-Methylheptanoyl	**D-Dab**	**D-Leu**	**Thr**
**D1**	(*S*)-6-Methyloctanoyl	**D-Ser**	**D-Leu**	**Thr**
**D2**	6-Methylheptanoyl	**D-Ser**	**D-Leu**	**Thr**
**Colistin A (E1)**	(*S*)-6-Methyloctanoyl	Dab	**D-Leu**	Leu
**Colistin B (E2)**	6-Methylheptanoyl	Dab	**D-Leu**	Leu
**E3**	**Octanoyl**	Dab	**D-Leu**	Leu
**E4**	**Heptanoyl**	Dab	**D-Leu**	Leu
**E7**	**7-Methyloctanoyl**	Dab	**D-Leu**	Leu
**E1 (Ile)**	(*S*)-6-Methyloctanoyl	Dab	**D-Leu**	**Ile**
**E1 (Val)**	(*S*)-6-Methyloctanoyl	Dab	**D-Leu**	**Val**
**E1 (Nva)**	(*S*)-6-Methyloctanoyl	Dab	**D-Leu**	**Nva**
**E2 (Ile)**	6-Methylheptanoyl	Dab	**D-Leu**	**Ile**
**E2 (Val)**	6-Methylheptanoyl	Dab	**D-Leu**	**Val**
**E8 (Ile)**	**7-Methylnonanoyl**	Dab	**D-Leu**	**Ile**
**M1**	(*S*)-6-Methyloctanoyl	Dab	**D-Leu**	**Thr**
**M2**	6-Methylheptanoyl	Dab	**D-Leu**	**Thr**
**P1**	(*S*)-6-Methyloctanoyl	**D-Dab**	D-Phe	**Thr**
**P2**	6-Methylheptanoyl	**D-Dab**	D-Phe	**Thr**
**S1**	(*S*)-6-Methyloctanoyl	**D-Ser**	D-Phe	**Thr**

Supplementary Table 2 Nephrotoxicity of F287, F365 and polymyxin B in mice.

**Lipopeptide**	***n***	**Total dose* (mg/kg/d)**	**Kidney histological damage**
**Saline control**	6	0	No damage; 6/6 mice
**Polymyxin B****	6	60	Moderate to severe; 6/6 mice
**F287**	6	120	Minimal; 2/6 mice
**F365**	6	120	No damage; 6/6 mice

* Administered IP in 6 divided doses every 2 h. ** 60 mg/kg/d was the highest dose of polymyxin B that could be safely administered intraperitoneally.

Supplementary Table 3 MICs (μg/mL) of polymyxin B and F365 against polymyxin-resistant clinical isolates.

Polymyxin-resistant isolate*	MIC (μg/mL)	
	Polymyxin B	F365
P. aeruginosa		
FADDI-PA070	>32	>32
FADDI-PA090	4	8
FADDI-PA102**	4	4
A. baumannii		
FADDI-AB144	16	32
FADDI-AB156**	8	4
FADDI-AB161	8	1
K. pneumoniae		
Kp MKP103	16	8
FADDI-KP132***	8	8
FADDI-KP027	>32	>32

* EUCAST breakpoints of colistin were employed: Susceptible ≤ 2 μg/mL, Resistant > 2 μg/mL.

** carbapenem-resistant

*** mobilized colistin resistance 1 (*mcr-1*) positive

Supplementary Table 4 Pharmacokinetics of F365 and polymyxin B in rodents.

Lipopeptide	Dose (mg/kg)	Route of dosing	Protein binding (%)	Total CL (L/h/kg)	$AUC_{(0-\infty)}$ (mg·h/L)	Free $AUC_{(0-\infty)}$ (mg·h/L)	Half-life (h)	Urinary recovery (%)
**Mouse (Plasma)**								
**F365**	5	IP	36	0.63	7.92	5.07	0.30	n.d.
PMB	5	IP	95	0.14	31.3	1.56	1.40	n.d.
**Mouse (ELF)**								
**F365**	40	SC	-	0.46	86.2	86.2*	3.39	n.d.
PMB	40	SC	-	0.50	80.0	10.0*	6.70	n.d.
**Rat (Plasma)**								
**F365**	1	IV	33	0.46 ± 0.15	2.40 ± 0.85	1.61 ± 0.57	0.91 ± 0.21	2.41% ± 1.73%
PMB	1	IV	80	0.20 ± 0.09	5.73 ± 1.98	1.15 ± 0.40	1.45 ± 0.50	0.54% ± 0.25%

PMB = polymyxin B

Mouse (plasma) n = 3; mouse (ELF) n = 4; rat (plasma) n = 5 for **F365**, n = 11 for polymyxin B.

* Estimated $fAUC_{ELF}$ based on the change in the MICs for **F365** (no change) and polymyxin B (8-fold increase) in the presence of 10% Survanta® (Fig. 2b).

n.d. = not determined.

Supplementary Table 5 Plasma AUC_{0-24} and microscopic observations for the kidneys in a 14-day GLP monkey toxicology study.

Lipopeptide	*n*	Dose (mg/kg)	Dosing Interval	Total Daily Dose (mg/kg)	AUC_{0-24} (mg·h/L) Day 1	AUC_{0-24} (mg·h/L) Day 14	Histopathology
**F365**	8	1.25	q6h	5	24.0	26.4	8/8 no changes
**F365**	8	5	q6h	20	97.2	110.4	6/8 no changes<br>2/8 minimal tubular degeneration
**F365**	8	12.5	q6h	50	246.4	377.6	2/8 no changes<br>4/8 minimal tubular degeneration<br>1/8 mild tubular degeneration<br>1/8 moderate tubular degeneration
**Control***	8	0	q6h	0	-	-	8/8 no changes

* Received the reference item 0.9% sodium chloride for injection (USP).

Materials, methods and analytical data for lipopeptide synthesis

Materials. Polymyxin B sulfate and colistin sulfate were obtained from BetaPharm (Shanghai, China). The isolation of purified polymyxin B_1 and polymyxin B_2, colistin A and B from commercial preparations of polymyxin B and colistin, respectively, was conducted as previously described (*16*). Piperidine, diisopropylethylamine (DIPEA) and trifluoroacetic acid (TFA) were purchased from Auspep (Melbourne, Australia). Fmoc-Dab(Boc)-OH, was purchased from Try-lead Chem (Hangzhou, China). Fmoc-D-Phe-OH, Fmoc-Dab(ivDde)-OH, 1,1,1,3,3,3-Hexafluoro -2-propanol (HFIP) and 1H-Benzotriazolium-1-[bis(dimethylamino) methylene]-5-chlorohexafluorohosphate-(1-),3-oxide (HCTU) were purchased from Chem-Impex International (Wood Dale, IL, USA). Fmoc-Ala-OH, Fmoc-Leu-OH and Fmoc-Thr(tBu)-OH were purchased from Mimotopes (Melbourne, Australia). Dimethylformamide (DMF), methanol (MeOH), diethyl ether, dichloromethane (DCM), hydrochloric acid (HCl) and acetonitrile were purchased from Merck (Melbourne, Australia). Fmoc-Thr(tBu)-TCP-Resin was purchased from Intavis Bioanalytical Instruments (Germany). Triisopropylsilane (TIPS), 1,2-Ethanedithiol (EDT), diphenylphosphorylazide (DPPA) and hydrazine were obtained from Sigma-Aldrich (Castle Hill, Australia).

Synthesis protocol. Synthesis of the protected linear lipopeptide was conducted on a Protein Technologies Prelude automated peptide synthesizer using standard Fmoc solid-phase peptide chemistry. Synthesis was carried out using TCP-Resin, pre-loaded with Fmoc-Thr(tBu)-OH (0.1 mmol scale). Coupling of all Fmoc-amino acids was performed using the same standard protocol with 3 molar equivalents (relative to resin loading) of the Fmoc amino acid and the coupling reagent HCTU in DMF with activation *in situ*, using 6 molar equivalents of DIPEA. The reaction was left to proceed for 50 min at room temperature, followed by washing of the resin with DMF. Fmoc deprotection was conducted using the protocol 20% piperidine in DMF (1 × 5 min, 1 × 10 min), followed by washing of the resin with DMF at room temperature. Removal of the ivDde protecting group was achieved with 3% hydrazine in DMF (4 × 15 min), followed by washing of the resin with DMF. The protected linear lipopeptide was then cleaved from the resin by treating the resin with 10% hexafluoroisopropanol (HFIP) in DCM (1 × 30 min, 1 × 5 min). This solution was concentrated *in vacuo* to give the crude protected linear lipopeptide. The protected linear lipopeptide was dissolved in DMF (5 mL) to which DIPEA 0.6 mmol, 104 µL and DPPA, 0.3 mmol, 0.65 µL were added, the resulting solution was then stirred for 6 h at room temperature. The cyclisation solution was concentrated under vacuum overnight and the resulting residue taken up in a solution of 2.5% EDT, 5% TIPS in TFA and stirred for 2 h at room temperature. To this solution 40 mL of diethyl ether was added to precipitate the crude the lipopeptide resulting precipitate was collected by centrifugation and washed twice with diethyl ether (40 mL) then dried to give the crude cyclic lipopeptide product as a white solid. The crude cyclic lipopeptide was dissolved in Milli-Q water (5 mL) and de-salted using a Vari-Pure IPE SAX column then purified by preparative RP-HPLC. Fractions collected were analysed by LC-MS. Fractions with the desired purity were combined and lyophilised for two days to give the lipopeptide product as its corresponding TFA salt. The yields obtained for each lipopeptide, their corresponding analytical data and full chemical

structures are shown below. Modifications made to the lipopeptide scaffold are highlighted in red. Preparative RP-HPLC was carried on a Waters Prep LC system with a Waters 486 tuneable absorbance detector (214 nm) and a Phenomenex Axia Luna C8(2) column (250 × 21.2 mm i.d., 100 Å, 10 μm). Lipopeptides were eluted from the column with a gradient of 0−60% buffer B over 60 min at a flow rate of 15 mL/min; buffer A was 0.1% TFA/water, and buffer B was 0.1% TFA/acetonitrile. LC-MS analysis was conducted on a Shimadzu 2020 LC-MS system, incorporating a photodiode array detector (214 nm) coupled to an electrospray ionization source and a single quadrupole mass analyser. A Phenomenex Luna C8(2) column (100 × 2.0 mm i.d., 100 Å, 3 μm) was used, eluting with a gradient of 0-60% solvent B over 10 min at a flow rate of 0.2 mL/min. Solvent A was 0.05% TFA in water and Solvent B 0.05% TFA in acetonitrile. Mass spectra were acquired in the positive ion mode with a scan range of 200 - 2,000 *m/z*. ^{1}H NMR spectra were collected at 25 °C on 600 MHz on a Bruker Avance III 600 spectrometer. The lipopeptide solutions were prepared in D_2O at 1 mg/100 μL. Chemical shifts (δ) are reported in ppm downfield from TMS. Coupling constants J are recorded in Hertz (Hz). High-resolution mass spectral (HRMS) analysis of **F365** was carried out on a Thermo Fisher Q-Exactive Orbitrap *via* direct infusion of the lipopeptide (dissolved in MeOH-H_2O 1:1 at 25 μg/mL) in positive mode with a mass range of 150-2,000 *m/z* and a resolution of 280,000.

F085:

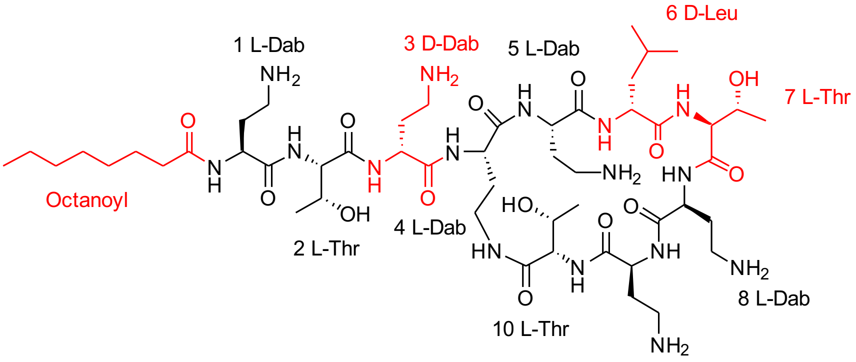

TFA salt was obtained in a yield of 49.5 mg, retention time (t_R) at 214 nm = 11.583 min (HPLC purity: 98.5%). ESI-MS analysis of peak at 11.583 min: *m/z* (monoisotopic) $[M+H]^+$ 1144.2, $[M+2H]^{2+}$ 572.7 $[M+3H]^{3+}$ 382.3. Calculated *m/z* (monoisotopic) for **F085** ($C_{50}H_{94}N_{16}O_{14}$) $[M+H]^+$ 1143.7, $[M+2H]^{2+}$ 572.4, $[M+3H]^{3+}$ 381.9. ^{1}H NMR (600 MHz, D_2O) δ 4.49 – 4.40 (m, 3H), 4.40 – 4.09 (m, 10H), 3.61 – 3.55 (m, 1H), 3.35 – 3.27 (m, 1H), 3.17 – 2.92 (m, 11H), 2.32 – 1.77 (m, 15H), 1.68 – 1.46 (m, 6H), 1.25 – 1.11 (m, 18H), 0.91 – 0.83 (m, 6H), 0.80 – 0.76 (m, 3H).

F100:

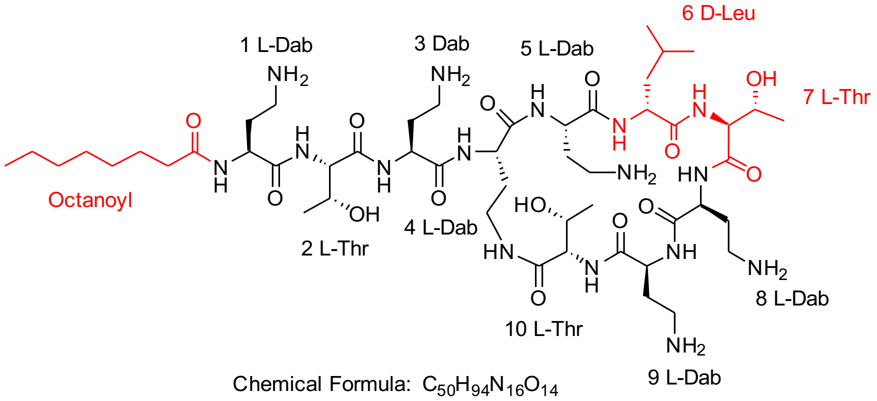

TFA salt was obtained in a yield of 54.7 mg, retention time (t_R) at 214 nm = 11.345 min (HPLC purity: 98.4%). ESI-MS analysis of peak at 11.345 min: *m/z* (monoisotopic) $[M+H]^+$ 1144.0, $[M+2H]^{2+}$ 572.8 $[M+3H]^{3+}$ 382.3. Calculated *m/z* (monoisotopic) for **F100** ($C_{50}H_{94}N_{16}O_{14}$) $[M+H]^+$ 1143.7, $[M+2H]^{2+}$ 572.4, $[M+3H]^{3+}$ 381.9. ^{1}H NMR (600 MHz, D_2O) δ 4.54 – 4.43 (m, 3H), 4.42 – 4.12 (m, 10H), 3.35 – 3.26 (m, 1H), 3.19 – 3.13 (m, 1H), 3.13 – 2.92 (m, 10H), 2.33 – 1.77 (m, 15H), 1.68 – 1.50 (m, 5H), 1.27 – 1.19 (m, 8H), 1.18 – 1.10 (m, 9H), 0.94 – 0.83 (m, 6H), 0.83 – 0.77 (m, 3H).

F124:

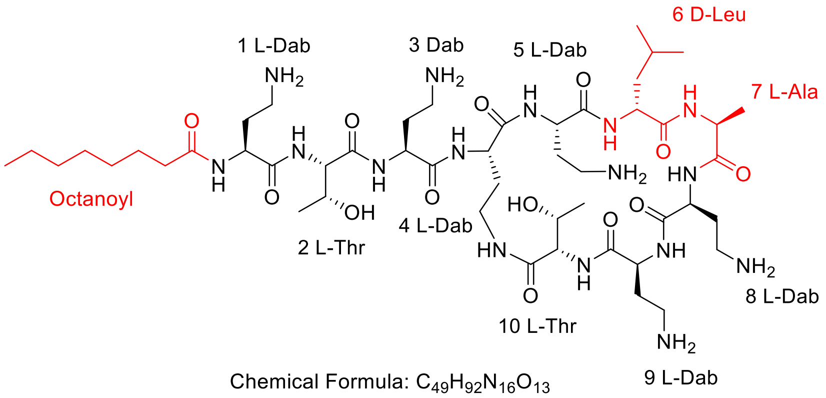

TFA salt was obtained in a yield of 57.0 mg, retention time (t_R) at 214 nm = 11.504 min (HPLC purity: 99.2%). ESI-MS analysis of peak at 11.504 min: *m/z* (monoisotopic) $[M+H]^+$ 1114.3, $[M+2H]^{2+}$ 557.8 $[M+3H]^{3+}$ 372.3. Calculated *m/z* (monoisotopic) for **F124** ($C_{49}H_{92}N_{16}O_{13}$) $[M+H]^+$ 1113.7, $[M+2H]^{2+}$ 557.4, $[M+3H]^{3+}$ 371.9. 1H NMR (600 MHz, D_2O) δ 4.50 – 4.47 (m, 1H), 4.46 – 4.42 (m, 2H), 4.37 – 4.12 (m, 9H), 3.31 – 3.25 (m, 1H), 3.17 – 3.11 (m, 1H), 3.11 – 2.91 (m, 10H), 2.28 – 2.24 (m, 2H), 2.22 – 1.76 (m, 12H), 1.62 – 1.49 (m, 5H), 1.36 – 1.30 (m, 3H), 1.25 – 1.17 (m, 8H), 1.17 – 1.12 (m, 6H), 0.89 – 0.81 (m, 6H), 0.81 – 0.76 (m, 3H).

F183:

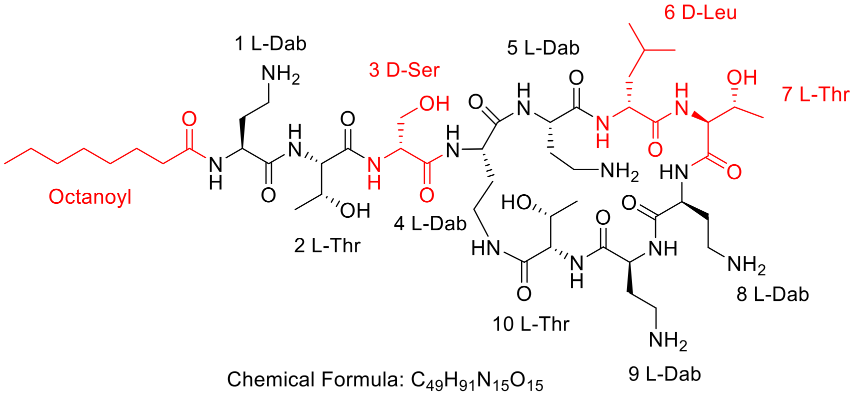

TFA salt was obtained in a yield of 42.9 mg, retention time (t_R) at 214 nm = 11.740 min (HPLC purity: 98.7%). ESI-MS analysis of peak at 11.740 min: *m/z* (monoisotopic) $[M+H]^+$ 1131.1, $[M+2H]^{2+}$ 566.2 $[M+3H]^{3+}$ 377.9. Calculated *m/z* (monoisotopic) for **F183** ($C_{49}H_{91}N_{15}O_{15}$) $[M+H]^+$ 1130.7, $[M+2H]^{2+}$ 565.8, $[M+3H]^{3+}$ 377.6. ^{1}H NMR (600 MHz, D_2O) δ 4.51 – 4.43 (m, 2H), 4.42 – 4.38 (m, 1H), 4.38 – 4.08 (m, 10H), 3.91 – 3.79 (m, 2H), 3.37 – 3.27 (m, 1H), 3.15 – 2.91 (m, 10H), 2.30 – 2.24 (m, 3H), 2.23 – 1.77 (m, 10H), 1.67 – 1.49 (m, 6H), 1.25 – 1.18 (m, 8H), 1.17 – 1.10 (m, 9H), 0.92 – 0.83 (m, 6H), 0.81 – 0.76 (m, 3H).

F224:

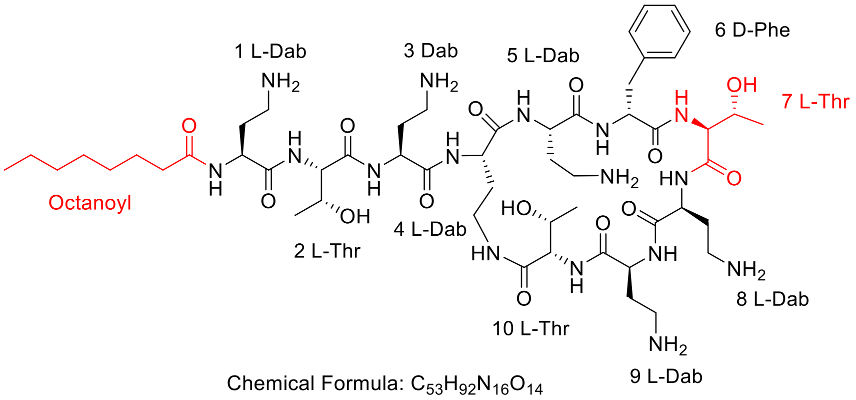

TFA salt was obtained in a yield of 54.8 mg, retention time (t_R) at 214 nm = 11.595 min (HPLC purity: 99.3%). ESI-MS analysis of peak at 11.595 min: *m/z* (monoisotopic) $[M+H]^+$ 1178.4, $[M+2H]^{2+}$ 589.8 $[M+3H]^{3+}$ 393.7. Calculated *m/z* (monoisotopic) for **F224** ($C_{53}H_{92}N_{16}O_{14}$) $[M+H]^+$ 1177.7, $[M+2H]^{2+}$ 589.4, $[M+3H]^{3+}$ 393.2. ^{1}H NMR (600 MHz, D_2O) δ 7.35 – 7.22 (m, 5H), 4.46 – 4.39 (m, 3H), 4.30 – 4.14 (m, 8H), 4.13 – 4.11 (m, 1H), 3.30 – 3.22 (m, 1H), 3.15 – 2.96 (m, 12H), 2.79 – 2.60 (m, 2H), 2.27 – 2.24 (m, 2H), 2.21 – 1.97 (m, 8H), 1.94 – 1.76 (m, 4H), 1.57 – 1.49 (m, 2H), 1.25 – 1.17 (m, 9H), 1.16 – 1.11 (m, 6H), 0.81 – 0.77 (m, 6H).

F225:

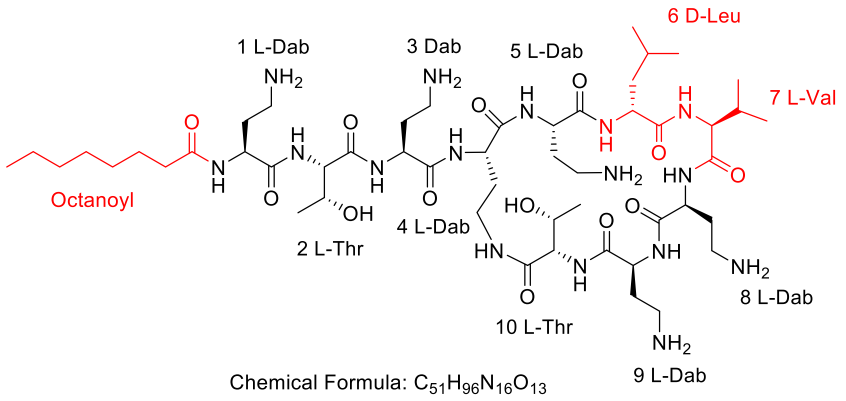

TFA salt was obtained in a yield of 41.2 mg, retention time (t_R) at 214 nm = 11.767 min (HPLC purity: 99.3%). ESI-MS analysis of peak at 11.767 min: *m/z* (monoisotopic) $[M+H]^+$ 1142.4, $[M+2H]^{2+}$ 571.8, $[M+3H]^{3+}$ 381.7. Calculated *m/z* (monoisotopic) for **F225** ($C_{51}H_{96}N_{16}O_{13}$) $[M+H]^+$ 1141.7, $[M+2H]^{2+}$ 571.4, $[M+3H]^{3+}$ 381.2. ^1H NMR (600 MHz, D_2O) δ 4.54 – 4.49 (m, 1H), 4.46 – 4.41 (m, 2H), 4.36 – 4.31 (m, 1H), 4.31 – 4.28 (m, 1H), 4.27 – 4.12 (m, 6H), 3.31 – 3.11 (m, 2H), 3.10 – 2.91 (m, 10H), 2.29 – 2.23 (m, 3H), 2.23 – 1.88 (m, 11H), 1.84 – 1.76 (m, 1H), 1.63 – 1.46 (m, 5H), 1.25 – 1.18 (m, 8H), 1.17 – 1.12 (m, 6H), 0.90 – 0.77 (m, 15H).

F226:

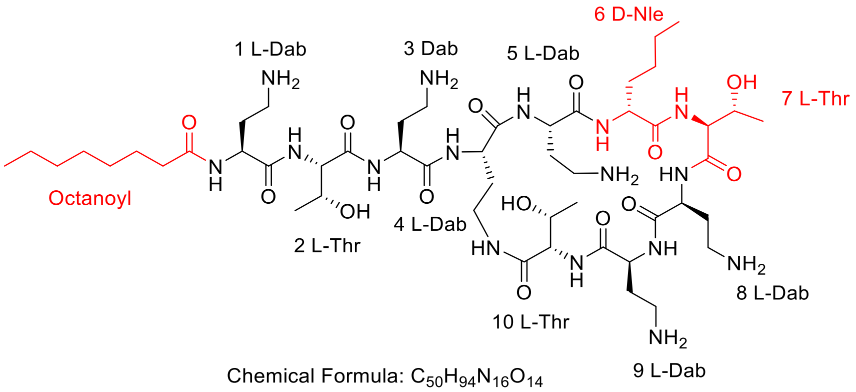

TFA salt was obtained in a yield of 61.2 mg, retention time (t_R) at 214 nm = 11.555 min (HPLC purity: 98.9%). ESI-MS analysis of peak at 11.555 min: *m/z* (monoisotopic) $[M+H]^+$ 1144.4, $[M+2H]^{2+}$ 572.8, $[M+3H]^{3+}$ 382.3. Calculated *m/z* (monoisotopic) for **F226** ($C_{50}H_{94}N_{16}O_{14}$) $[M+H]^+$ 1143.7, $[M+2H]^{2+}$ 572.4, $[M+3H]^{3+}$ 381.9. ^1H NMR (600 MHz, D_2O) δ 4.52 – 4.48 (m, 1H), 4.46 – 4.42 (m, 2H), 4.40 – 4.36 (m, 2H), 4.32 – 4.22 (m, 5H), 4.20 – 4.14 (m, 2H), 4.13 – 4.11 (m, 1H), 3.34 – 3.27 (m, 1H), 3.16 – 2.92 (m, 11H), 2.28 – 2.24 (m, 3H), 2.22 – 1.96 (m, 9H), 1.96 – 1.87 (m, 1H), 1.86 – 1.65 (m, 3H), 1.59 – 1.49 (m, 2H), 1.32 – 1.17 (m, 12H), 1.17 – 1.11 (m, 9H), 0.85 – 0.75 (m, 6H).

F227:

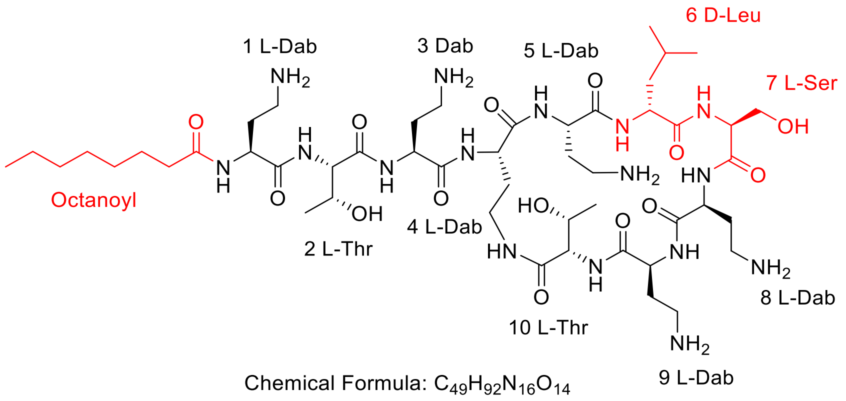

TFA salt was obtained in a yield of 49.8 mg, retention time (t_R) at 214 nm = 11.341 min (HPLC purity: 99.2%). ESI-MS analysis of peak at 11.341 min: *m/z* (monoisotopic) $[M+H]^+$ 1130.3, $[M+2H]^{2+}$ 565.8 $[M+3H]^{3+}$ 377.6. Calculated *m/z* (monoisotopic) for **F227** ($C_{49}H_{92}N_{16}O_{14}$) $[M+H]^+$ 1129.7, $[M+2H]^{2+}$ 565.4, $[M+3H]^{3+}$ 377.2. ^{1}H NMR (600 MHz, D_2O) δ 4.68 – 4.52 (m, 1H), 4.52 – 4.12 (m, 11H), 3.83 (ddd, J = 64.3, 11.3, 5.5 Hz, 2H), 3.33 – 3.21 (m, 1H), 3.18 – 2.90 (m, 11H), 2.29 – 2.22 (m, 3H), 2.19 – 1.97 (m, 9H), 1.94 – 1.77 (m, 2H), 1.65 – 1.43 (m, 5H), 1.26 – 1.17 (m, 8H), 1.16 – 1.10 (m, 6H), 0.85 (dd, J = 25.1, 6.0 Hz, 6H), 0.81 – 0.52 (m, 3H).

F228:

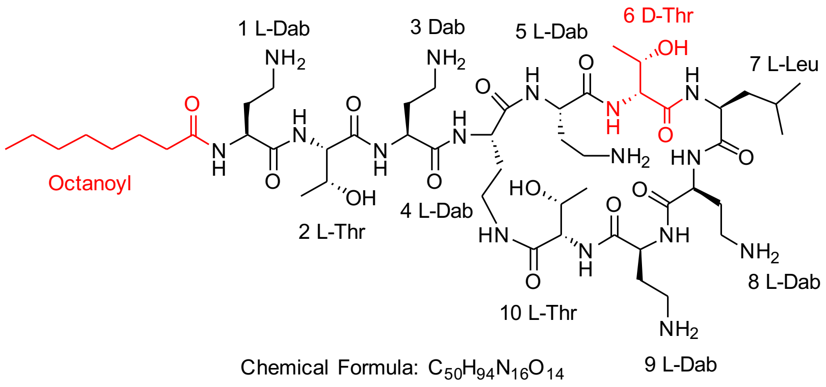

TFA salt was obtained in a yield of 52.4 mg, retention time (t_R) at 214 nm = 11.410 min (HPLC purity: 99.0%). ESI-MS analysis of peak at 11.410 min: *m/z* (monoisotopic) $[M+H]^+$ 1144.4, $[M+2H]^{2+}$ 572.8 $[M+3H]^{3+}$ 382.3. Calculated *m/z* (monoisotopic) for **F228** ($C_{50}H_{94}N_{16}O_{14}$) $[M+H]^+$ 1143.7, $[M+2H]^{2+}$ 572.4, $[M+3H]^{3+}$ 381.9. ^{1}H NMR (600 MHz, D_2O) δ 4.58 (dd, J = 9.3, 5.0 Hz, 1H), 4.48 – 4.42 (m, 2H), 4.35 – 4.08 (m, 10H), 3.33 – 3.11 (m, 2H), 3.09 – 2.94 (m, 11H), 2.29 – 2.24 (m, 2H), 2.24 – 1.97 (m, 11H), 1.97 – 1.79 (m, 2H), 1.67 – 1.50 (m, 5H), 1.25 – 1.18 (m, 8H), 1.18 – 1.12 (m, 9H), 0.87 (d, J = 6.1 Hz, 3H), 0.82 (d, J = 6.0 Hz, 3H), 0.79 (t, J = 7.0 Hz, 3H).

F229:

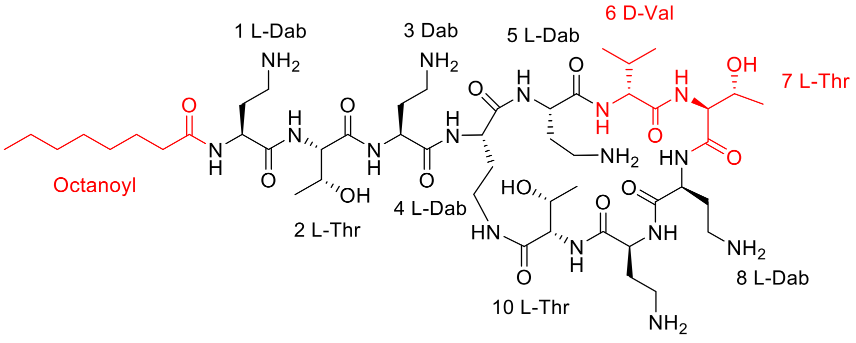

Chemical Formula: $C_{49}H_{92}N_{16}O_{14}$

TFA salt was obtained in a yield of 67.0 mg, retention time (t_R) at 214 nm = 11.264 min (HPLC purity: 97.2%). ESI-MS analysis of peak at 11.264 min: *m/z* (monoisotopic) $[M+H]^+$ 1130.4, $[M+2H]^{2+}$ 565.9 $[M+3H]^{3+}$ 377.6. Calculated *m/z* (monoisotopic) for **F229** ($C_{49}H_{92}N_{16}O_{14}$) $[M+H]^+$ 1129.7, $[M+2H]^{2+}$ 565.4, $[M+3H]^{3+}$ 377.2. ^{1}H NMR (600 MHz, D*2*O) δ 4.55 (dd, J = 9.3, 5.0 Hz, 1H), 4.44 (dd, J = 9.3, 5.2 Hz, 2H), 4.41 – 4.35 (m, 2H), 4.31 – 4.26 (m, 3H), 4.26 – 4.22 (m, 1H), 4.21 – 4.15 (m, 2H), 4.11 (d, J = 4.9 Hz, 1H), 4.09 (d, J = 7.6 Hz, 1H), 3.35 – 3.27 (m, 1H), 3.17 – 2.93 (m, 12H), 2.28 – 2.24 (m, 2H), 2.23 – 1.95 (m, 12H), 1.94 – 1.77 (m, 2H), 1.58 – 1.48 (m, 2H), 1.25 – 1.18 (m, 8H), 1.15 (dd, J = 12.5, 6.3 Hz, 9H), 0.97 – 0.91 (m, 6H), 0.79 (t, J = 6.9 Hz, 3H).

F230:

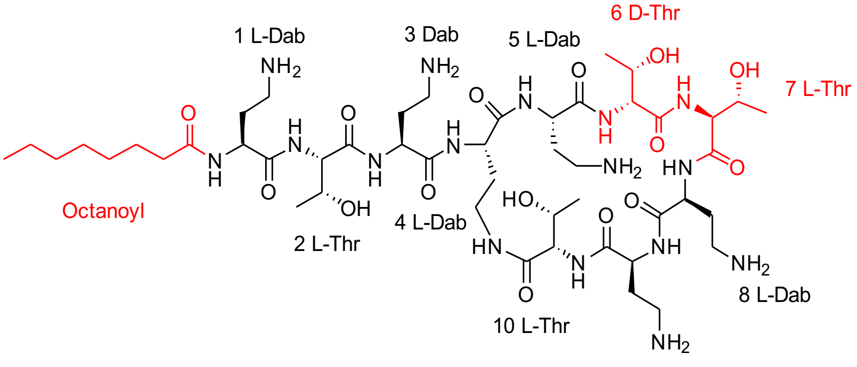

Chemical Formula: $C_{48}H_{90}N_{16}O_{15}$

TFA salt was obtained in a yield of 47.1 mg, retention time (t_R) at 214 nm = 11.097 min (HPLC purity: 98.1%). ESI-MS analysis of peak at 11.097 min: *m/z* (monoisotopic) $[M+H]^+$ 1132.3, $[M+2H]^{2+}$ 566.9, $[M+3H]^{3+}$ 378.2. Calculated *m/z* (monoisotopic) for **F230** ($C_{48}H_{90}N_{16}O_{15}$) $[M+H]^+$ 1131.7, $[M+2H]^{2+}$ 566.4, $[M+3H]^{3+}$ 377.9. ^{1}H NMR (600 MHz, D_2O) δ 4.58 (dd, J = 9.3, 5.1 Hz, 1H), 4.44 (dd, J = 9.3, 5.3 Hz, 2H), 4.39 (d, J = 3.2 Hz, 1H), 4.36 (dd, J = 6.4, 3.2 Hz, 1H), 4.33 – 4.27 (m, 4H), 4.24 (dd, J = 10.2, 4.3 Hz, 1H), 4.20 – 4.13 (m, 4H), 4.11 (d, J = 5.0 Hz, 1H), 3.35 – 3.24 (m, 1H), 3.17 – 2.95 (m, 14H), 2.31 – 2.22 (m, 3H), 2.22 – 1.97 (m, 11H), 1.96 – 1.78 (m, 4H), 1.60 – 1.47 (m, 12H), 1.24 – 1.21 (m, 2H), 1.21 – 1.18 (m, 3H), 1.18 – 1.10 (m, 22H), 0.79 (t, J = 7.0 Hz, 3H).

F251:

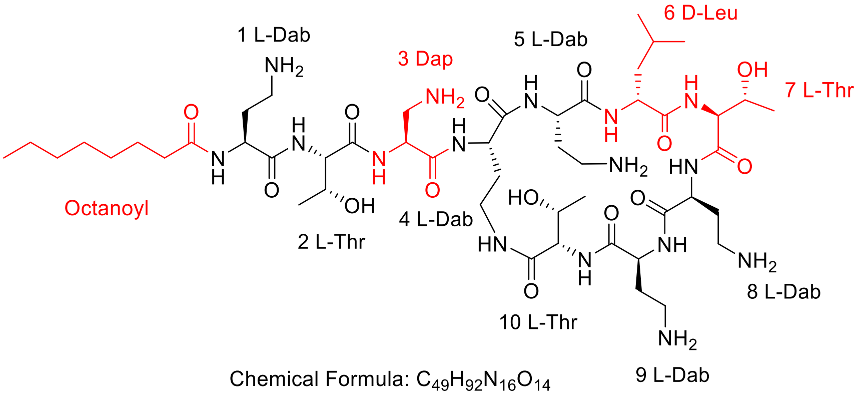

TFA salt was obtained in a yield of 72.2 mg, retention time (t_R) at 214 nm = 11.424 min (HPLC purity: 98.8%). ESI-MS analysis of peak at 11.424 min: *m/z* (monoisotopic) $[M+H]^+$ 1130.4, $[M+2H]^{2+}$ 565.8, $[M+3H]^{3+}$ 377.7. Calculated *m/z* (monoisotopic) for **F251** ($C_{49}H_{92}N_{16}O_{14}$) $[M+H]^+$ 1129.7, $[M+2H]^{2+}$ 565.4, $[M+3H]^{3+}$ 377.2. ^1H NMR (600 MHz, D_2O) δ 4.47 (ddd, J = 22.0, 9.4, 5.2 Hz, 2H), 4.41 – 4.34 (m, 3H), 4.32 – 4.21 (m, 4H), 4.20 – 4.15 (m, 1H), 4.12 (d, J = 4.9 Hz, 1H), 3.48 – 3.41 (m, 1H), 3.35 – 3.24 (m, 2H), 3.18 – 2.91 (m, 9H), 2.30 – 2.24 (m, 3H), 2.25 – 1.98 (m, 8H), 1.96 – 1.75 (m, 2H), 1.68 – 1.49 (m, 5H), 1.26 – 1.18 (m, 8H), 1.17 – 1.10 (m, 9H), 0.87 (dd, J = 26.6, 5.8 Hz, 6H), 0.79 (t, J = 7.0 Hz, 3H).

F252:

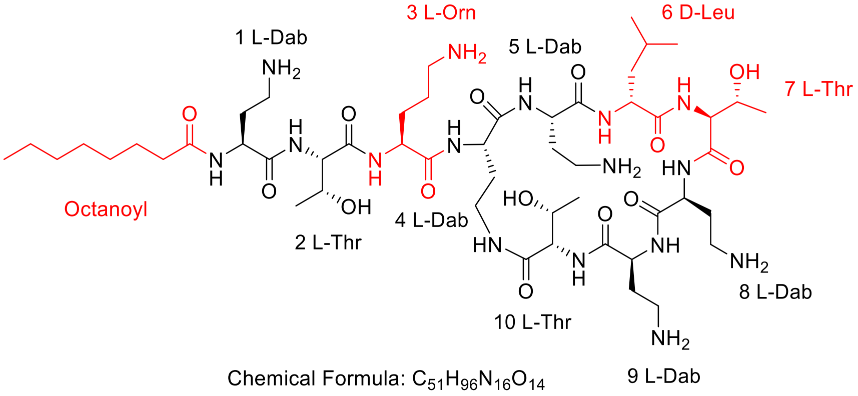

TFA salt was obtained in a yield of 57.9 mg, retention time (t_R) at 214 nm = 11.346 min (HPLC purity: 98.4%). ESI-MS analysis of peak at 11.346 min: *m/z* (monoisotopic) $[M+H]^+$ 1158.4, $[M+2H]^{2+}$ 579.9, $[M+3H]^{3+}$ 387.0. Calculated *m/z* (monoisotopic) for **F252** ($C_{51}H_{96}N_{16}O_{14}$) $[M+H]^+$ 1157.7, $[M+2H]^{2+}$ 579.4, $[M+3H]^{3+}$ 386.6. ^1H NMR (600 MHz, D_2O) δ 4.49 (dd, J = 9.3, 4.8 Hz, 1H), 4.43 (dd, J = 9.2, 5.2 Hz, 1H), 4.40 – 4.27 (m, 6H), 4.27 – 4.20 (m, 2H), 4.19 – 4.15 (m, 2H), 4.13 (d, J = 5.0 Hz, 1H), 3.33 – 3.25 (m, 1H), 3.16 – 2.91 (m, 12H), 2.30 – 2.22 (m, 3H), 2.22 – 2.09 (m, 5H), 2.08 – 1.80 (m, 6H), 1.77 – 1.47 (m, 9H), 1.25 – 1.16 (m, 9H), 1.14 (d, J = 14.6 Hz, 9H), 0.87 (dd, J = 24.8, 5.4 Hz, 6H), 0.79 (t, J = 6.5 Hz, 3H).

F271:

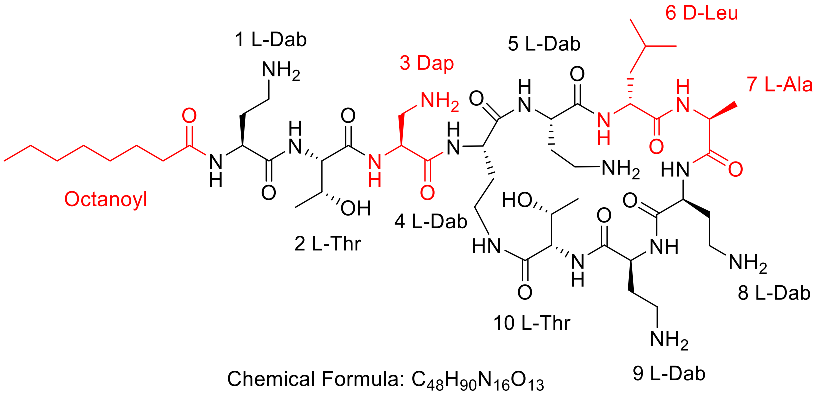

TFA salt was obtained in a yield of 59.3 mg, retention time (t_R) at 214 nm = 11.373 min (HPLC purity: 98.7%). ESI-MS analysis of peak at 11.373 min: *m/z* (monoisotopic) $[M+H]^+$ 1099.9, $[M+2H]^{2+}$ 550.8, $[M+3H]^{3+}$ 367.6. Calculated *m/z* (monoisotopic) for **F271** ($C_{48}H_{90}N_{16}O_{13}$) $[M+H]^+$ 1099.7, $[M+2H]^{2+}$ 550.3 $[M+3H]^{3+}$ 367.2. ^{1}H NMR (600 MHz, D_2O) δ 4.50 – 4.44 (m, 2H), 4.37 (d, J = 3.8 Hz, 1H), 4.31 (q, J = 7.2 Hz, 1H), 4.28 – 4.19 (m, 6H), 4.16 (d, J = 4.4 Hz, 1H), 3.49 – 3.43 (m, 1H), 3.33 – 3.23 (m, 2H), 3.16 – 2.92 (m, 10H), 2.29 – 2.24 (m, 2H), 2.23 – 2.07 (m, 6H), 2.05 – 1.90 (m, 3H), 1.86 – 1.79 (m, 1H), 1.62 – 1.48 (m, 5H), 1.33 (d, J = 7.2 Hz, 3H), 1.25 – 1.17 (m, 8H), 1.14 (d, J = 13.4 Hz, 6H), 0.84 (dd, J = 26.0, 6.0 Hz, 6H), 0.79 (t, J = 6.9 Hz, 3H).

F287:

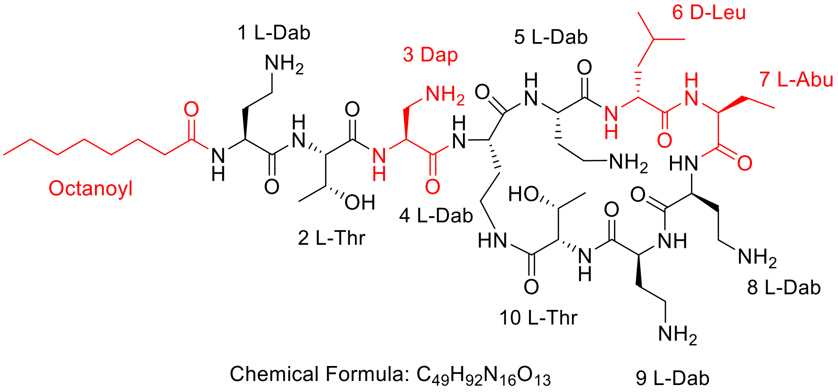

TFA salt was obtained in a yield of 52.5 mg, retention time (t_R) at 214 nm = 11.509 min (HPLC purity: 98.9%). ESI-MS analysis of peak at 11.509 min: *m/z* (monoisotopic) $[M+H]^+$ 1113.9, $[M+2H]^{2+}$ 557.8, $[M+3H]^{3+}$ 372.2. Calculated *m/z* (monoisotopic) for **F287** ($C_{49}H_{92}N_{16}O_{13}$) $[M+H]^+$ 1113.7, $[M+2H]^{2+}$ 557.4, $[M+3H]^{3+}$ 371.9. ^{1}H NMR (600 MHz, D_2O) δ 4.47 (ddd, J = 22.7, 9.3, 5.1 Hz, 2H), 4.37 (d, J = 3.7 Hz, 1H), 4.29 – 4.17 (m, 6H), 4.15 (d, J = 4.6 Hz, 1H), 3.46 (dd, J = 13.4, 4.7 Hz, 1H), 3.33 – 3.23 (m, 2H), 3.16 – 2.92 (m, 10H), 2.29 – 2.25 (m, 2H), 2.24 – 1.78 (m, 12H), 1.67 – 1.58 (m, 2H), 1.57 – 1.50 (m, 4H), 1.25 – 1.18 (m, 8H), 1.15 (d, J = 11.3 Hz, 6H), 0.89 – 0.85 (m, 6H), 0.83 (d, J = 5.8 Hz, 3H), 0.79 (t, J = 6.7 Hz, 3H).

F300:

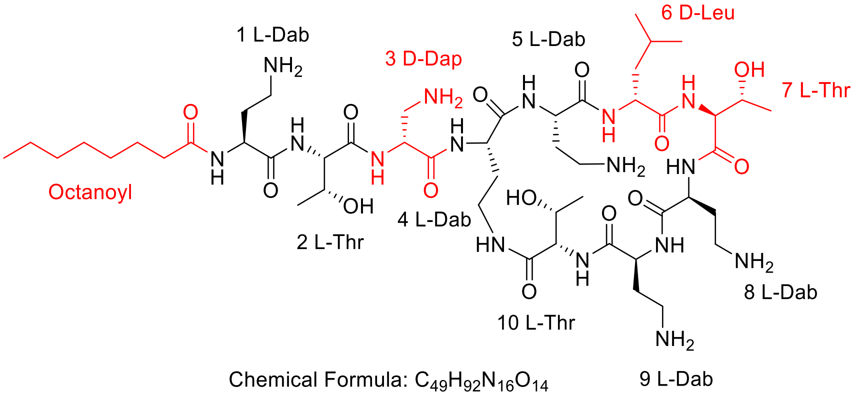

TFA salt was obtained in a yield of 40.0 mg, retention time (t_R) at 214 nm = 11.616 min (HPLC purity: 97.3%). ESI-MS analysis of peak at 11.616 min: *m/z* (monoisotopic) $[M+H]^+$ 1129.9, $[M+2H]^{2+}$ 565.8, $[M+3H]^{3+}$ 377.6. Calculated *m/z* (monoisotopic) for **F300** ($C_{49}H_{92}N_{16}O_{14}$) $[M+H]^+$ 1129.7, $[M+2H]^{2+}$ 565.4, $[M+3H]^{3+}$ 377.2. ^{1}H NMR (600 MHz, D_2O) δ 4.47 (dt, J = 9.1, 4.5 Hz, 2H), 4.40 – 4.34 (m, 4H), 4.31 – 4.26 (m, 3H), 4.24 (dd, J = 10.1, 4.5 Hz, 1H), 4.17 (dd, J = 12.0, 5.8 Hz, 1H), 4.12 (d, J = 5.0 Hz, 1H), 3.49 (dd, J = 13.4, 5.5 Hz, 1H), 3.36 – 3.24 (m, 2H), 3.15 – 2.93 (m, 10H), 2.29 – 2.25 (m, 3H), 2.25 – 1.98 (m, 9H), 1.97 – 1.80 (m, 2H), 1.68 – 1.50 (m, 6H), 1.25 – 1.18 (m, 9H), 1.15 (dd, J = 15.5, 9.2 Hz, 9H), 0.87 (dd, J = 26.3, 5.8 Hz, 6H), 0.79 (t, J = 6.9 Hz, 3H).

F314:

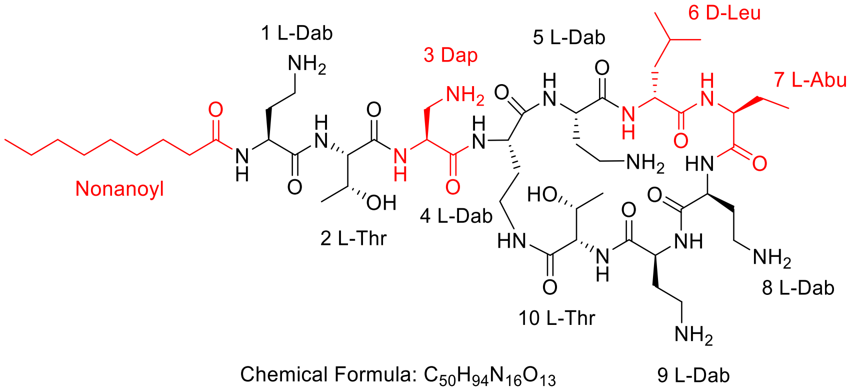

TFA salt was obtained in a yield of 67.6 mg, retention time (t_R) at 214 nm = 11.644 min (HPLC purity: 99.5%). ESI-MS analysis of peak at 11.644 min: *m/z* (monoisotopic) $[M+H]^+$ 1128.1, $[M+2H]^{2+}$ 564.9, $[M+3H]^{3+}$ 376.9. Calculated *m/z* (monoisotopic) for **F314** ($C_{50}H_{94}N_{16}O_{13}$) $[M+H]^+$ 1127.7, $[M+2H]^{2+}$ 564.4, $[M+3H]^{3+}$ 376.6. ^{1}H NMR (600 MHz, D_2O) δ 4.50 – 4.43 (m, 2H), 4.37 – 4.35 (m, 1H), 4.28 – 4.12 (m, 9H), 3.48 – 3.42 (m, 1H), 3.31 – 3.22 (m, 3H), 3.14 – 2.91 (m, 11H), 2.27 – 2.07 (m, 10H), 2.04 – 1.76 (m, 6H), 1.67 – 1.50 (m, 7H), 1.24 – 1.17 (m, 12H), 1.16 – 1.12 (m, 7H), 0.89 – 0.85 (m, 7H), 0.84 – 0.81 (m, 4H), 0.80 – 0.76 (m, 4H).

F319:

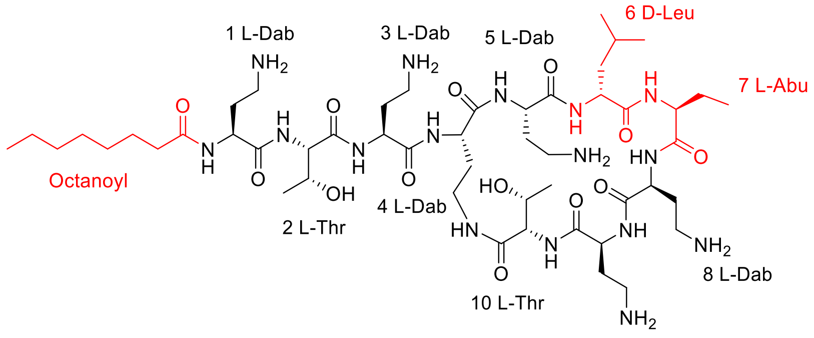

Chemical Formula: $C_{50}H_{94}N_{16}O_{13}$

TFA salt was obtained in a yield of 23.0 mg, retention time (t_R) at 214 nm = 11.914 min (HPLC purity: 99.5%). ESI-MS analysis of peak at 11.914 min: *m/z* (monoisotopic) $[M+H]^+$ 1127.9, $[M+2H]^{2+}$ 564.8, $[M+3H]^{3+}$ 376.9. Calculated *m/z* (monoisotopic) for **F319** ($C_{50}H_{94}N_{16}O_{13}$) $[M+H]^+$ 1127.7, $[M+2H]^{2+}$ 564.4, $[M+3H]^{3+}$ 376.6. ^{1}H NMR (600 MHz, D_2O) δ 4.51 (dd, J = 9.4, 4.9 Hz, 1H), 4.46 (dd, J = 9.4, 5.2 Hz, 2H), 4.31 (d, J = 4.4 Hz, 1H), 4.30 – 4.14 (m, 9H), 3.32 – 3.25 (m, 1H), 3.19 – 2.93 (m, 13H), 2.30 – 2.26 (m, 3H), 2.26 – 1.99 (m, 12H), 1.96 – 1.79 (m, 4H), 1.70 – 1.51 (m, 7H), 1.26 – 1.19 (m, 10H), 1.16 (dd, J = 6.3, 1.5 Hz, 6H), 0.90 – 0.86 (m, 6H), 0.85 (d, J = 6.0 Hz, 3H), 0.81 (t, J = 6.9 Hz, 3H).

F342:

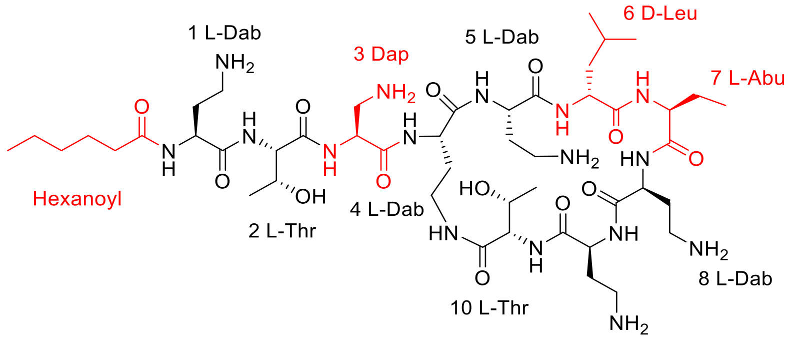

Chemical Formula: $C_{47}H_{88}N_{16}O_{13}$

TFA salt was obtained in a yield of 69.9 mg, retention time (t_R) at 214 nm = 10.689 min (HPLC purity: 99.3%). ESI-MS analysis of peak at 10.689 min: *m/z* (monoisotopic) $[M+H]^+$ 1085.9, $[M+2H]^{2+}$ 543.8, $[M+3H]^{3+}$ 362.9. Calculated *m/z* (monoisotopic) for **F342** ($C_{47}H_{88}N_{16}O_{13}$) $[M+H]^+$ 1085.7, $[M+2H]^{2+}$ 543.3, $[M+3H]^{3+}$ 362.6. ^{1}H NMR (600 MHz, D_2O) δ 4.48 (ddd, J = 24.0, 11.9, 7.2 Hz, 2H), 4.37 (dd, J = 9.1, 3.8 Hz, 1H), 4.31 – 4.13 (m, 9H), 3.52 – 3.42 (m, 1H), 3.38 – 3.23 (m, 2H), 3.19 – 2.86 (m, 11H), 2.29 – 2.24 (m, 3H), 2.24 – 2.07 (m, 7H), 2.06 – 1.77 (m, 6H), 1.68 – 1.49 (m, 7H), 1.26 – 1.19 (m, 5H), 1.15 (dd, J = 13.2, 7.6 Hz, 6H), 0.90 – 0.85 (m, 6H), 0.84 – 0.82 (m, 3H), 0.81 – 0.78 (m, 3H).

F350:

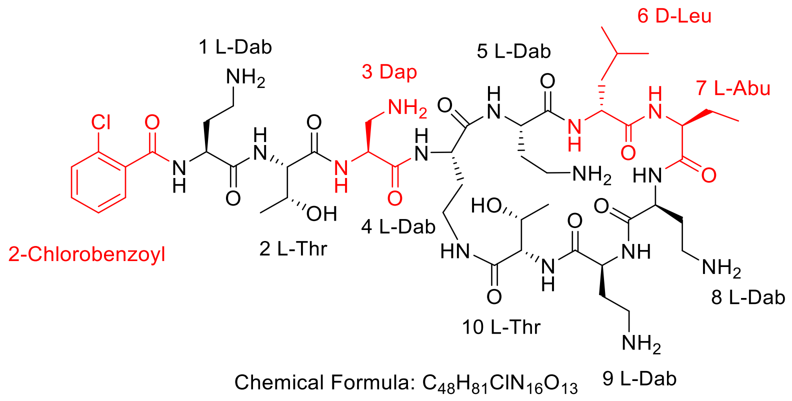

TFA salt was obtained in a yield of 61.0 mg, retention time (t_R) at 214 nm = 10.561 min (HPLC purity: 97.3%). ESI-MS analysis of peak at 10.561 min: *m/z* (monoisotopic) $[M+H]^+$ 1125.8, $[M+2H]^{2+}$ 564.0, $[M+3H]^{3+}$ 376.4. Calculated *m/z* (monoisotopic) for **F350** ($C_{48}H_{81}ClN_{16}O_{13}$) $[M+H]^+$ 1125.6, $[M+2H]^{2+}$ 563.3, $[M+3H]^{3+}$ 375.9. ^{1}H NMR (600 MHz, D_2O) δ 7.52 – 7.42 (m, 3H), 7.39 (dd, J = 12.8, 6.5 Hz, 1H), 4.49 (dd, J = 9.2, 5.2 Hz, 1H), 4.43 (d, J = 3.7 Hz, 1H), 4.32 – 4.22 (m, 5H), 4.19 (dt, J = 12.9, 5.3 Hz, 2H), 4.14 (t, J = 5.1 Hz, 1H), 3.51 – 3.43 (m, 1H), 3.35 – 3.21 (m, 2H), 3.20 – 2.90 (m, 10H), 2.34 – 2.07 (m, 7H), 2.05 – 1.78 (m, 4H), 1.68 – 1.49 (m, 4H), 1.32 – 1.11 (m, 6H), 0.92 – 0.80 (m, 9H).

F360:

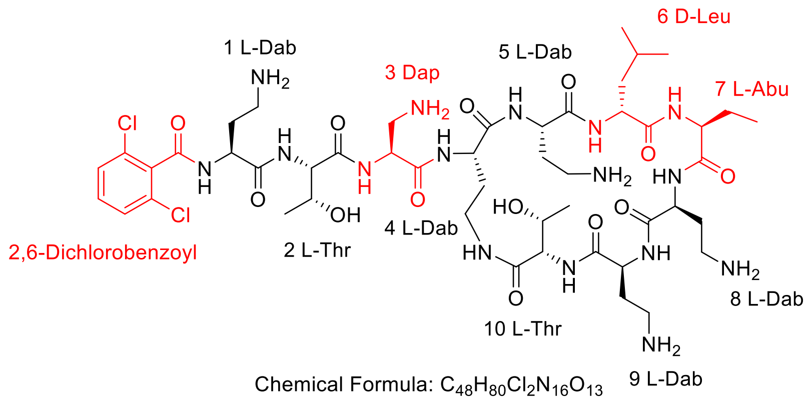

TFA salt was obtained in a yield of 54.2 mg, retention time (t_R) at 214 nm = 10.648 min (HPLC purity: 98.1%). ESI-MS analysis of peak at 10.648 min: *m/z* (monoisotopic) $[M+H]^+$ 1159.8, $[M+2H]^{2+}$ 580.7, $[M+3H]^{3+}$ 387.9. Calculated *m/z* (monoisotopic) for **F360** ($C_{48}H_{80}Cl_2N_{16}O_{13}$) $[M+H]^+$ 1159.5, $[M+2H]^{2+}$ 580.3, $[M+3H]^{3+}$ 387.2. ^{1}H NMR (600 MHz, D_2O) δ 7.52 – 7.42 (m, 3H), 7.39 (dd, J = 12.8, 6.5 Hz, 1H), 4.49 (dd, J = 9.2, 5.2 Hz, 1H), 4.43 (d, J = 3.7 Hz, 1H), 4.32 – 4.22 (m, 6H), 4.19 (dt, J = 12.9, 5.3 Hz, 2H), 4.14 (t, J = 5.1 Hz, 1H), 3.51 – 3.43 (m, 1H), 3.35 – 3.21 (m, 2H), 3.20 – 2.90 (m, 10H), 2.34 – 2.07 (m, 7H), 2.05 – 1.78 (m, 4H), 1.68 – 1.49 (m, 4H), 1.32 – 1.11 (m, 6H), 0.92 – 0.80 (m, 9H).

F365 (QPX9003):

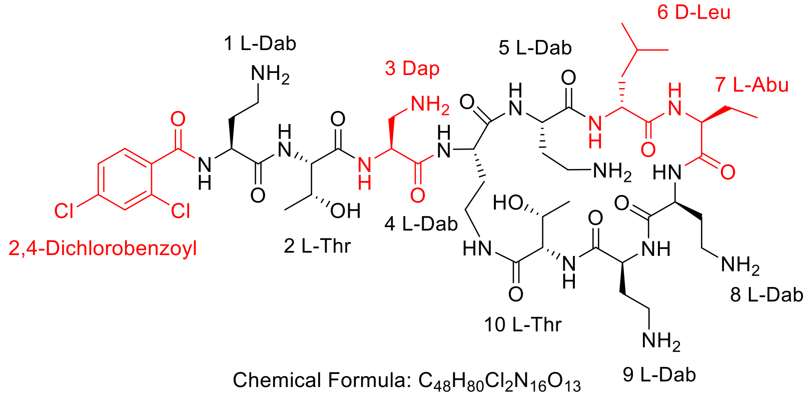

TFA salt was obtained in a yield of 63.5 mg, retention time (t_R) at 214 nm = 11.068 min (HPLC purity: 99.0%). ESI-MS analysis of peak at 11.068 min: *m/z* (monoisotopic) $[M+H]^+$ 1159.8, $[M+2H]^{2+}$ 580.7, $[M+3H]^{3+}$ 387.9. Calculated *m/z* (monoisotopic) for **F365** ($C_{48}H_{80}Cl_2N_{16}O_{13}$) $[M+H]^+$ 1159.5, $[M+2H]^{2+}$ 580.3, $[M+3H]^{3+}$ 387.2. HRMS: *m/z* monoisotopic calculated for $C_{48}H_{80}Cl_2N_{16}O_{13}$ $[M+H]^+$ 1159.5546, observed 1159.5544.^{1}H NMR (600 MHz, D_2O) δ 7.59 (d, J = 1.6 Hz, 1H), 7.45 – 7.40 (m, 2H), 4.49 (dd, J = 9.4, 5.0 Hz, 2H), 4.31 – 4.11 (m, 10H), 3.47 (dd, J = 13.4, 5.0 Hz, 2H), 3.32 – 3.24 (m, 2H), 3.12 (d, J = 8.3 Hz, 3H), 3.08 – 2.95 (m, 6H), 2.30 – 2.16 (m, 4H), 2.15 – 2.08 (m, 4H), 2.03 – 1.78 (m, 6H), 1.58 (dddd, J = 24.0, 19.6, 15.1, 8.5 Hz, 5H), 1.18 (d, J = 6.4 Hz, 3H), 1.13 (d, J = 6.4 Hz, 3H), 0.89 – 0.85 (m, 6H), 0.83 (d, J = 6.0 Hz, 3H).

F371:

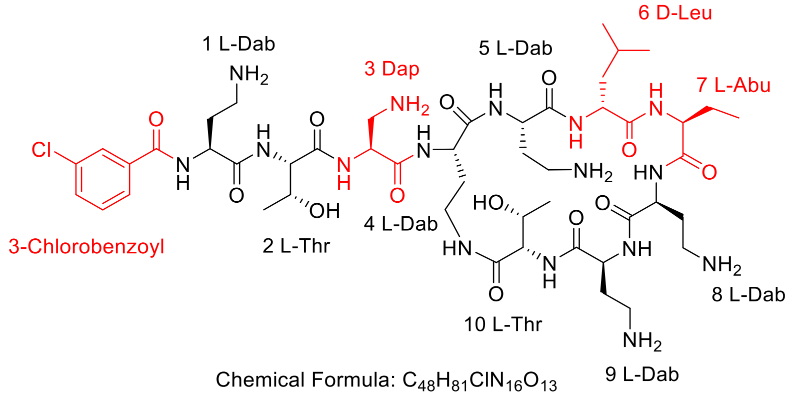

TFA salt was obtained in a yield of 59.3 mg, retention time (t_R) at 214 nm = 10.781 min (HPLC purity: 98.5%). ESI-MS analysis of peak at 10.781 min: *m/z* (monoisotopic) $[M+H]^+$ 1125.9, $[M+2H]^{2+}$ 564.0, $[M+3H]^{3+}$ 376.4. Calculated *m/z* (monoisotopic) for **F371** ($C_{48}H_{81}ClN_{16}O_{13}$) $[M+H]^+$ 1125.6, $[M+2H]^{2+}$ 563.3, $[M+3H]^{3+}$ 375.9. ^{1}H NMR (600 MHz, D_2O) δ 7.84 – 7.73 (m, 1H), 7.71 – 7.53 (m, 2H), 7.50 – 7.40 (m, 1H), 4.52 – 4.34 (m, 2H), 4.34 – 4.05 (m, 7H), 3.50 – 3.41 (m, 1H), 3.37 – 3.21 (m, 2H), 3.18 – 2.88 (m, 9H), 2.37 – 2.09 (m, 7H), 2.02 – 1.71 (m, 4H), 1.67 – 1.48 (m, 4H), 1.19 – 1.07 (m, 6H), 0.92 – 0.78 (m, 9H).

F378:

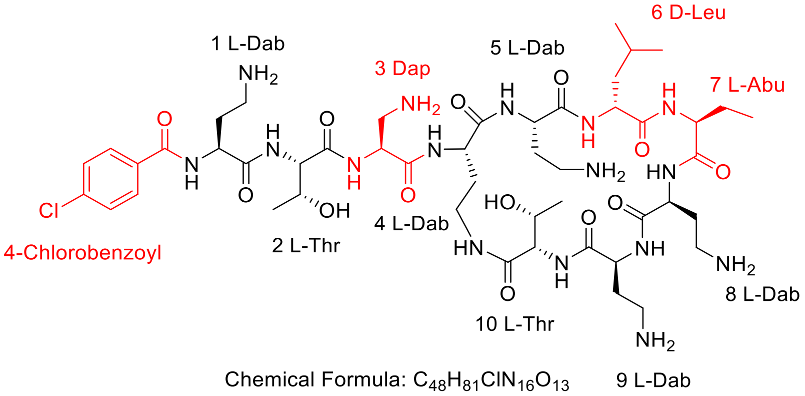

Chemical Formula: $C_{48}H_{81}ClN_{16}O_{13}$

TFA salt was obtained in a yield of 62.1 mg, retention time (t_R) at 214 nm = 10.993 min (HPLC purity: 98.9%). ESI-MS analysis of peak at 10.993 min: *m/z* (monoisotopic) $[M+H]^+$ 1125.8, $[M+2H]^{2+}$ 564.0, $[M+3H]^{3+}$ 376.4. Calculated *m/z* (monoisotopic) for **F378** ($C_{48}H_{81}ClN_{16}O_{13}$) $[M+H]^+$ 1125.6, $[M+2H]^{2+}$ 563.3, $[M+3H]^{3+}$ 375.9. ^{1}H NMR (600 MHz, D_2O) δ 7.82 – 7.65 (m, 2H), 7.57 – 7.42 (m, 2H), 4.51 – 4.37 (m, 2H), 4.32 – 4.08 (m, 7H), 3.51 – 3.43 (m, 1H), 3.35 – 3.22 (m, 2H), 3.18 – 2.88 (m, 9H), 2.33 – 2.06 (m, 7H), 2.03 – 1.77 (m, 4H), 1.69 – 1.48 (m, 4H), 1.19 – 1.09 (m, 6H), 0.93 – 0.78 (m, 9H).

F379:

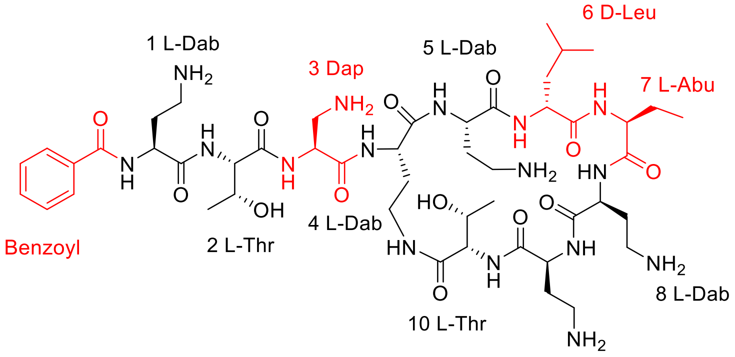

Chemical Formula: $C_{48}H_{82}N_{16}O_{13}$

TFA salt was obtained in a yield of 61.6 mg, retention time (t_R) at 214 nm = 10.497 min (HPLC purity: 99.2%). ESI-MS analysis of peak at 10.497 min: *m/z* (monoisotopic) $[M+H]^+$ 1091.8, $[M+2H]^{2+}$ 546.8, $[M+3H]^{3+}$ 364.9. Calculated *m/z* (monoisotopic) for **F379** ($C_{48}H_{82}N_{16}O_{13}$) $[M+H]^+$ 1091.6, $[M+2H]^{2+}$ 546.3, $[M+3H]^{3+}$ 364.5. ^{1}H NMR (600 MHz, D_2O) δ 7.76 (d, J = 9.2 Hz, 2H), 7.60 (t, J = 7.5 Hz, 1H), 7.49 (t, J = 7.7 Hz, 2H), 4.48 (dd, J = 9.4, 5.0 Hz, 1H), 4.41 (d, J = 3.8 Hz, 1H), 4.14 (d, J = 11.5 Hz, 7H), 3.47 (dd, J = 13.4, 4.9 Hz, 1H), 3.32 – 3.23 (m, 2H), 3.17 – 2.91 (m, 9H), 2.33 – 2.07 (m, 7H), 2.03 – 1.77 (m, 4H), 1.68 – 1.50 (m, 4H), 1.14 (dd, J = 16.8, 6.4 Hz, 6H), 0.89 – 0.85 (m, 6H), 0.83 (d, J = 5.9 Hz, 3H).

F381:

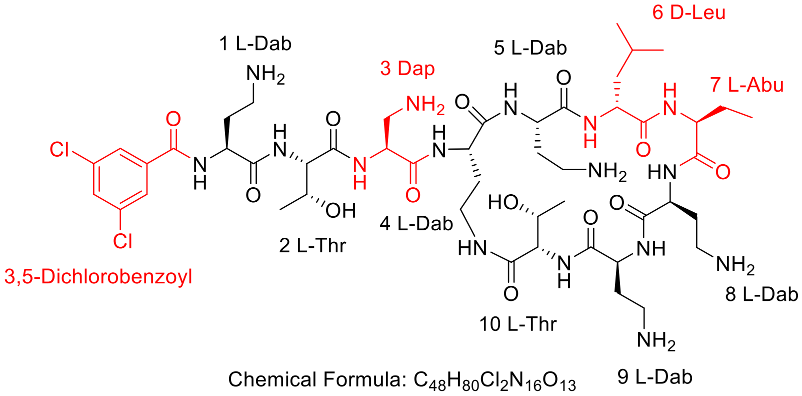

TFA salt was obtained in a yield of 63.8 mg, retention time (t_R) at 214 nm = 11.383 min (HPLC purity: 98.1%). ESI-MS analysis of peak at 11.383 min: *m/z* (monoisotopic) $[M+H]^+$ 1159.8, $[M+2H]^{2+}$ 580.6, $[M+3H]^{3+}$ 387.9. Calculated *m/z* (monoisotopic) for **F381** ($C_{48}H_{80}Cl_2N_{16}O_{13}$) $[M+H]^+$ 1159.5, $[M+2H]^{2+}$ 580.3, $[M+3H]^{3+}$ 387.2. ^{1}H NMR (600 MHz, D_2O) δ 7.70 (d, J = 1.8 Hz, 2H), 7.67 (t, J = 1.8 Hz, 1H), 4.48 (dd, J = 9.4, 5.0 Hz, 1H), 4.41 (d, J = 3.9 Hz, 1H), 4.29 – 4.22 (m, 5H), 4.21 – 4.16 (m, 2H), 4.13 (d, J = 4.6 Hz, 1H), 3.47 (dd, J = 13.4, 4.9 Hz, 1H), 3.27 (dd, J = 13.1, 8.4 Hz, 2H), 3.21 – 2.87 (m, 10H), 2.36 – 2.04 (m, 7H), 2.04 – 1.76 (m, 4H), 1.68 – 1.47 (m, 4H), 1.14 (dd, J = 16.9, 6.4 Hz, 6H), 0.90 – 0.85 (m, 6H), 0.83 (d, J = 5.9 Hz, 3H).

F383:

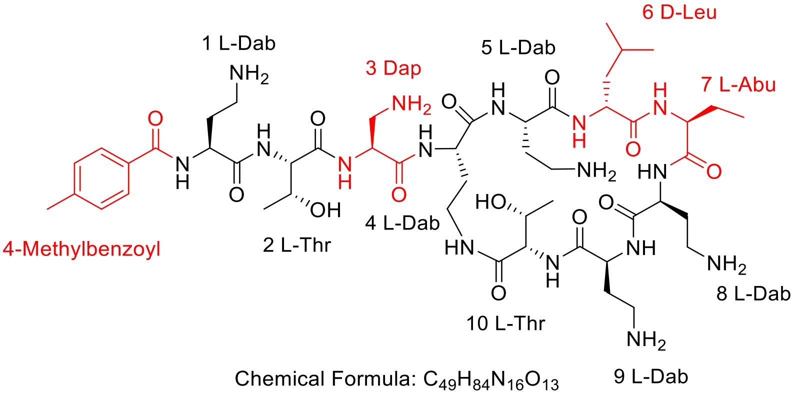

TFA salt was obtained in a yield of 60.0 mg, retention time (t_R) at 214 nm = 10.733 min (HPLC purity: 98.1%). ESI-MS analysis of peak at 10.733 min: *m/z* (monoisotopic) $[M+H]^+$ 1105.9, $[M+2H]^{2+}$ 553.8, $[M+3H]^{3+}$ 369.6. Calculated *m/z* (monoisotopic) for **F383** ($C_{49}H_{84}N_{16}O_{13}$) $[M+H]^+$ 1105.6, $[M+2H]^{2+}$ 553.3, $[M+3H]^{3+}$ 369.2. ^{1}H NMR (600 MHz, D_2O) δ 7.66 (d, J = 8.2 Hz, 2H), 7.32 (d, J = 8.1 Hz, 2H), 4.48 (dd, J = 9.5, 5.0 Hz, 1H), 4.40 (d, J = 3.8 Hz, 1H), 4.30 – 4.15 (m, 6H), 4.13 (d, J = 4.7 Hz, 1H), 3.47 (dd, J = 13.4, 4.9 Hz, 1H), 3.33 – 3.23 (m, 2H), 3.17 – 2.90 (m, 10H), 2.35 (s, 3H), 2.32 – 2.07 (m, 8H), 2.04 – 1.77 (m, 4H), 1.70 – 1.48 (m, 4H), 1.14 (dd, J = 14.7, 6.4 Hz, 6H), 0.90 – 0.85 (m, 6H), 0.83 (d, J = 6.0 Hz, 3H).

F448:

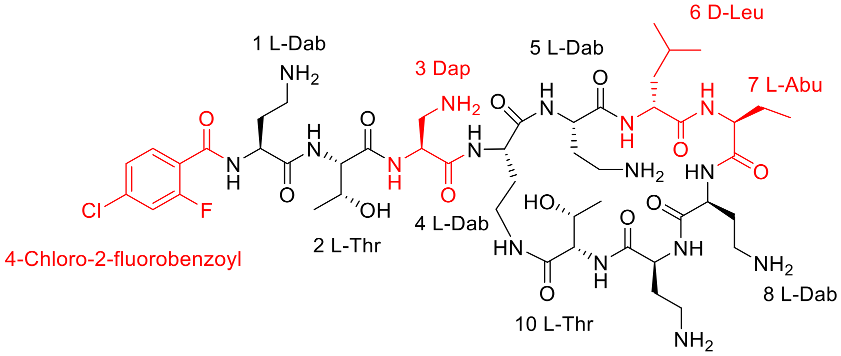

Chemical Formula: $C_{48}H_{80}ClFN_{16}O_{13}$ 9 L-Dab

TFA salt was obtained in a yield of 70.3 mg, retention time (t_R) at 214 nm = 10.727 min (HPLC purity: 97.7%). ESI-MS analysis of peak at 10.727 min: *m/z* (monoisotopic) $[M+H]^+$ 1143.9, $[M+2H]^{2+}$ 572.6, $[M+3H]^{3+}$ 382.4. Calculated *m/z* (monoisotopic) for **F448** ($C_{48}H_{80}ClFN_{16}O_{13}$) $[M+H]^+$ 1143.6, $[M+2H]^{2+}$ 572.3, $[M+3H]^{3+}$ 381.9. ^{1}H NMR (600 MHz, D_2O) δ 7.65 – 7.60 (m, 1H), 7.36 – 7.28 (m, 2H), 4.51 – 4.45 (m, 1H), 4.42 – 4.39 (m, 1H), 4.29 – 4.10 (m, 7H), 3.50 – 3.42 (m,1H), 3.32 – 3.22 (m, 2H), 3.16 – 2.90 (m, 9H), 2.32 – 2.07 (m, 7H), 2.03 – 1.77 (m, 4H), 1.68 – 1.48 (m, 4H), 1.18 – 1.10 (m, 6H), 0.90 – 0.79 (m, 9H).

F449:

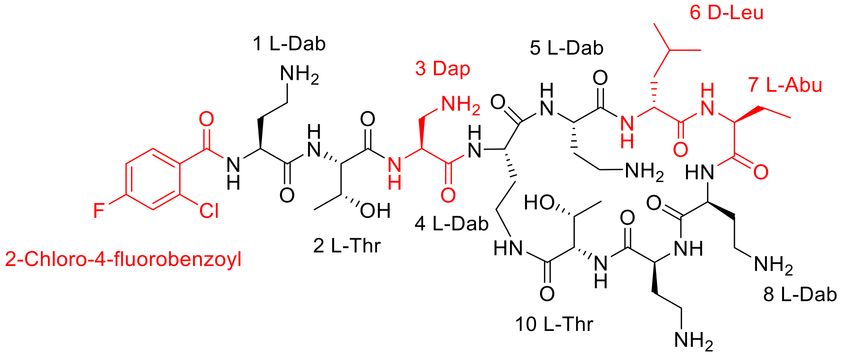

Chemical Formula: $C_{48}H_{80}ClFN_{16}O_{13}$ 9 L-Dab

TFA salt was obtained in a yield of 66.2 mg, retention time (t_R) at 214 nm = 10.476 min (HPLC purity: 98.4%). ESI-MS analysis of peak at 10.476 min: *m/z* (monoisotopic) $[M+H]^+$ 1143.8, $[M+2H]^{2+}$ 572.9, $[M+3H]^{3+}$ 382.4. Calculated *m/z* (monoisotopic) for **F449** ($C_{48}H_{80}ClFN_{16}O_{13}$) $[M+H]^+$ 1143.6, $[M+2H]^{2+}$ 572.3, $[M+3H]^{3+}$ 381.9. ^{1}H NMR (600 MHz, D_2O) δ 7.51 – 7.47 (m, 1H), 7.33 – 7.29 (m, 1H), 7.17 – 7.13 (m, 1H), 4.50 – 4.45 (m, 1H), 4.43 – 4.39 (m, 1H), 4.32 – 4.11 (m, 7H), 3.49 – 3.43 (m, 1H), 3.33 – 3.20 (m, 2H), 3.14 – 3.10 (m, 3H), 3.07 – 2.91 (m, 6H), 2.30 – 2.06 (m, 7H), 2.02 – 1.78 (m, 4H), 1.67 – 1.49 (m, 4H), 1.20 – 1.15 (m, 3H), 1.15 – 1.11 (m, 3H), 0.90 – 0.80 (m, 9H).

F477:

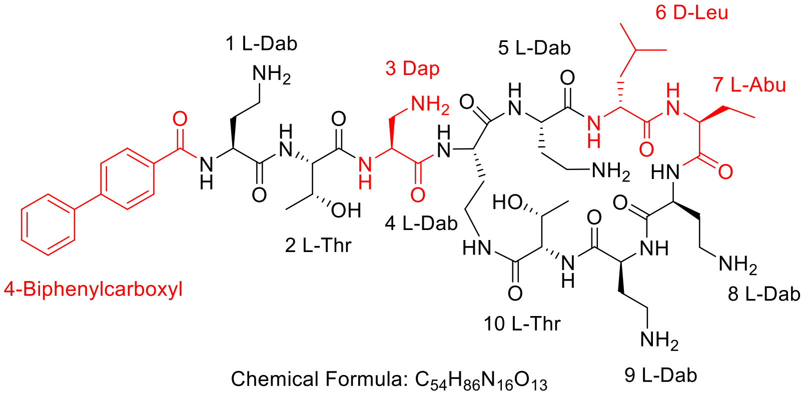

TFA salt was obtained in a yield of 69.3 mg, retention time (t_R) at 214 nm = 11.610 min (HPLC purity: 98.9%). ESI-MS analysis of peak at 11.610 min: *m/z* (monoisotopic) $[M+H]^+$ 1168.0, $[M+2H]^{2+}$ 584.8, $[M+3H]^{3+}$ 390.3. Calculated *m/z* (monoisotopic) for **F477** ($C_{54}H_{86}N_{16}O_{13}$) $[M+H]^+$ 1167.7, $[M+2H]^{2+}$ 584.3, $[M+3H]^{3+}$ 389.9. ^{1}H NMR (600 MHz, D_2O) δ 7.66 – 7.61 (m, 4H), 7.49 – 7.44 (m, 2H), 7.40 – 7.35 (m, 3H), 4.49 – 4.44 (m, 2H), 4.34 – 4.31 (m, 1H), 4.28 – 4.08 (m, 7H), 3.70 – 3.63 (m, 2H), 3.43 – 3.34 (m, 1H), 3.28 – 3.18 (m, 2H), 3.11 – 2.88 (m, 10H), 2.27 – 2.02 (m, 8H), 2.01 – 1.73 (m, 4H), 1.66 – 1.48 (m, 4H), 1.12 – 1.09 (m, 3H), 1.05 – 1.01 (m, 3H), 0.88 – 0.80 (m, 9H).

Scale-up synthesis of F365 (QPX9003). The scale-up synthesis of **F365** was conducted using the protocol described above for the 0.1 mmol scale synthesis; however, it was done in parallel ×6 on the Protein Technologies Prelude automated peptide synthesizer to reach the total scale of 0.6 mmol. The eluents (×6) from the ion-exchange step, were combined. The RP-HPLC purification step was divided into two lots and was carried out on a Waters Prep LC system equipped with a Phenomenex Axia Luna C8(2) column (250 × 50.0 mm i.d., 100 Å, 10 µm). **F365** was eluted from the column (at 214 nm) with a gradient of 0−60% buffer B over 60 min at a flow rate of 40 mL/min; buffer A was 0.1% TFA/water, and buffer B was 0.1% TFA/acetonitrile. The purity of the fractions collected was determined by LC-MS analysis on a Shimadzu 2020 LC-MS system as described above. Fractions containing purified **F365** from the two purifications were combined and lyophilized for 3 days to give **F365** TFA salt as a white powder. **F365** TFA salt was obtained in a yield of 325 mg, retention time (t_R) at 214 nm = 10.820 min (HPLC purity: 99.4%). ESI-MS analysis of peak at 10.820 min: *m/z* (monoisotopic) $[M+H]^+$ 1160.0, $[M+2H]^{2+}$ 580.7, $[M+3H]^{3+}$ 387.9 Calculated *m/z* (monoisotopic) for **F365** ($C_{48}H_{80}Cl_2N_{16}O_{13}$) $[M+H]^+$ 1159.5, $[M+2H]^{2+}$ 580.3, $[M+3H]^{3+}$ 387.2.
