# Supplementary material for: A synthetic lipopeptide targeting top-priority multidrug-resistant Gram-negative pathogens
Source: Nat Commun. 2022 Mar 25;13:1625. doi: 10.1038/s41467-022-29234-3 (PMC8956739; doi:10.1038/s41467-022-29234-3)
Supplement: Supplementary file 2 — Reporting Summary [file 41467_2022_29234_MOESM2_ESM.pdf]

nature portfolio

Corresponding author(s): Tony Velkov and Jian Li

Last updated by author(s): Feb 17, 2022

Reporting Summary

Nature Portfolio wishes to improve the reproducibility of the work that we publish. This form provides structure for consistency and transparency in reporting. For further information on Nature Portfolio policies, see our Editorial Policies and the Editorial Policy Checklist.

Statistics

For all statistical analyses, confirm that the following items are present in the figure legend, table legend, main text, or Methods section.

n/a	Confirmed	
☐	☒	The exact sample size (*n*) for each experimental group/condition, given as a discrete number and unit of measurement
☐	☒	A statement on whether measurements were taken from distinct samples or whether the same sample was measured repeatedly
☐	☒	The statistical test(s) used AND whether they are one- or two-sided<br>*Only common tests should be described solely by name; describe more complex techniques in the Methods section.*
☒	☐	A description of all covariates tested
☐	☒	A description of any assumptions or corrections, such as tests of normality and adjustment for multiple comparisons
☐	☒	A full description of the statistical parameters including central tendency (e.g. means) or other basic estimates (e.g. regression coefficient) AND variation (e.g. standard deviation) or associated estimates of uncertainty (e.g. confidence intervals)
☐	☒	For null hypothesis testing, the test statistic (e.g. *F*, *t*, *r*) with confidence intervals, effect sizes, degrees of freedom and *P* value noted<br>*Give P values as exact values whenever suitable.*
☒	☐	For Bayesian analysis, information on the choice of priors and Markov chain Monte Carlo settings
☒	☐	For hierarchical and complex designs, identification of the appropriate level for tests and full reporting of outcomes
☒	☐	Estimates of effect sizes (e.g. Cohen's *d*, Pearson's *r*), indicating how they were calculated

Our web collection on statistics for biologists contains articles on many of the points above.

Software and code

Policy information about availability of computer code

Data collection	Nil.
Data analysis	Open source software subread 2.0.1 was used for RNA-Seq reads alignment. R packages limma 3.46.0, mixOmics 6.14.1 and enrichR 2.1 were used for identification of differentially expressed genes, principle component analysis and HK-2 enrichment analysis, respectively. Packages UpSetR 1.4.0, CompleHeatmap 2.6.2, circlize 0.4.13 and iGraph 1.2.11 were used for data visualisation. Custom R codes are available at https://doi:10.5281/zenodo.5915369.

For manuscripts utilizing custom algorithms or software that are central to the research but not yet described in published literature, software must be made available to editors and reviewers. We strongly encourage code deposition in a community repository (e.g. GitHub). See the Nature Portfolio guidelines for submitting code & software for further information.

Data

Policy information about availability of data

All manuscripts must include a data availability statement. This statement should provide the following information, where applicable:

- Accession codes, unique identifiers, or web links for publicly available datasets
- A description of any restrictions on data availability
- For clinical datasets or third party data, please ensure that the statement adheres to our policy

Reference genomes of AB5075 (GCF_000963815) and human (GRCh38.94) were downloaded from RefSeq and Ensembl database, respectively. Transcriptomics data were deposited in Short Reads Archive database and publicly accessible under accession numbers SRR15235669-SRR15235725 and SRR15239061-SRR15239078.
BioCyc Acinetobacter baumannii strain AB5075 database (version 25.5) was used for AB5075 enrichment analysis. Key regulatory genes of HK-2 were analyzed in

the context of signalling network in Signor 2.0 database. Source data are provided with this paper.

Field-specific reporting

Please select the one below that is the best fit for your research. If you are not sure, read the appropriate sections before making your selection.

☒ Life sciences ☐ Behavioural & social sciences ☐ Ecological, evolutionary & environmental sciences

For a reference copy of the document with all sections, see nature.com/documents/nr-reporting-summary-flat.pdf

Life sciences study design

All studies must disclose on these points even when the disclosure is negative.

Sample size	Isolates from each of the three target bacterial species were chosen, comprising of reference strains (from the American Type Culture Collection) and multidrug-resistant clinical isolates. These sample sizes were sufficient for the initial screening and large-panel MIC measurements. Considering statistical analysis, n => 3 was employed in each group/time point for all the in vitro and in vivo experiments. The numbers of samples are 57 and 18 for the RNA-Seq experiments of Acinetobacter baumannii AB5075 and HK-2 cells, respectively.
Data exclusions	No data were excluded.
Replication	All data were replicated in at least two replicates. All attempts at replication were successful for MIC experiments. The data from the mouse lung infection model were repeated once or multiple times and the data were consistent. Pharmacokinetic experiments were conducted in >=3 animals for each condition and the LC-MS/MS analysis for all the mouse and rat samples were conducted with appropriate quality control samples (n>=3) and acceptable accuracy and reproducibility.
Randomization	The randomization principle was applied in all the steps of animal experiments, including assigning animals into each treatment group. In the LC-MS/MS analysis procedures, the randomization principle was also applied, e.g. arranging the order of sample vials on the LS-MS/MS racks.
Blinding	The blinding principle was applied whenever applicable. In the animal experiments, the investigator were blinded to group allocation during data collection and/or analysis; for example, the experimenter for bacterial counting was blinded to the treatment details, and the histologist was blinded to the drug treatment details.

Reporting for specific materials, systems and methods

We require information from authors about some types of materials, experimental systems and methods used in many studies. Here, indicate whether each material, system or method listed is relevant to your study. If you are not sure if a list item applies to your research, read the appropriate section before selecting a response.

Materials & experimental systems

n/a	Involved in the study	
☒	☐	Antibodies
☐	☒	Eukaryotic cell lines
☒	☐	Palaeontology and archaeology
☐	☒	Animals and other organisms
☒	☐	Human research participants
☒	☐	Clinical data
☒	☐	Dual use research of concern

Methods

n/a	Involved in the study	
☒	☐	ChIP-seq
☒	☐	Flow cytometry
☒	☐	MRI-based neuroimaging

Eukaryotic cell lines

Policy information about cell lines

Cell line source(s)	the American Type Culture Collection (ATCC)
Authentication	HK-2 cell line was purchased from the ATCC and cultured as per the ATCC recommendations.
Mycoplasma contamination	We confirm that the cell line we used had no Mycoplasma contamination.
Commonly misidentified lines (See ICLAC register)	No commonly misidentified cell lines were used this study.

Animals and other organisms

Policy information about studies involving animals; ARRIVE guidelines recommended for reporting animal research

Laboratory animals	In the mouse acute toxicity, bloodstream infection, thigh infection, lung infection, nephrotoxicity and pharmacokinetics studies, Swiss mice (female, 7 weeks old) were used. In the rat pharmacokinetics study, Sprague-Dawley rats (male, 10 weeks old) were used. In the monkey GLP-toxicity study, Cynomolgus monkeys (male and female, 2 to 4 years old) were used. Mice were housed in micro-isolators in a PC2 animal laboratory with 12h/12h (6pm, 6am) dark/light cycle; the room temperature was controlled between 20 and 24˚C; and a relative ambient humidity was adjusted to 50-70%.
Wild animals	No wild animal were used.
Field-collected samples	No field samples were used.
Ethics oversight	All animal studies conducted by Monash University and Qpex Biopharma were approved by the Monash Animal Ethics Committee and Qpex Institutional Animal Care and Use Committee, respectively.

Note that full information on the approval of the study protocol must also be provided in the manuscript.
